# Supplementary material for: Can smartphone technology be used to support an effective home exercise intervention to prevent falls amongst community dwelling older adults?: the TOGETHER feasibility RCT study protocol
Source: BMJ Open. 2019 Sep 18;9(9):e028100. doi: 10.1136/bmjopen-2018-028100 (PMC6756425; doi:10.1136/bmjopen-2018-028100)
Supplement: Supplementary data [file bmjopen-2018-028100supp003.pdf]

Supplementary Material Table 1: Intervention description using the Template for Intervention Description and Replication (TIDieR) checklist*

<table>
<tr><td>Item 1: Name</td><td colspan="2">Can smartphone TechnolOGy be used to support an EffecTive Home ExeRcise intervention to prevent falls amongst community dwelling older people: The TOGETHER trial</td></tr>
<tr><td>Item 2: Why</td><td colspan="2">Strength and balance training has been found to be effective in reducing the rate and risk of falls. Health services are often unable to deliver the evidence-based dose of exercise and older adults do not always sufficiently adhere to their programme to gain full outcomes. This feasibility trial will explore whether smartphone technology based on the Theory of Planned Behaviour and using goal setting, feedback and prompts can be used to support patients to better adhere to an evidence-based exercise rehabilitation programme and test study procedures and outcome measures.</td></tr>
<tr><td rowspan="2">Item 3: What (Materials)</td><td>Intervention arm: Behaviour change apps</td><td>Control arm:
Standard service</td></tr>
<tr><td>Samsung Galaxy J5

Health Professional phone based Motivate Me app: -
1. set patients long-term goals (outcomes)
2. set patients behavioural goals (which evidence based exercises they will do and how often, when they will exercise with the health professional, alone or in a group),
3. access the patients self-report data and see what exercises they have been doing and when.
4. upgrade exercise programme.
5. give the patient bespoke feedback (set as once a week).

Patient phone based My Activity Programme app:-
1. report the exercises they have done (exercise type, duration, intensity)
2. receive prompts when they have scheduled to exercise
3. receive automated motivational messages based on the long-term goals they have set (with a focus on strengthening outcome</td><td>Samsung Galaxy J5

Control group self-report app:-
report the exercises they have done (exercise type, duration, intensity)

Home exercise booklet

Calander/FallsMonitor@home

Patient ‘How to guide’

Technology issue log (Health professional)</td></tr>
</table>

	expectations)<br>4. Receive bespoke feedback from health professionals (aimed at increasing Perceived Behavioural Control).<br><br>Home exercise booklet<br><br>Calander/FallsMonitor@home<br><br>Patient ‘How to guide’<br>Health professional ‘How to guide’ (for technology)<br><br>Technology issue log (Health professional)	
**4. What procedure**	Screening and assessment as part of standard service by Falls Services<br><br>Support and training on how to use the ‘My Activity Programme app’ and falls alarm.<br><br>Formal goal setting session with health professional where use their ‘Motivate me’ app to set patients’ behavioural and outcome-based goals (including prescribing the exercise programme). Handing out of home exercise booklet.<br><br>Strength and balance rehabilitation in group or home commences.<br><br>**Trafford:**<br>If recruited through Community Rehabilitation team they receive a 6- week programme. They are then either referred to group-based rehabilitation or discharged. Seen in own home once a week.<br><br>If recruited through group-based rehabilitation they receive an 8 week group-based programme. Seen in a group once a week.<br><br>They are then referred on to a community based FaME class once a week and prescribed home	Screening and assessment as part of standard service by Falls Services.<br><br>Some discussion around long term goals and home exercise booklet given and programme prescribed (behavioural goals).<br><br>Strength and balance rehabilitation in group or home commences.<br><br>**Trafford:**<br>If recruited through Community Rehabilitation team they receive a 6 week programme. They are then either referred to group-based rehabilitation or discharged. Seen in own home once a week.<br><br>If recruited through the group-based rehabilitation they receive an 8 week group-based programme. Seen in a group once a week.<br><br>They are then referred on to a community based FaME class once a week and prescribed home exercise programme.<br><br>**Manchester City**<br>They receive either a 12 week group based exercise class and prescribed home exercise programme or just a 12 week home based exercise, and

	exercise programme.<br><br>**Manchester City**<br>They receive either a 12 week group based exercise class and prescribed home exercise programme or just a 12 week home based exercise, and then follow-up visits until 6 months (as per patient need). On discharge they are referred to the exercise referral programme where they can attend a group once a week and receive a prescribed home exercise programme. Sometimes they are referred to exercise referral programme before discharge.<br><br>All patients across both sites will receive a prescribed programme by the health professional on discharge and the health professional will set behavioural goals with the patient using the apps. Throughout the rehabilitation period the health professional can upgrade the exercise programme as appropriate using the apps. They will also send the patient a personalised motivational message once a week until discharge.<br><br>Participants receive a visit from the research team the week after their rehabilitation programme has started and then monthly phonecalls.<br><br>Participants can contact the research team for technical support at any time.	then follow-up visits until 6 months (as per patient need). On discharge they are referred to the exercise referral programme where they can attend a group once a week and receive a prescribed home exercise programme. Sometimes they are referred to the exercise referral programme before discharge.<br><br>All patients across both sites will receive a prescribed programme by the health professional on discharge.<br><br>Participants receive a visit from the research team the week after their rehabilitation programme has started and then monthly phonecalls.<br><br>Participants can contact the research team for technical support at any time.
**Item 5: who provided**	**Assessments**<br>All physical assessments completed at the clinical sites are completed by blinded clinical staff from those sites. All assessors are qualified physiotherapists.<br>Assessments are completed at baseline (prior to randomisation), 3 and 6 months. Other assessments are self- completed by the older person.<br><br>**Intervention**<br>Both the intervention and control are delivered by the same health	

<table>
<tr><td></td><td colspan="3">professionals at both sites. These are predominantly Physiotherapists, but also Occupational Therapists and Healthcare Assistants. All health professionals have been trained to deliver the evidence based rehabilitation programme and have also received training on using the smartphone from the research team.</td></tr>
<tr><td rowspan="5">6. How</td><td>Identification</td><td colspan="2">Patients are identified by health professionals at 2 sites and screened against the inclusion/exclusion criteria. They are then approached by the health professional and asked if they would like a demo of the technology.</td></tr>
<tr><td>Consent</td><td colspan="2">The researcher contacts the individual and arranges a visit accompanied by a former patient who has used the smartphone app before.
The researcher takes consent.</td></tr>
<tr><td rowspan="3">Exercise delivery

Motivational input</td><td colspan="2">Exercise delivery is the same across intervention and control, but differs dependent on site (see 4.What). Exercise is delivered by the health professional either in the home or within a group.</td></tr>
<tr><td>INTERVENTION</td><td>CONTROL</td></tr>
<tr><td>Patient carry out goal setting session with health professional structured by the use of the ‘Motivate Me’ app and receive a prescribed programme on their app and an exercise booklet.

Patient will receive prompts and messages through the ‘my activity programme app’ at home on the days they have planned to exercise.

Patients will also receive verbal feedback when they see the health professional.</td><td>Patient given a prescribed exercise programme and some informal goal setting is carried out as part of the session. They receive a home exercise booklet.

Patients will only receive verbal feedback when they see the health professional</td></tr>
<tr><td>7. Where</td><td colspan="3">The intervention is delivered across several community venues and health centres venues and in patients’ homes in Manchester City and Trafford.</td></tr>
<tr><td rowspan="3">8. When and how much</td><td></td><td>Intervention arm</td><td>Control arm</td></tr>
<tr><td></td><td></td><td></td></tr>
<tr><td>Research Team</td><td>1 visit to set- up the phone after randomisation</td><td>1 visit to set- up the phone after randomisation</td></tr>
</table>

		1 visit the week after the rehabilitation programme has commenced<br><br>Phone calls x 6	1 visit the week after the rehabilitation programme has commenced<br><br>Phone calls x 6
	Health Professional	2 x structured goal setting session (baseline and at discharge).<br><br>Weekly face to face exercise intervention (ranging from 6-15 contacts).<br><br>Weekly feedback message received through app until discharge (maximum of 26).	2 x goal setting session (baseline and at discharge).<br><br>Weekly face to face exercise intervention (ranging from 6-15 contacts).
	Smartphone	Automated messages and prompts x 3 on the day they have scheduled to exercise.	
**9. Tailoring**	Rehabilitation programme	The number of home based visits to each patient may differ across both recruitment sites and across both control and intervention groups dependent on patient need.<br><br>The exercise programme delivered will be tailored to each individual patient across both sites and across both control and intervention groups.<br><br>Both sites will send the weekly feedback message until patient discharge (this could be at 8 weeks at Trafford and 6 months at Manchester city).	
	Motivational messages	Because the exercise programme is tailored to the individual, health professionals may schedule for the patient to exercise 3 times a week on the phone or every day, dependent on preference. Participants will receive messages on the days they have scheduled to exercise some patients may receive more than others.	
**11. How well planned**	Health professional fidelity	The research team will attend the goal-setting sessions for the intervention arm for the first 5 patients at each site and then 1 patient at each site every 2 months.<br><br>All staff have undergone a half-day training session and a follow-up support session in using the smartphone app and trial procedures.	

	Assessment fidelity	Assessors masked to group allocation will follow an assessment standard operating procedure.
	Adherence	1. Adherence is collected through the smartphone apps (control and intervention).<br>2. At baseline, 3 and 6 months though validated questionnaire (EARS).<br>3. Through group exercise attendance records, health professional and instructor delivery records.

Item 10 and 12 N/A.
