# Supplementary figures and images for: Can smartphone technology be used to support an effective home exercise intervention to prevent falls amongst community dwelling older adults?: the TOGETHER feasibility RCT study protocol
Source: BMJ Open. 2019 Sep 18;9(9):e028100. doi: 10.1136/bmjopen-2018-028100 (PMC6756425; doi:10.1136/bmjopen-2018-028100)

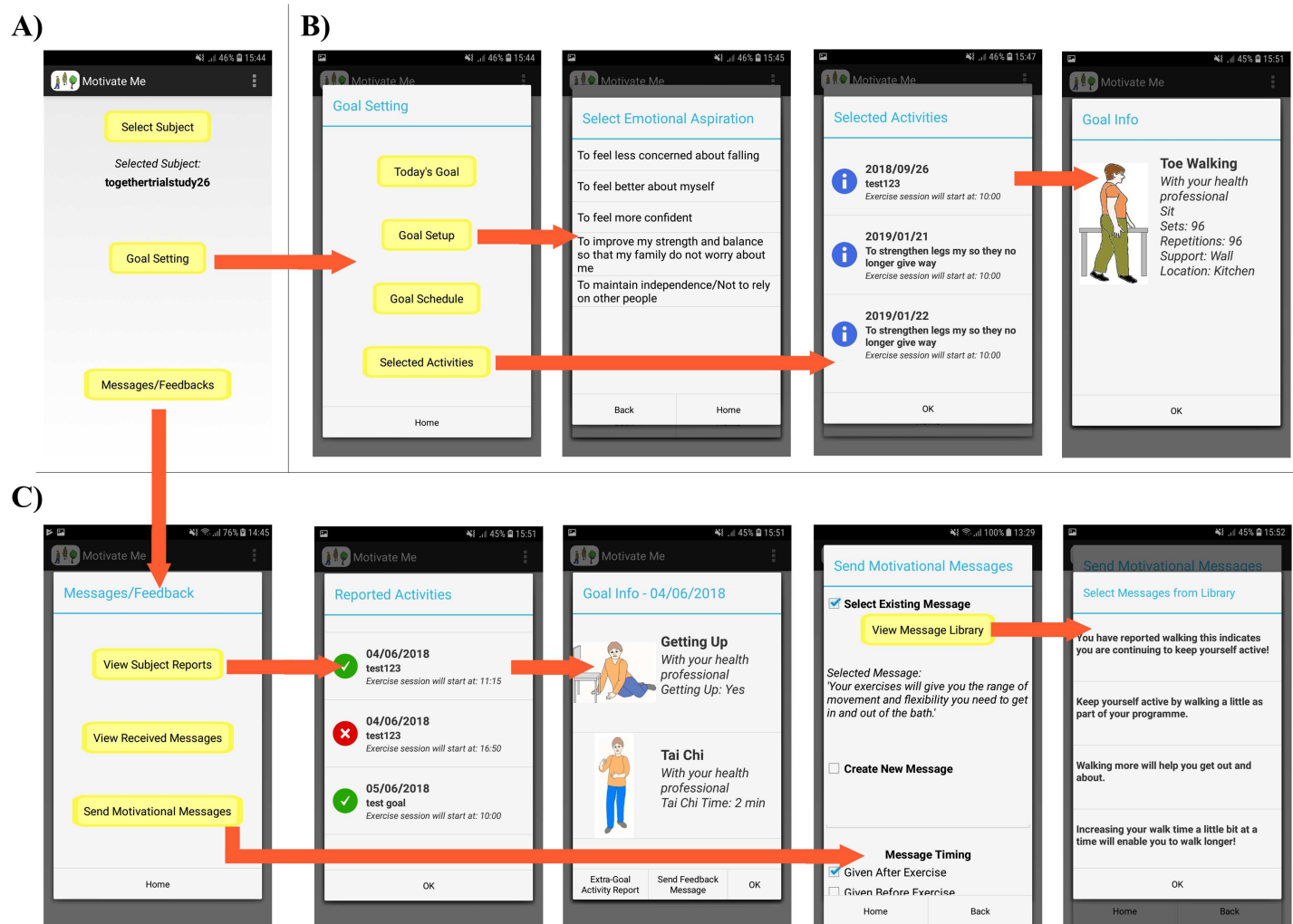

Supplement: Supplementary data [file bmjopen-2018-028100supp001.pdf]

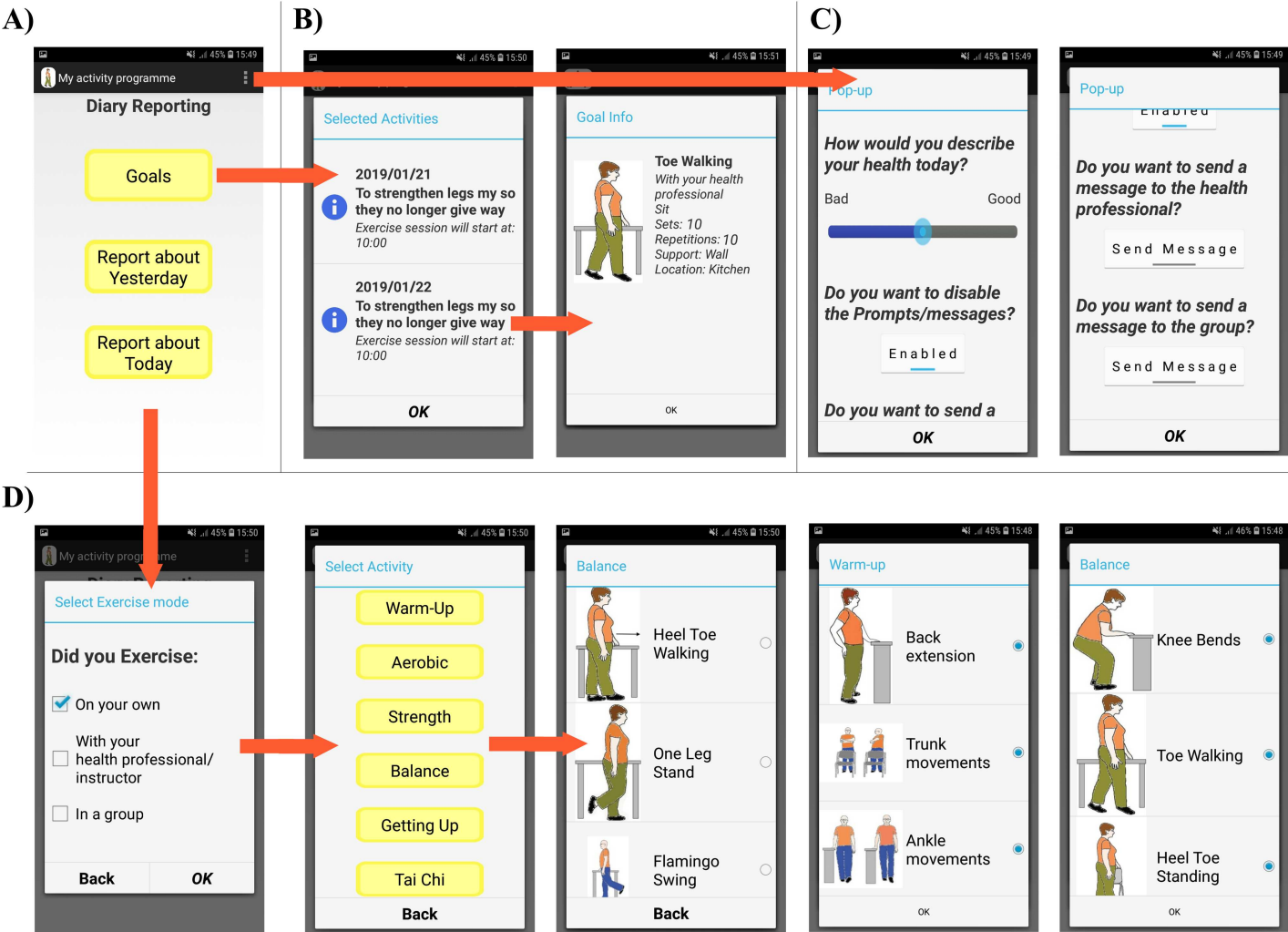

Supplement: Supplementary data [file bmjopen-2018-028100supp002.pdf]
