# Supplementary material for: Can smartphone technology be used to support an effective home exercise intervention to prevent falls amongst community dwelling older adults?: the TOGETHER feasibility RCT study protocol
Source: BMJ Open. 2019 Sep 18;9(9):e028100. doi: 10.1136/bmjopen-2018-028100 (PMC6756425; doi:10.1136/bmjopen-2018-028100)
Supplement: Author's manuscript [file bmjopen-2018-028100.draft_revisions.pdf]

BMJ Open is committed to open peer review. As part of this commitment we make the peer review history of every article we publish publicly available.

When an article is published we post the peer reviewers' comments and the authors' responses online. We also post the versions of the paper that were used during peer review. These are the versions that the peer review comments apply to.

The versions of the paper that follow are the versions that were submitted during the peer review process. They are not the versions of record or the final published versions. They should not be cited or distributed as the published version of this manuscript.

BMJ Open is an open access journal and the full, final, typeset and author-corrected version of record of the manuscript is available on our site with no access controls, subscription charges or pay-per-view fees (http://bmjopen.bmj.com).

If you have any questions on BMJ Open's open peer review process please email editorial.bmjopen@bmj.com

BMJ Open

Can smartphone TechnolOGy be used to support an EffecTive Home ExeRcise intervention to prevent falls amongst community dwelling older adults? The TOGETHER feasibility RCT study protocol.

Journal:	*BMJ Open*
Manuscript ID	bmjopen-2018-028100
Article Type:	Protocol
Date Submitted by the Author:	21-Nov-2018

Complete List of Authors:	Hawley-Hague, Helen; University of Manchester, School of Health Sciences<br>Tacconi, Carlo; Universita degli Studi di Bologna Dipartimento di Ingegneria dell'Energia Elettrica e dell'Informazione Guglielmo Marconi<br>Mellone, Sabato; University of Bologna, Department of Electrical, Electronic and Information Engineering «Guglielmo Marconi»<br>Martinez, Ellen; Central Manchester University Hospitals NHS Foundation Trust<br>Easdon, Angela; Pennine Care NHS Foundation Trust<br>Yang, Fan; University of York, Centre for Health Economics<br>Su, Ting-Li; University of Manchester, Dentistry<br>Mikolaizak, A. Stefanie; Robert Bosch Krankenhaus<br>Chiari, Lorenzo; University of Bologna, Department of Electrical, Electronic and Information Engineering «Guglielmo Marconi»<br>Helbostad, Jorunn<br>Todd, Chris; University of Manchester, School of Health Sciences

Keywords:	REHABILITATION MEDICINE, older, mhealth

SCHOLARONE™
Manuscripts

Can smartphone **T**echnol**OG**y be used to support an **E**ffec**T**ive **H**ome **E**xe**R**cise intervention to prevent falls amongst community dwelling older adults? The **TOGETHER** feasibility RCT study protocol.

Hawley-Hague, H[1]., Tacconi, C[2,3]., Mellone, S[2,3,4]., Martinez, E[5], Easdon, A[6]., Yang, F[7]., Su, T[8]., Mikolaizak, A.S[9]., Chiari, L[2,3,4]., Helbostad, J[10]., Todd[1, 11], C.

[1] School of Health Sciences, Faculty of Biology, Medicine and Health, The University of Manchester and Manchester Academic Health Sciences Centre, Manchester, U.K.
[2] Health Sciences and Technologies-Interdepartmental Center for Industrial Research, University of Bologna, Bologna, Italy.
[3] mHealth Technologies s.r.l., Bologna, Italy,
[4] Department of Electrical, Electronic and Information Engineering «Guglielmo Marconi» - University of Bologna, Bologna, Italy.
[5] Manchester University NHS Foundation Trust, Manchester, U.K
[6] Pennine Care Hospital Trust, Manchester, U.K
[7] Centre for Health Economics, University of York, York, U.K
[8] School of Medical Sciences, Faculty of Biology, Medicine and Health, The University of Manchester, Manchester, U.K
[9] Robert Bosch Krankenhaus, Department of Clinical Gerontology, Stuttgart, Germany
[10] Department of Neuromedicine and Movement Science, The Faculty of Medicine and Health Sciences, The Norwegian University of Science and Technology, NTNU, Trondheim, Norway
[11] Manchester University NHS Foundation Trust, Manchester, U.K.

Corresponding author:
Dr Helen Hawley-Hague
The University of Manchester
School of Health Sciences (Nursing, Midwifery and Social Work)
Floor 6, Jean McFarlane Building
Oxford Road
Manchester
M13 9PL
Helen.Hawley-Hague@manchester.ac.uk

Word count: 4,231

For peer review only

ABSTRACT

Introduction

Falls have major implications for quality of life, independence and cost to the health service. Strength and balance training has been found to be effective in reducing the rate/risk of falls, as long as there is adequate fidelity to the evidence-based programme. Health services are often unable to deliver the evidence-based dose of exercise and older adults do not always sufficiently adhere to their programme to gain full outcomes. Smartphone technology based on behaviour-change theory has been used to support healthy lifestyles, but not falls prevention exercise. This feasibility trial will explore whether smartphone technology can support patients to better adhere to an evidence-based rehabilitation programme and test study procedures/outcome measures.

Methods and analysis

A two-arm, pragmatic simple randomised controlled trial will be conducted with health services in Manchester, UK. Seventy-two patients aged 50+ years eligible for a falls rehabilitation exercise programme from two community services will receive: (1) standard service or (2) standard service plus a motivational smartphone app. The primary outcome is feasibility and acceptability of the intervention, design and procedures. Secondary outcome measures include balance, function, falls, strength, fear of falling, health related quality of life, resource use and adherence. Outcomes are measured at baseline, three and six month post-randomisation. Interviews/focus groups with health professionals and participants further explore feasibility of the technology and trial procedures. Primarily analyses will be descriptive.

Ethics and dissemination.

The study protocol is approved by North West Greater Manchester East Research Ethics Committee (Rec ref:18/NW/0457, 9/07/2018). User groups and patient representatives were consulted to inform trial design, and are involved in study recruitment. Results will be reported at conferences and in peer-reviewed publications. A dissemination event will be held in Manchester to present the results of the trial. The protocol adheres to the recommended SPIRIT Checklist.

Trial registration number: ISRCTN12830220

Key words: Rehabilitation, older, technology

Strengths and limitations of this study

- The first study to examine a motivational app to support falls rehabilitation.

For peer review only
For peer review only - http://bmjopen.bmj.com/site/about/guidelines.xhtml

- Pragmatic feasibility trial enabling results to be made directly applicable to practice.
- Multi-site study with different types of falls services, representative of UK service delivery.
- Standard service differs across the two sites, making the study more complex.
- Due to the nature of the intervention we will not be able to blind participants or those delivering the intervention.

For peer review only

Falls are an important public health issue, with over 30% of people aged 65 and over falling at least once a year[1]. This has implications for quality of life, independence and cost to the health service[1]. Strength and balance training (SBT) comprises 'carrying out exercises that increase muscle strength in the legs and improve balance'[2]. Strength and balance exercise programmes are effective in reducing risk and rate of falls and injuries[3]. Sherrington et al[4] have shown that for strength and balance programmes to be effective they need to be progressive, tailored and of adequate dose (3x a week for 50 hours, and then maintained). Work carried out by Public Health England[5] illustrates that to see a return on investment; fidelity to the evidence-base has to be carried-out (adequate dose, progression).

However, Nyman and Victor[6] report that adherence to evidence-based strength and balance programmes is poor. The National Health Service (NHS) only delivers programmes that are pre-dominantly 3 months or less[7], older adults do not carry out their exercise programme three times a week as prescribed (dose) or carry out the programme for a sufficient length of time to achieve and maintain the benefits[6,7]. Cost and appropriate staffing are cited as primary reasons for short NHS delivery[7].

Unless there are innovative new solutions to support the delivery of falls prevention exercises sufficiently to reduce falls risk and to prevent re-referral to services, over the coming decade it is estimated that population changes will result service demand beyond the reach of current interventions[8].The use of smartphone to support falls rehabilitation could be one of the solutions. The evidence which looks at the role of the smartphone for falls prevention is sparse[9], particularly for interventions focused on rehabilitation/strength and balance training. Although, there is a lack of specific evidence related to falls prevention interventions, there is evidence that older adults find mobile phones more usable than using a new device e.g. a falls alarm[10]. There is also evidence supporting the use of mobile phone-based healthy lifestyle programmes [11,12], including to increase physical activity[13,14]. King et al[15], developed and tested apps based on behaviour change theory designed to motivate adults aged 45+ years. One of these included personalised goal-setting and behavioural feedback, successful evidence-based behavioural change techniques[16]. The app recieved positive feedback from participants and increased physical activity.

We know from previous studies that attitudes and beliefs are important to the uptake of and adherence to exercise by older adults[17,18]. The Theory of Planned Behaviour (TPB) [19] is particularly useful for assessing older adults' attitudes in relation to exercise uptake and adherence [17,18,20]. The TPB is based on three core components:
(i) perceived behavioural control (PBC), the perceived ease or difficulty of performing the behaviour.
(ii) social influences including, subjective norms (beliefs of important people e.g. family), perceived social support (support from others for behaviour) and modelling (following observed behaviour of others).
(iii) attitudes (outcome expectations)[19]. Focused on the advantages and disadvantages of the behaviour (outcome expectations) and when related to adherence, whether these advantages have occurred.

Attitudes measured by using a TPB-based tool have been significantly associated with exercise behaviour in a previous study[17]. This theory has informed the intervention

For peer review only - http://bmjopen.bmj.com/site/about/guidelines.xhtml

overall and content of the motivational messages within the proposed intervention (focused on outcome expectations/PBC).

Smartphone technology-based motivational applications underpinned by behaviour change theory and developed with health professionals and older adults could be an effective way of encouraging maintenance of exercise and of successfully supporting adherence to evidence-based strength and balance training. We have already carried out usability and acceptability testing of the technology and applications, before planning this trial (ISRCTN: 12830220). The smartphone apps have been developed through several cycles of user-led design. Initially we carried out engagement workshops with older adults (AgeUK) and health professionals from one falls service in Manchester, followed by usability/acceptability testing with another falls service in Manchester and their patients (IRAS:205980). The use of this approach has enabled us to develop the apps, establish whether the technology is acceptable to older adults and health professionals (qualitative methods) and to check its usability (technology testing). This study now aims to explore whether it is feasible for smartphone technology to be used to support patients to sufficiently adhere to an evidence-based exercise rehabilitation programme. As a secondary aim it will assess whether technology-based outcome measures (smartphone-based falls alarm and Timed up and Go Test)[21] are reliable. Through a feasibility RCT we will explore the feasibility of using smartphone technology to support falls rehabilitation and test study procedures (e.g. suitability of outcome measures, standard deviation of the outcome measure, recruitment, randomisation, follow-up rates, retention, time required for analysis). Both arms of the trial will receive rehabilitation exercises and will report their exercises on the phone but only the intervention arm will carry out goal-setting, receive feedback.

The intervention has the potential to:
1. Increase the amount of support the patient receives to adhere to their exercise, leading to increased adherence.
2. Increase exercise progression/dose which could be cost neutral/saving.
3. Enable health professionals to monitor compliance to the prescribed programme.
This could assist maintenance of health, reducing long-term falls risk and re-access to services.

METHODS

Trial design

Core trial information is presented in Table 1. This study is a two-arm pragmatic feasibility randomised controlled trial including economic analysis. The trial design framework is exploratory. Alongside the trial, qualitative work is carried out to understand the acceptability and feasibility of the intervention and the trial procedures.

For peer review only

Table 1: WHO Trial Registration Data Set

**Data category**	**Information**
Primary registry and trial identifying number	ISRCTN
Date of registration in primary registry	21.08.2018
Secondary identifying numbers	
Source of monetary or material support	National Institute for Health Research Postdoctoral Fellowship Award
Primary sponsor	University of Manchester
Secondary sponsor	N/A
Contact for public queries	Helen.hawley-hague@manchester.ac.uk
Contact for scientific queries	Helen.hawley-hague@manchester.ac.uk
Public title	The Together trial
Scientific Title	Can smartphone **T**echnol**OG**y be used to support an **E**ffec**T**ive **H**ome **E**xe**R**cise intervention to prevent falls amongst community dwelling older people?<br>The **TOGETHER** feasibility RCT.
Countries of recruitment	UK
Health condition of problem studied	Falls in Older Adults
Interventions	**Standard service:**<br>**Manchester City**: 12 weeks once a week visits (either home based or group exercise) and then check-ups until 6 months discharge. Prescribed exercise plan and exercise booklet given, asked informally what they want to achieve (outcome goals).<br>**Trafford:** 8 weeks group exercise once a week then discharged. Prescribed exercise plan and exercise booklet given, asked informally what they want to achieve (outcome goals).<br>OR 6 week home based exercise then discharged or referred to further 8 week group exercise. Prescribed exercise plan and exercise booklet given, asked informally what they want to achieve (outcome goals).

For peer review only - http://bmjopen.bmj.com/site/about/guidelines.xhtml

	**Intervention:** Standard service plus the use of Motivate Me and My Activity Programme applications.
Key inclusion and exclusion criteria	**Age:** Older adults aged 50+<br>**Sex:** Male or female<br>**Inclusion:** At risk of falls, referred to falls rehabilitation services and assessed as suitable for an exercise programme, Good 3G/4G reception in their home or wifi.<br>**Exclusion:** unable to follow instructions (unless they have support from a family member or carer), Severe visual impairment, long-term residential or nursing care, terminal illness or expected shortened lifespan, defined as less than 6 months, Older adults unable to read written English unless they have support from a family member or carer).
Study type	Interventional<br>Allocation: simple randomised;<br>Primary purpose: prevention, feasibility<br>Phase II clinical trial
Date of first enrolment:	September 2018
Target sample size	72
Recruitment status	Pending
Primary outcome	Feasibility and acceptability of the design and procedures
Key secondary outcomes	Balance (Berg), Function (TUG/mTUG), Falls (Calander/FallsMonitor@home), Strength (30 second chair stand), Fear of Falling (Short FES-I), Health related quality of life (EQ5D-5L/ ICE-CAP-O), resource use, adherence (my activity programme/EARS).<br>Baseline, 3, 6 months.

For peer review only

Sampling principles and procedures

Eligibility

Older adults at risk of falls (aged 50+ years) and assessed as requiring a falls rehabilitation exercise programme, are identified through current community falls rehabilitation services delivered in Manchester City and Trafford. Exclusions include older adults who are unable to follow instructions (unless supported by a family member/carer), who are unable to understand written English (unless supported by a family member/carer), with severe visual impairment, those in long-term residential or nursing care and those with terminal illness or expected shortened lifespan, defined as less than 6 months, as determined by the NHS teams. Patients need to have good 3G/4G mobile phone reception or wifi in their home and this is assessed by the health professional before they handout participant information.

Recruitment, consent, sample size

Health professionals give patients the study information sheet and inform them about the intervention. The health professionals then ask the patient if they are happy to be contacted by the researcher who demonstrates the technology either in the patient's home or within groups at each NHS site. The technology is demonstrated to the participant before they are asked to give informed consent. Where possible a former patient who has used the smartphone applications accompanies the researcher to demonstrate the technology. Involvement of a peer assists in promoting patient confidence in the use of the technology.

The first 36 eligible patients identified through each service (N=72 in total) who are willing to participate are being recruited and randomised. Thirty patients per arm after attrition (approx. 10%) are normally used for feasibility RCTs[22]. Study participants are randomised using a computer-generated randomisation algorithm at sealedenvelope.com, stratified by gender and site, using block randomisation (2, 4, 6 blocks) into either intervention or control group.

Blinding

Baseline and follow-up (3 and 6 months) assessments are carried out by experienced clinicians within each NHS Trust (not a member of the clinical teams participating), who are blinded to which intervention the participants are receiving, at baseline the individual is also blinded to the intervention they will receive as randomisation occurs after baseline assessment.

As this is an 'active' intervention, it is not possible to blind the health professionals delivering the service or the participants during the intervention. The lead researcher provides technical support to both arms to use the smartphone so is not blinded.
The statistical analysis will be carried out by the lead researcher with the support of a statistician. Patient ID codes will be removed from the data to allow for blinded analysis.

Patient withdrawal

In consenting to the trial, patients are consenting to the trial treatment, follow-up and data collection. If withdrawal of the randomly allocated treatment occurs, patients should still be followed up where they agree. Patients are allowed to withdraw without giving reason at any time and a withdrawal case report form (CRF) will be completed to document the date and reason (where given) for withdrawal. Data collected up to the time of withdrawal will be included in analyses. Health professionals will assess patients' capacity to take part in the rehabilitation programme and the study, if they have been deemed to have lost capacity to consent they will be withdrawn from the study but the data already collected will be retained.

Interviews with patients

All participants are offered an interview (even those who withdraw from the trial) in their own home after the final follow-up to assess their experiences of the intervention and trial processes. Family members/carers may also attend the interview at the participants' request.

Focus groups with health professionals

For peer review only - http://bmjopen.bmj.com/site/about/guidelines.xhtml

Health professionals from Trafford and Manchester city who are involved in the study are recruited to participate in a focus group at the end of the study (after 24 weeks follow-up). All members of staff (N=8) will be given study information by their team leader and asked if they are available for a focus group, the focus groups will take part at their place of work at a time convenient to each team. Participating staff can choose to be part of a one-to-one interview if they prefer not to be interviewed with colleagues or if for staffing reasons it is not feasible for them to attend the focus group.

The Intervention

Full details of the intervention components are shown in Supplementary material: Table 1 (TIDieR Guidelines).

The technology

The Samsung Galaxy J5 as a means of communication[23] will be provided to all participants and health professionals.

'Motivate me' app

The 'Motivate me' app is the health professional application. This app is used by the health professional with the patient to set behavioural and outcome-based goals, for the health professional to see what exercises the patient has reported and to give feedback and to check they have received messages.

'My activity programme'

'My activity programme' is the patients application. This app will be used by the patient to report the exercises they have done, receive messages and prompts and to confirm whether they like the messages received .

There are 12-behaviour change techniques adopted [24] through the intervention include goal-setting (behaviour/outcome), action-planning (recording plan to exercise in diary on smartphone/reminder text messages when it is time to start the programme), and feedback on behaviour (providing feedback on what they have done/benefits). The key behavioural change techniques delivered as part of the control and intervention arms are outlined in Table 2.

Table 2: Behaviour change techniques adopted*

	**1.Intervention arm**	**1a How**	**2.Control arm (standard service)**	**2a How**

For peer review only - http://bmjopen.bmj.com/site/about/guidelines.xhtml

1.1 Goal setting (behaviour)	x	what, when, where- smartphone and paper	x	What, Where- Paper
1.3 Goal setting (outcome)	x	smartphone verbally	x	Verbally
1.4 Action planning	x	smartphone		
1.5 Review Behavioural goals	x	smartphone verbally	x	Paper Verbally
1.7 Review outcome goals	x	smartphone verbally	x	Verbally
2.2 Feedback on behaviour	x	Smartphone verbally	x	Verbally
4.1 Instructions on how to perform the behaviour	x	Physically<br>Smartphone Paper	x	Physically<br>Paper
5.1 Information about health consequences	x	Smartphone Verbally (ad hoc)	x	Verbally (ad hoc)
5.6 Emotional Consequences	x	Smartphones Verbally (ad hoc)	x	Verbally (ad hoc)
6.1 Demonstration of behaviour	x	Physically	x	Physically
7.1 Prompts	x	Smartphone		
8.7 Graded tasks	x	Smartphone Paper	x	Paper

*Based on Michie et al[24] behaviour change taxonomy

The Control

Standard service is variable across different sites, but all sites deliver a mix of the evidence-based Falls Management Programme (FaME) and Otago [25] exercises as standard care.

For peer review only - http://bmjopen.bmj.com/site/about/guidelines.xhtml

They include face-to-face delivery once-a-week and a prescribed home-exercise programme (with booklet) with informal outcome-based goal-setting.

Manchester City: Once a week visits (either home-based or group exercise) for around 12 weeks (dependent on need) and then check-ups until 6 months discharge.
Trafford: 8 weeks group exercise once a week then discharged, or 6 week home-based exercise then discharged or referred to further 8 week group exercise.

Both sites leave participants with a home exercise plan on discharge and where appropriate refer onto community-based strength and balance programmes.

Control application for self-reporting exercise

The control arm receives a basic app where they report their exercises, but they are only able to report their exercises (outcome measure), they are not able to view their programme, receive messages or receive feedback on the phone. The health professional is not able to view what they have reported (outcome measure for the research team only).

Co-treatments
Trial participants are free to seek management of falls and other related or unrelated medical conditions during the course of the trial. We record all health service resource use and these will be reported as a trial outcome. At trial closure, participants will continue with usual healthcare, no further ancillary care is provided beyond that immediately required for the proper and safe conduct of the trial.

Intervention fidelity checks and process evaluation
We report intervention fidelity, process and compliance using observation during quality assurance visits and health professionals and the assessor will follow a Standard Operating Procedure (SOP) for assessment and intervention. The research team attend the goal-setting session with the health professional for the first 5 patients at each site to check fidelity and then attend alternate months (one patient). The trial treatment record (CRF) includes details of the grade and type of staff involved with delivery. The health professionals and researcher keep an issue-log of technical issues with the phones or apps during the trial either experienced directly or reported by participants.

Outcome measures

Data such as demographics (age, gender, socio-economics, health conditions, falls history) and physical tests are recorded on the CRF (Table 3).

Table 3: Schedule of enrolment interventions and assessments

	STUDY PERIOD					
	Enrolment	Allocation	Post-allocation			Post intervention
**TIMEPOINT****	$-t_1$	0	T_1	T_2	T_3	

For peer review only - http://bmjopen.bmj.com/site/about/guidelines.xhtml

**ENROLMENT:**						
**Eligibility screen**	X					
**Consent to further information**	X					
**Tech demo and Informed consent**	X					
**Allocation**		X				
**INTERVENTIONS:**						
Control: ***CFS*** ***TCS***			CFS ◆——— TCS ◆—◆	———	———◆	
Intervention			◆———	———	———◆	
**ASSESSMENTS:**						
**Gender** **Age** **Ethnicity** **Education** **Housing** **Falls history** **Medical history** **Technology use** **Allocated to home or group exercise**	X					
Falls (Calendar) ***Falls (alarm)*** ***My activity self-report*** ***Prescribed exercise plan*** ***Face to Face delivery***			◆———	———	———◆	
Berg ***TUG*** ***mTUG*** ***30 Second Chair Stand*** ***FES-1*** ***EQ5D*** ***Resource Use*** ***Health professional time resource*** ***ICE-CAP-O*** ***EARS***	X			X	X	
Interviews ***Focus groups***						X

For peer review only

Primary outcome measures

Assess feasibility and acceptability of the design and procedures including:

a. willingness of participants to be randomised (collected on the screening section of the CRF/interviews)
b. willingness of clinicians to recruit participants (through focus group/completion of screening on the CRF)
c. number of eligible patients (through the CRF and documentation of number of monthly referrals to falls services)
d. characteristics of the proposed outcome measures e.g. reliability of falls detector.
e. follow-up rates, adherence/compliance rates
f. time needed to collect and analyse data
g. determine effect sizes for use in sample-size calculations, enabling power calculations for the reduction in falls for a definitive large scale RCT.

Start/stop criteria for going to full trial are included in CONSORT diagram (Diagram 1) based on these outcome measures.

Secondary outcome measures

Falls
The primary outcome for any future definitive trial would be falls, expressed as fall rate per person per months of follow-up observation after randomisation.

All participants will wear the smartphone in their pocket or on a waistband and this will act as a falls detector, running the FallsMonitor@home app developed by the University of Bologna[26]. If the patient chooses then they can also use the app on the phone as a falls alarm. The fall detection system application allows the user to identify a list of formal/informal caregivers who will receive an SMS if a fall is detected. Patients are given an opportunity to de-active the falls alarm through an application on the smartphone if there is a false alarm, enabling the user to maintain control and prevent unwanted intrusion. Participants are asked if we can use their anonymised falls data for further development of the app and in the Farseeing real-world falls database[27].

To validate this as an outcome measure we use the internationally agreed ProFaNE falls definition[28] and follow the agreed ProFaNE falls data collection and analysis protocols based on self-report calendars[29].

Fear of falling
Short Falls Efficacy Scale-International (Short FES-1) is used to measure fear of falling[30]. This is often a measure used by UK falls services as part of standard outcome measures.

Function
The Timed Up and Go test (TUG) will be used to assess improvements in mobility and function. The TUG will be applied as described by Podsiadlo and Richardson[21]. Participants will be asked to perform the TUG at their self-selected habitual walking speed. A medical device implementing an instrumented version of the TUG will be used

For peer review only - http://bmjopen.bmj.com/site/about/guidelines.xhtml

(mTUG, mHealth Technologies s.r.l., Bologna, Italy). The device is able to automatically provide guidance to the user for administering the test, capture and process the data, and generates summary reports of function for the health professional. The blinded assessor will complete the normal TUG and the mTUG as outcome measures (the standard TUG as a validation measure) to explore whether the mTUG is usable as an outcome measurement for the definitive RCT. The health professional will carry out the mTUG with a sub-sample of 10 patients at each site to assess their experiences of its use.

Balance
The Berg Balance Scale will be used to assess balance. This has good validity and sensitivity in this population[31] and is one of the best outcome measures for assessing standing balance[32]. It has also been used for the prediction of falls[33]. The effect sizes from this outcome measure scale will be used as part of the power calculation for the full trial.

Strength
30 seconds chair stand test[34], which has good validity and is used throughout health services will be used to assess physical ability, in particular strength.

Adherence
Adherence will be measured in a number of ways (outlined in detail, Table 5):
1) Self-report app will be used for both control and intervention group. Adherence will be classed as the participant carrying out 80% of their prescribed programme (based on the evidence-base for effective strength and balance)[6,35].
2) Exercise Adherence Rating Scale (EARS)[36]. This is a validated 16-question tool with a 6-question subscale specifically measuring adherence (remaining questions measure reasons for adherence/non-adherence).

Health economics
The health economics analysis is focussed on informing relevant measures and means of collection of health related quality of life and resource use for the future definitive study. Only an exploratory cost-effectiveness analysis will be conducted, for all measures we will report mean values and sample variability alongside information on missing values. The health related quality of life measures will include the European Quality of Life 5 Dimensions (EQ-5D-5L)[37] and an additional measure used in previous trials related to falls prevention (the ICEpop CAPability measure for Older people (ICE-CAP-O)[38,39]. Costs of delivering the intervention will be observed based on staff training, delivery costs and equipment costs. Additional resource use measures will be captured via a Resource Use Questionnaire which will seek to measure costs related to an NHS and social care perspective (secondary, primary, community care service use), and a patient perspective (costs related to informal care). The findings from these will inform the feasibility of collection of the data, and priorities for cost collection at full trial.

For peer review only - http://bmjopen.bmj.com/site/about/guidelines.xhtml

Table 5: Adherence measures

	**What**	**How/additional validation**
**Self-report through my activity programme and control arm smartphone app**	Exercises reported on app to their prescribed programme they day they are carried out.<br>- Exercise Type<br>- Intensity<br>- Dose<br>Adherence defined as participant carrying out 80% of their prescribed programme.	The health professional will be asked to provide a copy of the participants prescribed exercise plan, any changes to it and the dates any changes were made (both sites record this as part of standard intervention).<br><br>For face to face home delivery, the health professional will be asked to report exactly what the patient has done when with them (this will be used to validate the self-report from participants).<br><br>After discharge from rehabilitation if participants move onto other strength and balance provision. Those services will give us copies of the exercise programme delivered for any days the participants attend, attendance records and any prescribed home exercise programme.
**EARS**	Validated 16-question tool with a 6-question subscale specifically measuring adherence.	Paper questionnaire at baseline, 3 months and 6 months.

For peer review only

Interviews/focus groups

The interview and focus group schedules are based on FARSEEING guidelines[40]. The following key areas will be explored in relation to the smartphone, the 'Motivate me' app, 'My activity programme' app, the falls alarm, and the mTUG. Ease of use, clarity of screen, demonstration of use, wearing comfort, adaption of use, reliability, choice and control and home and lifestyle. This feedback will be considered and any required changes to the technology set-up, applications and intervention will be made prior to the definitive RCT. We also ask additional questions about the research process including: general expectations and views; experiences of recruiting patients (health professional), and of being recruited and randomised (patients), suggestions of methods for recruiting participants; likely uptake and retention of participants.

Analysis

Quantitative data is analysed using SPSS Release 22.0. The main analyses is descriptive,

For peer review only - http://bmjopen.bmj.com/site/about/guidelines.xhtml

involving the estimation of recruitment rates, attrition rates, non-compliance rates, means and standard deviations of outcomes by group at baseline and end trial, and 95% confidence intervals for differences of means of outcomes between groups and assessment of change following the intervention at end trial. Primary and secondary outcomes at end trial will also be compared by group using intention-to-treat analysis. z and t test for continuous outcomes and chi-square test for categorical outcomes (or their regression equivalent) will be adopted to compare intervention effect. Adjustment for baseline characters or site effect will be considered whenever necessary. However, such inferential analyses will need to be interpreted with great caution as the study will not be powered to detect significant differences, as the main aim is to assess proof of concept, feasibility and inform a full-scale trial. A statistical analysis plan will be created before data analysis.

Qualitative interviews/focus groups will be analysed using thematic analysis[41]. The research will be inductive and although will seek to further understand the quantitative findings, this approach will also generate categories and explanations directly from the data rather than based on previously set aims and objectives, reducing risk of bias[41]. QSR International's NVivo 10 qualitative data analysis software will be used to manage the data. The validity of the analysis will be checked by returning to the data once themes have been identified and also through the use of a second researcher who will check samples of analysis. The accuracy of the transcripts will be checked through discussion with participants to establish if anything is not clear from the interviews/focus groups.

Ethical issues

Ethical approval has been granted from the North West Greater Manchester East Research Ethics Committee (Rec ref: 18/NW/0457, 9/07/2018). Regional and site-specific approvals have been obtained. As this is a study with older patients a number of ethical issues could arise. To address these, community services will act as gatekeepers to access patients and assess patients' eligibility for the study. The intervention is delivered by health service staff and provided in addition to standard service, therefore patients are unlikely to be disadvantaged.

If falls are detected by the smartphone, it is important that someone is informed in real-time. The smartphone application allows the user to select a list of formal/informal caregivers who will receive an SMS if a fall is detected. It will be made clear that the falls service is not an emergency service so in the event of a fall the person receiving the text message would call an ambulance as they would in normal circumstances. If patients already wear a call alarm then they will be encouraged to continue to use this as well or to adopt their usual method of alerting help.

The study requires monitoring of subjects and it is important that patients do not find this obtrusive (privacy issues have been identified as major barriers to the use of technology). Patients are given an opportunity to de-active the falls alarm through an application on the smartphone if there is a false alarm. However, previous consultation/usability testing with older adults raised no major privacy issues.

There are ethical issues in the removal of technology at the end of studies[42]. We will not be able to offer older adults the technology at the end of the 6 month study period, but they

For peer review only - http://bmjopen.bmj.com/site/about/guidelines.xhtml

will be offered the opportunity to download the apps onto their own phones if they wish.

The risk of interviews and focus groups are minimal. The patient or health professional can ask the researcher to move onto another question if they are uncomfortable at any point. Health professionals will be given the chance to discuss the trial, technology and intervention in a one-to-one interview if they do not feel comfortable giving feedback in front of colleagues.

Patient and public representatives have been involved in designing the trial, study material and assisting with recruitment.

Trial monitoring

The lead researcher (HHH) will monitor the delivery of the intervention and recruitment of patients, there will also be a clinical lead (AE, EM) at each site taking overall responsibility for identification of patients and delivery of the intervention. This team, alongside academic experts (JH, LC, SM, ASM, CT) form the Trial Co-ordination Group who ensure overall quality of trial data. There is an advisory group (AG), which meets bi-annually, giving feedback on the project, providing expert guidance and assisting in dissemination, this includes two previous patients. A risk register is reviewed by the AG. The study is subject to the audit and monitoring regime of the University of Manchester and a monitoring plan followed.

A detailed risk assessment has been carried out and potential patient, organisational and study hazards considered, the likelihood of their occurrence and the resulting impact should they occur.

Adverse events

A safety reporting protocol has been developed for related and unexpected serious adverse events (AEs) and directly attributable AEs. An AE is defined as any untoward medical occurrence in a subject which does not necessarily have a causal relationship with treatment. These AEs are recorded in the CRF and if a Serious Adverse Event (SAE) occurs then reported to the Chief investigator. The CI will determine whether AEs require reporting to the trial sponsor and Ethics Committee, in accordance with the safety reporting protocol.

DISCUSSION

This is the first trial that we are aware of that explores the potential use of motivational smartphone apps for the support of an evidence-based falls exercise programme. As this is an active intervention and control we are unable to blind participants or those delivering the intervention. However, the design does enable us to blind both those carrying out the assessments and analysis.

This trial assesses several novel outcome measures against the gold standard, the mTUG against standard TUG, the FallsMonitor@home against standard calendar method and a self-report app against the EARS tool[36]. This enables us to further our understanding of whether technology has the potential to provide more objective and reliable outcome

For peer review only - http://bmjopen.bmj.com/site/about/guidelines.xhtml

measures than current methods.

We use two very different NHS sites, reflecting the reality of day-to-day practice (one specialist falls service, one general rehabilitation services) to explore the delivery of the intervention. This means that the standard service is different across the two sites adding complexity to how the control and intervention arm are delivered. However, these differences enable us to assess its scalability to full trial where different types of falls services would need to be included as sites. It also enables us to be more representative of current services and assess its potential for delivery in practice.

Authors' contributions:

HHH leads the research project and its design, managing the trial overall and has led the writing of the protocol. CT and SB give technical support for the study and have advised on outcomes and the manuscript. JH, LC, CT and SM have provided scientific advice around the design of the study and commented on the manuscript. ST and YF have given advice on statistics and health economic part of design and manuscript. AE and EM have given advice on the operationalisation of the study and commented on the manuscript.

Acknowledgments

Thanks to Professor Dawn Skelton and Later Life Training for allowing us to use their images and the name 'Motivate Me' for the health professional app.

Competing Interests

No competing interests declared.

Funding
This study is independent research arising from a NIHR Postdoctoral Fellowship Award, Dr Helen Hawley-Hague, (PDF -2015-08-012) supported by the National Institute for Health Research**.** The views expressed in this publication are those of the author(s) and not necessarily those of the NHS, the National Institute for Health Research or the Department of Health.

For peer review only - http://bmjopen.bmj.com/site/about/guidelines.xhtml

REFERENCES

1. Public Health England. Muscle and bone strengthening and balance activities for general health benefits in adults and older adults. Summary of a rapid evidence review for the UK Chief Medical Officers' update of the physical activity guidelines. London 2018. https://assets.publishing.service.gov.uk/government/uploads/system/uploads/attachment_data/file/721874/MBSBA_evidence_review.pdf (accessed 15 November 2018)
2. Yardley, L., Kirby, S., Ben-Shlomo, Y, et al. How likely are older people to take up different falls prevention activities? *Prev Med*2008;47:554-58. doi: 10.1016/j.ypmed.2008.09.001
3. Gillespie, L.D., Robertson, M.C., Gillespie, W.J, et al. Interventions for preventing falls in older people living in the community. *Cochrane Database of Systematic Reviews.* 9, 2012 Art. No.: CD007146. doi: 10.1002/14651858.CD007146.pub3.
4. Sherrington, C., Tiedemann, A., Fairhall N, et al. Exercise to prevent falls in older adults: an updated systematic review and meta-analysis. *Br J Sports Med*2018;51:1750-1758. doi: 10.1136/bjsports-2016-096547
5. Public Health England (2018) An ROI Tool: Assessment of Falls Prevention Programmes for Older People Living in the Community. Crown Copyright. https://assets.publishing.service.gov.uk/government/uploads/system/uploads/attachment_data/file/679856/A_return_on_investment_tool_for_falls_prevention_programmes.pdf (accessed 6 August 2018)
6. Nyman, S.R. Victor, C.R. Older people's participation in and engagement with falls prevention interventions in community settings: an augment to the Cochrane systematic review. *Age Ageing*2011;41:16-23. doi: 10.1093/ageing/afr103.
7. Royal College of Physicians. Older people's experiences of therapeutic exercise as part of a falls prevention service-patient and public involvement. London 2012: RCP.
8. Rapp, K., Büchele,G., Dreinhöfer, k, et al. Epidemiology of hip fractures. *Z Gerontol Geriatr*, 2018: 1-7. https://doi.org/10.1007/s00391-018-1382-z.
9. Hamm, J., Money, AG., Atwal, A, et al. Fall prevention intervention technologies: a conceptual framework and survey of the state of the art *J. Biomed. Inform*2016;59:319-345. https://doi.org/10.1016/j.jbi.2015.12.013
10. CONFIDENCE (2011) D8.5: Final report. http://www.confidence-eu.org/ (accessed 8 August 2014)
11. Fukuoka, Y., Kamitani, E., Bonnet, K, et al. Real-Time Social Support Through a Mobile Virtual Community to Improve Healthy Behavior in Overweight and Sedentary Adults: A Focus Group Analysis. *J Med Internet Res*2011;13:e49.
12. Vankipuram, M., McMahon., S., Fleury, J. ReadySteady: App for Accelerometer-based Activity Monitoring and Wellness-Motivation Feedback System for Older Adults. *AMIA Annual Symposium Proceedings*2012:931–939.
13. Worringham C, Rojek A, Stewart I, et al.Development and Feasibility of a Smartphone. ECG and GPS Based System for Remotely Monitoring Exercise in Cardiac Rehabilitation. *PloS one*2011:6. doi: e14669-10.1371/journal.pone.0014669
14. Rabin, C. Bock, B. Desired features of smartphone applications promoting physical activity. *Telemed J E Health*2011;17:1-3. doi: 10.1089/tmj.2011.0055
15. King, A.C., Hekler, E.B., Grieco, L.A, et al. Harnessing Different Motivational Frames via Mobile Phones to Promote Daily Physical Activity and Reduce Sedentary Behavior in Aging Adults. *PlosOne*2013;8. DOI: 10.1371/journal.pone.0062613
16. Epton, T., Currie, S., Armitage, C. J. Unique effects of setting goals on behavior change: Systematic review and meta-analysis. *J Consult Clin Psychol*2017;85:1182-98.

For peer review only - http://bmjopen.bmj.com/site/about/guidelines.xhtml

doi: 10.1037/ccp0000260
17. Hawley-Hague, H., Horne, M., Campbell, M, et al. Multiple levels of influence on older adults' attendance and adherence to community exercise classes. *Gerontologist*2014; 54:599-610. doi: 10.1093/geront/gnt075
18. Yardley, L., Donovan-Hall, M., Francis, C, et al. Attitudes and beliefs that predict older people's intention to undertake strength and balance training. *J Gerontol B Psychol Sci Soc Sci 2007*;2:199-225. doi: 10.1093/geronb/62.2.P119
19. Ajzen, I. *Attitudes, Personality and Behaviour*. Maidenhead, UK: Open University Press 1988.
20. Lucidi, F., Grano, C., Barbaranelli, C, et al. Social-cognitive determinants of physical activity attendance in older adults. *J Aging Phys Act2006;*14:344-59.
21. Podsiadlo, D., Richardson, S. The timed "Up & Go": a test of basic functional mobility for frail elderly persons. *J Am Geriatr Soc*1991;39:142–48.
22. Lancaster, GA. Dodd, S., Williamson, P.R. Design and analysis of pilot studies: recommendations for good practice. *J Eval Clin Pract* 2004; 10:307-12. doi:10.1111/j.2002.384.doc.x
23. Boulton, E. Hawley-Hague, H., Vereijken, B, et al. Developing the FARSEEING Taxonomy of Technologies: Classification and Description of Technology Use (including ICT) in Falls Prevention Studies. *J Biomed Inform* 2016; 61:132-140. doi: 10.1016/j.jbi.2016.03.017
24. Michie, S., Richardson, M., Johnston, M, et al. The behavior change technique taxonomy (v1) of 93 hierarchically clustered techniques: building an international consensus for the reporting of behavior change interventions. *Ann Behav Med*2013;46:81-95. doi: 10.1007/s12160-013-9486-6
25. ProFouND. Strength and Balance Home Exercise Booklet for Older People (English) 2013. http://profound.eu.com/strength-and-balance-home-exercise-booklet-for-older-people-english/ (accessed 8 November 2018)
http://profound.eu.com/otago-exercise-program/#
26. Mellone S, Tacconi C, Schwickert L. Smartphone-based solutions for fall detection and prevention: the FARSEEING approach. Z Gerontol Geriatr2012;45:722-7. doi: 10.1007/s00391-012-0404-5.
27. FARSEEING. The FARSEEING real world falls repository, http://farseeingresearch.eu/the-farseeing-real-world-fall-repository-a-large-scale-collaborative-database-to-collect-and-share-sensor-signals-from-real-world-falls/ (accessed 5 August 2018)
28. Lamb S, Jørstad-Stein, EC, Hauer K, et al. Development of a common outcome data set for fall injury prevention trials: the Prevention of Falls Network. Europe consensus *JAGS*,2005;53:1618–1622 doi:10.1111/j.1532-5415.2005.53455
29. Hauer K, Lamb SE, Jorstad EC, et al. Systematic review of definitions and methods of measuring in randomized controlled fall prevention trials. *Age Ageing,*2006;35:5-10. doi: 10.1093/ageing/afi218
30. Kempen G, Yardley, L Van Haastregt, J.C.M, et al. The Short FES-I: a shortened version of the Falls Efficacy Scale-International to assess fear of falling. *Age Ageing*.2008;37:45-50 doi: https://doi.org/10.1093/ageing/afm157
31. Berg K,O., Wood-Dauphinée, SL., Williams, JI, et al. Measuring balance in the elderly: validation of an instrument. *Can J Public Health*1992; S2:S7–S11.
32. Sibley KM, Howe T, Lamb SE, et al. Recommendations for a Core Outcome Set for Measuring Standing Balance in Adult Populations: A Consensus-Based Approach. *Plos1*2015. https://doi.org/10.1371/journal.pone.0120568

For peer review only - http://bmjopen.bmj.com/site/about/guidelines.xhtml

33. Lajoie, Y., Gallagher, S.P. Predicting falls within the elderly community: comparison of postural sway, reaction time, the Berg Balance Scale and the Activities-specific Balance Confidence (ABC) scale for comparing fallers and non-fallers. *Arch Gerontol Geriatr*2004;38:11-26
34. Jones CJ, Rikli RE, Beam WC. A 30-s chair-stand test as a measure of lower body strength in community-residing older adults. *Res Q Exerc Sport*1999;70:113–19. doi: 10.1080/02701367.1999.10608028.
35. Osho O, Owoeye O, Armijo-Olivo S. Adherence and Attrition in Fall Prevention Exercise Programs for Community-Dwelling Older Adults: A Systematic Review and Meta-Analysis. *J Aging Phys Act* 2017. 3:1-34. doi: 10.1123/japa.2016-0326
36. Newman-Beinart, N., Norton, S., Dowling, D, et al. The development and initial psychometric evaluation of a measure assessing adherence to prescribed exercise: the Exercise Adherence Rating Scale (EARS). *Physiotherapy*2017;103:180-85. doi: 10.1016/j.physio.2016.11.001
37. Janssen MF, Pickard AS, Golicki D, et al. Measurement properties of the EQ-5D-5L compared to the EQ-5D-3L across eight patient groups: a multi-country study. *Qual Life Res* 2013;22:1717-1727. doi: 10.1007/s11136-012-0322-4
38. Grewal I, Lewis J, Flynn T, et al. Developing attributes for a generic quality of life measure for older people: Preferences or capabilities? *Soc Sci Med*. 2006;62:1891-1901
39. Davis J, Liu-Ambrose T, Richardson C, et al. A comparison of the ICECAP-O with EQ-5D in falls prevention clinical setting: are they complements or substitutes? *Qual life Res*2012;22:969-77. doi: 10.1007/s11136-012-0225-4
40. FARSEEING**.** Preliminary guidelines for design and implementation of technologies 2014. http://farseeingresearch.eu/wp-content/uploads/2014/07/Preliminary-Guidelines-V1.0-Public.pdf (accessed 8 August 2017)
41.Braun V. Clarke V. Using thematic analysis in psychology. *Qualitative Research in Psychology* 2006;3:77-101. doi.org/10.1191/1478088706qp063oa
42. Magnusson, L., Hanson, E.J. Ethical issues arising from a research, technology and development project to support frail older people and their family carers at home. *Health Soc Care Community* 2003;11:431-9.

.

.

For peer review only - http://bmjopen.bmj.com/site/about/guidelines.xhtml

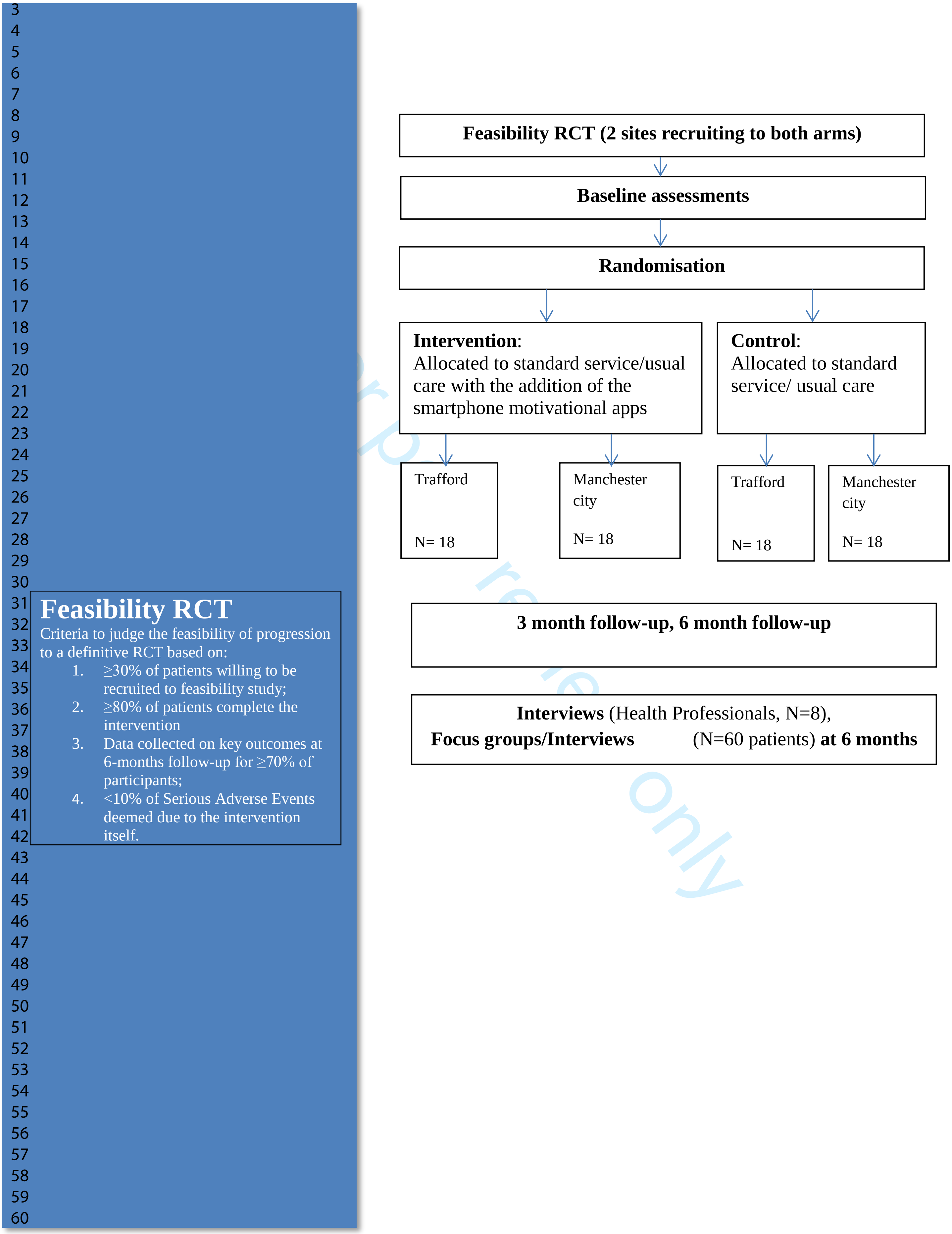

For peer review only

Supplementary Material Table 1: Intervention description using the Template for Intervention Description and Replication (TIDieR) checklist*

**Item 1: Name**	Can smartphone **T**echnol**OG**y be used to support an **E**ffec**T**ive **H**ome **E**xe**R**cise intervention to prevent falls amongst community dwelling older people: The TOGETHER trial	
**Item 2: Why**	Strength and balance training has been found to be effective in reducing the rate and risk of falls. Health services are often unable to deliver the evidence-based dose of exercise and older adults do not always sufficiently adhere to their programme to gain full outcomes. This feasibility trial will explore whether smartphone technology based on the Theory of Planned Behaviour and using goal setting, feedback and prompts can be used to support patients to better adhere to an evidence-based exercise rehabilitation programme and test study procedures and outcome measures.	
**Item 3: What (Materials)**	**Intervention arm:** Behaviour change apps	**Control arm:**<br><br>Standard service
	Samsung Galaxy J5<br><br>**Health Professional phone based Motivate Me app**: -<br>1. set patients long-term goals (outcomes)<br>2. set patients behavioural goals (which evidence based exercises they will do and how often, when they will exercise with the health professional, alone or in a group),<br>3. access the patients self-report data and see what exercises they have been doing and when.<br>4. upgrade exercise programme.<br>5. give the patient bespoke feedback (set as once a week).<br><br>**Patient phone based My Activity Programme app:-**<br>1. report the exercises they have done (exercise type, duration, intensity)<br>2. receive prompts when they have scheduled to exercise<br>3. receive automated motivational messages based on the long-term goals they have set (with a focus on strengthening outcome	Samsung Galaxy J5<br><br>**Control group self-report app:-** report the exercises they have done (exercise type, duration, intensity)<br><br>Home exercise booklet<br><br>Calander/FallsMonitor@home<br><br>Patient ‘How to guide’<br><br>Technology issue log (Health professional)

For peer review only

	expectations)<br>4. Receive bespoke feedback from health professionals (aimed at increasing Perceived Behavioural Control).<br><br>Home exercise booklet<br><br>Calander/FallsMonitor@home<br><br>Patient 'How to guide'<br>Health professional 'How to guide' (for technology)<br><br>Technology issue log (Health professional)	
**4. What procedure**	Screening and assessment as part of standard service by Falls Services<br><br>Support and training on how to use the 'My Activity Programme app' and falls alarm.<br><br>Formal goal setting session with health professional where use their 'Motivate me' app to set patients' behavioural and outcome-based goals (including prescribing the exercise programme). Handing out of home exercise booklet.<br><br>Strength and balance rehabilitation in group or home commences.<br><br>**Trafford:**<br>If recruited through Community Rehabilitation team they receive a 6- week programme. They are then either referred to group-based rehabilitation or discharged. Seen in own home once a week.<br><br>If recruited through group-based rehabilitation they receive an 8 week group-based programme. Seen in a group once a week.<br><br>They are then referred on to a community based FaME class once a week and prescribed home	Screening and assessment as part of standard service by Falls Services.<br><br>Some discussion around long term goals and home exercise booklet given and programme prescribed (behavioural goals).<br><br>Strength and balance rehabilitation in group or home commences.<br><br>**Trafford:**<br>If recruited through Community Rehabilitation team they receive a 6 week programme. They are then either referred to group-based rehabilitation or discharged. Seen in own home once a week.<br><br>If recruited through the group-based rehabilitation they receive an 8 week group-based programme. Seen in a group once a week.<br><br>They are then referred on to a community based FaME class once a week and prescribed home exercise programme.<br><br>**Manchester City**<br>They receive either a 12 week group based exercise class and prescribed home exercise programme or just a 12 week home based exercise, and

For peer review only

	exercise programme.<br><br>**Manchester City**<br>They receive either a 12 week group based exercise class and prescribed home exercise programme or just a 12 week home based exercise, and then follow-up visits until 6 months (as per patient need). On discharge they are referred to the exercise referral programme where they can attend a group once a week and receive a prescribed home exercise programme. Sometimes they are referred to exercise referral programme before discharge.<br><br>All patients across both sites will receive a prescribed programme by the health professional on discharge and the health professional will set behavioural goals with the patient using the apps. Throughout the rehabilitation period the health professional can upgrade the exercise programme as appropriate using the apps. They will also send the patient a personalised motivational message once a week until discharge.<br><br>Participants receive a visit from the research team the week after their rehabilitation programme has started and then monthly phonecalls.<br><br>Participants can contact the research team for technical support at any time.	then follow-up visits until 6 months (as per patient need). On discharge they are referred to the exercise referral programme where they can attend a group once a week and receive a prescribed home exercise programme. Sometimes they are referred to the exercise referral programme before discharge.<br><br>All patients across both sites will receive a prescribed programme by the health professional on discharge.<br><br>Participants receive a visit from the research team the week after their rehabilitation programme has started and then monthly phonecalls.<br><br>Participants can contact the research team for technical support at any time.
**Item 5: who provided**	**Assessments**<br>All physical assessments completed at the clinical sites are completed by blinded clinical staff from those sites. All assessors are qualified physiotherapists.<br>Assessments are completed at baseline (prior to randomisation), 3 and 6 months. Other assessments are self- completed by the older person.<br><br>**Intervention**<br>Both the intervention and control are delivered by the same health	

For peer review only

	professionals at both sites. These are predominantly Physiotherapists, but also Occupational Therapists and Healthcare Assistants. All health professionals have been trained to deliver the evidence based rehabilitation programme and have also received training on using the smartphone from the research team.		
**6. How**	**Identification**	Patients are identified by health professionals at 2 sites and screened against the inclusion/exclusion criteria. They are then approached by the health professional and asked if they would like a demo of the technology.	
	**Consent**	The researcher contacts the individual and arranges a visit accompanied by a former patient who has used the smartphone app before.<br>The researcher takes consent.	
	**Exercise delivery**	Exercise delivery is the same across intervention and control, but differs dependent on site (see 4.What). Exercise is delivered by the health professional either in the home or within a group.	
	**Motivational input**	**INTERVENTION**	**CONTROL**
		Patient carry out goal setting session with health professional structured by the use of the 'Motivate Me' app and receive a prescribed programme on their app and an exercise booklet.<br><br>Patient will receive prompts and messages through the 'my activity programme app' at home on the days they have planned to exercise.<br><br>Patients will also receive verbal feedback when they see the health professional.	Patient given a prescribed exercise programme and some informal goal setting is carried out as part of the session. They receive a home exercise booklet.<br><br>Patients will only receive verbal feedback when they see the health professional
**7. Where**	The intervention is delivered across several community venues and health centres venues and in patients' homes in Manchester City and Trafford.		
**8. When and how much**		**Intervention arm**	**Control arm**
	Research Team	1 visit to set- up the phone after randomisation	1 visit to set- up the phone after randomisation

For peer review only

		1 visit the week after the rehabilitation programme has commenced<br><br>Phone calls x 6	1 visit the week after the rehabilitation programme has commenced<br><br>Phone calls x 6
	Health Professional	2 x structured goal setting session (baseline and at discharge).<br><br>Weekly face to face exercise intervention (ranging from 6-15 contacts).<br><br>Weekly feedback message received through app until discharge (maximum of 26).	2 x goal setting session (baseline and at discharge).<br><br>Weekly face to face exercise intervention (ranging from 6-15 contacts).
	Smartphone	Automated messages and prompts x 3 on the day they have scheduled to exercise.	
**9. Tailoring**	Rehabilitation programme	The number of home based visits to each patient may differ across both recruitment sites and across both control and intervention groups dependent on patient need.<br><br>The exercise programme delivered will be tailored to each individual patient across both sites and across both control and intervention groups.<br><br>Both sites will send the weekly feedback message until patient discharge (this could be at 8 weeks at Trafford and 6 months at Manchester city).	
	Motivational messages	Because the exercise programme is tailored to the individual, health professionals may schedule for the patient to exercise 3 times a week on the phone or every day, dependent on preference. Participants will receive messages on the days they have scheduled to exercise some patients may receive more than others.	
**11. How well planned**	Health professional fidelity	The research team will attend the goal-setting sessions for the intervention arm for the first 5 patients at each site and then 1 patient at each site every 2 months.<br><br>All staff have undergone a half-day training session and a follow-up support session in using the smartphone app and trial procedures.	

	Assessment fidelity	Assessors masked to group allocation will follow an assessment standard operating procedure.
	Adherence	1. Adherence is collected through the smartphone apps (control and intervention).<br>2. At baseline, 3 and 6 months though validated questionnaire (EARS).<br>3. Through group exercise attendance records, health professional and instructor delivery records.

Item 10 and 12 N/A.

For peer review only

BMJ Open

Can smartphone TechnolOGy be used to support an EffecTive Home ExeRcise intervention to prevent falls amongst community dwelling older adults? The TOGETHER feasibility RCT study protocol.

Journal:	*BMJ Open*
Manuscript ID	bmjopen-2018-028100.R1
Article Type:	Protocol
Date Submitted by the Author:	18-Jun-2019
Complete List of Authors:	Hawley-Hague, Helen; University of Manchester, School of Health Sciences<br>Tacconi, Carlo; Universita degli Studi di Bologna Dipartimento di Ingegneria dell'Energia Elettrica e dell'Informazione Guglielmo Marconi<br>Mellone, Sabato; University of Bologna, Department of Electrical, Electronic and Information Engineering «Guglielmo Marconi»<br>Martinez, Ellen; Central Manchester University Hospitals NHS Foundation Trust<br>Easdon, Angela; Pennine Care NHS Foundation Trust<br>Yang, Fan; University of York, Centre for Health Economics<br>Su, Ting-Li; University of Manchester, Dentistry<br>Mikolaizak, A. Stefanie; Robert Bosch Krankenhaus<br>Chiari, Lorenzo; University of Bologna, Department of Electrical, Electronic and Information Engineering «Guglielmo Marconi»<br>Helbostad, Jorunn; The Norwegian University of Science and Technology, NTNU, Department of Neuromedicine and Movement Science, The Faculty of Medicine and Health Sciences<br>Todd, Chris; University of Manchester, School of Health Sciences
**Primary Subject Heading**:	Rehabilitation medicine
Secondary Subject Heading:	Geriatric medicine, Health services research
Keywords:	REHABILITATION MEDICINE, older, mhealth

SCHOLARONE™ Manuscripts

Can smartphone **T**echnol**OG**y be used to support an **E**ffec**T**ive **H**ome **E**xe**R**cise intervention to prevent falls amongst community dwelling older adults? The **TOGETHER** feasibility RCT study protocol.

Hawley-Hague, H[1]., Tacconi, C[2,3]., Mellone, S[2,3,4]., Martinez, E[5], Easdon, A[6]., Yang, F[7]., Su, T[8]., Mikolaizak, A.S[9]., Chiari, L[2,3,4]., Helbostad, J[10]., Todd[1, 11], C.

[1] School of Health Sciences, Faculty of Biology, Medicine and Health, The University of Manchester and Manchester Academic Health Sciences Centre, Manchester, U.K.
[2] Health Sciences and Technologies-Interdepartmental Center for Industrial Research, University of Bologna, Bologna, Italy.
[3] mHealth Technologies s.r.l., Bologna, Italy,
[4] Department of Electrical, Electronic and Information Engineering «Guglielmo Marconi» - University of Bologna, Bologna, Italy.
[5] Manchester University NHS Foundation Trust, Manchester, U.K
[6] Pennine Care Hospital Trust, Manchester, U.K
[7] Centre for Health Economics, University of York, York, U.K
[8] School of Medical Sciences, Faculty of Biology, Medicine and Health, The University of Manchester, Manchester, U.K
[9] Robert Bosch Krankenhaus, Department of Clinical Gerontology, Stuttgart, Germany
[10] Department of Neuromedicine and Movement Science, The Faculty of Medicine and Health Sciences, The Norwegian University of Science and Technology, NTNU, Trondheim, Norway
[11] Manchester University NHS Foundation Trust, Manchester, U.K.

Corresponding author:
Dr Helen Hawley-Hague
The University of Manchester
School of Health Sciences (Nursing, Midwifery and Social Work)
Floor 6, Jean McFarlane Building
Oxford Road
Manchester
M13 9PL
Helen.Hawley-Hague@manchester.ac.uk

Word count: 4,649

For peer review only - http://bmjopen.bmj.com/site/about/guidelines.xhtml

ABSTRACT

Introduction

Falls have major implications for quality of life, independence and cost to the health service. Strength and balance training has been found to be effective in reducing the rate/risk of falls, as long as there is adequate fidelity to the evidence-based programme. Health services are often unable to deliver the evidence-based dose of exercise and older adults do not always sufficiently adhere to their programme to gain full outcomes. Smartphone technology based on behaviour-change theory has been used to support healthy lifestyles, but not falls prevention exercise. This feasibility trial will explore whether smartphone technology can support patients to better adhere to an evidence-based rehabilitation programme and test study procedures/outcome measures.

Methods and analysis

A two-arm, pragmatic feasibility randomised controlled trial will be conducted with health services in Manchester, UK. Seventy-two patients aged 50+ years eligible for a falls rehabilitation exercise programme from two community services will receive: (1) standard service with a smartphone for outcome measurement only or (2) standard service plus a smartphone including the motivational smartphone app. The primary outcome is feasibility of the intervention, study design and procedures. The secondary outcome is to compare standard outcome measures for falls, function and adherence to instrumented versions collected using smartphone. Outcome measures collected include balance, function, falls, strength, fear of falling, health related quality of life, resource use and adherence. Outcomes are measured at baseline, three and six month post-randomisation. Interviews/focus groups with health professionals and participants further explore feasibility of the technology and trial procedures. Primarily analyses will be descriptive.

Ethics and dissemination.

The study protocol is approved by North West Greater Manchester East Research Ethics Committee (Rec ref:18/NW/0457, 9/07/2018). User groups and patient representatives were consulted to inform trial design, and are involved in study recruitment. Results will be reported at conferences and in peer-reviewed publications. A dissemination event will be held in Manchester to present the results of the trial. The protocol adheres to the recommended SPIRIT Checklist.

Trial registration number: ISRCTN12830220

Key words: Rehabilitation, older, technology

Strengths and limitations of this study

For peer review only - http://bmjopen.bmj.com/site/about/guidelines.xhtml

- The first study to examine a motivational app to support falls rehabilitation.
- Pragmatic feasibility trial enables us to establish whether it is feasible to use the motivational apps in practice.
- Multi-site study with different types of falls services, representative of UK service delivery.
-
- Due to the nature of the intervention we will not be able to blind participants or those delivering the intervention.

For peer review only

For peer review only - http://bmjopen.bmj.com/site/about/guidelines.xhtml

Falls are an important public health issue, with over 30% of people aged 65 and over falling at least once a year[1]. This has implications for quality of life, independence and cost to the health service[1]. Strength and balance training (SBT) comprises ‘carrying out exercises that increase muscle strength in the legs and improve balance’[2]. Strength and balance exercise programmes are effective in reducing risk and rate of falls and injuries[3]. Sherrington et al[4] have shown that for strength and balance programmes to be effective they need to be progressive, tailored and of adequate dose (3x a week for 50 hours, and then maintained). Work carried out by Public Health England[5] illustrates that to see a return on investment; fidelity to the evidence-base has to be carried-out (adequate dose, progression).

However, Nyman and Victor[6] report that adherence to evidence-based strength and balance programmes is poor. The National Health Service (NHS) only delivers programmes that are pre-dominantly 3 months or less[7], older adults do not carry out their exercise programme three times a week as prescribed (dose) or carry out the programme for a sufficient length of time to achieve and maintain the benefits[6,7]. Cost and appropriate staffing are cited as primary reasons for short NHS delivery[7].

Unless there are innovative new solutions to support the delivery of falls prevention exercises sufficiently to reduce falls risk and to prevent re-referral to services, over the coming decade it is estimated that population changes will result in service demand beyond the reach of current interventions[8].The use of smartphones to support falls rehabilitation could be one of the solutions. The proportion of older adults using smartphones is growing rapidly, with 39% of those aged 65 to 74 and 15% of those aged over 75 using smartphones[9]. Smartphones offer multiple opportunities to support healthy ageing and falls prevention as they are portable, can be body-worn and can therefore be used for falls detection, movement detection and motivation[10, 11, 12] The evidence which looks at the role of the smartphone for falls prevention is sparse[13], particularly for interventions focused on rehabilitation/strength and balance training. Although, there is a lack of specific evidence related to falls prevention interventions, there is evidence that older adults find mobile phones more usable than using a new device e.g. a falls alarm[14]. It has also been suggested that barriers to smartphone use in this population can be overcome through adequate support and affordability[15]. There is evidence supporting the use of mobile phone-based healthy lifestyle programmes[16,17], including to increase physical activity[17,18,19]. King et al[11], developed and tested smartphone applications (apps) based on behaviour change theory designed to motivate adults aged 45+ years. One of these included personalised goal-setting and behavioural feedback, successful evidence-based behavioural change techniques[20]. The apps recieved positive feedback from participants and increased physical activity.

We know from previous studies that attitudes and beliefs are important to the uptake of and adherence to exercise by older adults[21,22]. The Theory of Planned Behaviour (TPB) [23] is particularly useful for assessing older adults’ attitudes in relation to exercise uptake and adherence[21,22,24]. The TPB is based on three core components:
(i) perceived behavioural control (PBC), the perceived ease or difficulty of performing the behaviour.
(ii) social influences including, subjective norms (beliefs of important people e.g. family), perceived social support (support from others for behaviour) and modelling (following observed behaviour of others).

For peer review only - http://bmjopen.bmj.com/site/about/guidelines.xhtml

(iii) attitudes (outcome expectations)[23]. Focused on the advantages and disadvantages of the behaviour (outcome expectations) and when related to adherence, whether these advantages have occurred.

Attitudes measured by using a TPB-based tool have been significantly associated with exercise behaviour in a previous study[21]. This theory has informed the intervention overall and content of the motivational messages within the proposed intervention (focused on outcome expectations/PBC).

Smartphone technology-based motivational applications underpinned by behaviour change theory and developed with health professionals and older adults could be an effective way of encouraging maintenance of exercise and of successfully supporting adherence to evidence-based strength and balance training. We have already carried out usability and acceptability testing of the technology and two motivational apps (one for health professionals and one for patients), before planning this trial. The smartphone apps have been developed through several cycles of user-led design. Initially we carried out engagement workshops with older adults (AgeUK) and health professionals from one falls service in Manchester, followed by usability/acceptability testing with another falls service in Manchester and their patients (IRAS:205980). The use of this approach has enabled us to develop the apps, establish whether the technology is acceptable to older adults and health professionals (qualitative methods) and to check its usability (technology testing). Overall, the apps were acceptable to both patients and health professionals with the majority of suggested changes made to the health professionals' app to ensure it fit more easily with their practice. Changes following this testing included; improvements in the delivery of messages and a more streamline approach to scheduling activities for the health professional. Another suggested change was to make smartphone pens available to participants to aid in the use of the touchscreen.

This study now aims to explore whether it is feasible for smartphone technology to be used to support patients to sufficiently adhere to an evidence-based exercise rehabilitation programme. As a secondary aim it will assess whether technology-based outcome measures (smartphone-based falls alarm and Timed up and Go Test)[25] are reliable when compared to standard methods (e.g. falls calendars). Through a feasibility RCT we will explore the feasibility of using smartphone technology to support falls rehabilitation and test study procedures (e.g. suitability of outcome measures, standard deviation of the outcome measure, recruitment, randomisation, follow-up rates, retention, time required for analysis). Both arms of the trial will receive rehabilitation exercises and will report their exercises on a study provided smartphone but only the intervention arm will carry out goal-setting and receive feedback through the phone.

The intervention has the potential to:
1. Increase the amount of support the patient receives to adhere to their exercise, leading to increased adherence.
2. Increase exercise progression/dose which could be cost neutral/saving.
3. Enable health professionals to monitor compliance to the prescribed programme.
This could assist maintenance of health, reducing long-term falls risk and re-access to services.

For peer review only - http://bmjopen.bmj.com/site/about/guidelines.xhtml

METHODS

Trial design

Core trial information is presented in Table 1. This study is a two-arm pragmatic feasibility randomised controlled trial including the collection of economicdata. The trial design framework is exploratory**.** Alongside the trial, qualitative work is carried out to understand the feasibility of the intervention and the trial procedures.

Table 1: WHO Trial Registration Data Set

**Data category**	**Information**
Primary registry and trial identifying number	ISRCTN: 12830220
Date of registration in primary registry	21.08.2018
Secondary identifying numbers	
Source of monetary or material support	National Institute for Health Research Postdoctoral Fellowship Award
Primary sponsor	University of Manchester
Secondary sponsor	N/A
Contact for public queries	Helen.hawley-hague@manchester.ac.uk
Contact for scientific queries	Helen.hawley-hague@manchester.ac.uk
Public title	The Together trial
Scientific Title	Can smartphone **T**echnol**OG**y be used to support an **E**ffec**T**ive **H**ome **E**xe**R**cise intervention to prevent falls amongst community dwelling older people?<br>The **TOGETHER** feasibility RCT.
Countries of recruitment	UK
Health condition of problem studied	Falls in Older Adults
Interventions	**Standard service:**<br>**Manchester City**: 12 weeks once a week contact (home or group exercise), check-ups until 6 months discharge.<br>**Trafford:** 8 weeks group exercise once a week or 6 week home exercise then discharged or referred to further 8 week group exercise.

For peer review only - http://bmjopen.bmj.com/site/about/guidelines.xhtml

	For all prescribed exercise plan and exercise booklet given, asked informally what they want to achieve (outcome goals).<br>Use of study provided smartphone for reporting exercises and falls detection as outcome measures only<br><br>**Intervention:** Standard service plus the use of Motivate Me (health professional app) and My Activity Programme (patient app) on study provided smartphones.
Key inclusion and exclusion criteria	**Age:** Older adults aged 50+<br>**Sex:** Male or female<br>**Inclusion:** At risk of falls, referred to falls rehabilitation services and assessed as suitable for an exercise programme, Good 3G/4G reception in their home or wifi.<br>**Exclusion:** unable to follow instructions (unless they have support from a family member or carer), Severe visual impairment, long-term residential or nursing care, terminal illness or expected shortened lifespan, defined as less than 6 months, Older adults unable to read written English unless they have support from a family member or carer).
Study type	Interventional<br>Allocation: randomised;<br>Primary purpose: prevention, feasibility
Date of first enrolment:	September 2018
Target sample size	72
Recruitment status	Pending
Primary outcome	Feasibility of the design and procedures
Key secondary outcomes	Balance (Berg), Function (TUG/mTUG), Falls (Calendar/FallsMonitor@home), Strength (30 second chair stand), Fear of Falling (Short FES-I), Health related quality of life (EQ5D-5L/ ICE-CAP-O), resource use, adherence (my activity programme/EARS).<br>Baseline, 3, 6 months.

For peer review only - http://bmjopen.bmj.com/site/about/guidelines.xhtml

Sampling principles and procedures

Eligibility

Older adults at risk of falls (aged 50+ years) and assessed as requiring a falls rehabilitation exercise programme, are identified through current community falls rehabilitation services delivered in Manchester City and Trafford. The two sites see patients from diverse socio-economic populations. Exclusions include older adults who are unable to follow instructions (unless supported by a family member/carer), who are unable to understand written English (unless supported by a family member/carer), with severe visual impairment, those in long-term residential or nursing care and those with terminal illness or expected shortened lifespan, defined as less than 6 months, as determined by the NHS teams. Patients need to have good 3G/4G mobile phone reception (able to access webpages) or wifi in their home and this is assessed by the health professional before they handout participant information or by the researcher when taking consent.

Recruitment, consent, sample size

Health professionals give patients the study information sheet and inform them about the intervention. The health professionals then ask the patient if they are happy to be contacted by the researcher who demonstrates the technology either in the patient's home or within groups at each NHS site. The technology is demonstrated to the participant before they are asked to give informed consent. Where possible a former patient who has used the smartphone applications accompanies the researcher to demonstrate the technology. We think involvement of a peer has the potential to assist in promoting patient confidence in the use of the technology.

The first 36 eligible patients identified through each service (N=72 in total) who are willing to participate are being recruited and randomised (Figure 1). Thirty patients per arm after attrition (approx. 10%) are normally used for feasibility RCTs[26]. Study participants are randomised using a computer-generated randomisation algorithm at sealedenvelope.com, stratified by gender and site, using block randomisation (2, 4, 6 blocks) into either intervention or control group.

Blinding

Baseline and follow-up (3 and 6 months) assessments are carried out by experienced clinicians within each NHS Trust (not a member of the clinical teams participating), who are blinded to which intervention the participants are receiving, at baseline the individual is also blinded to the intervention they will receive as randomisation occurs after baseline assessment.

As this is an 'active' intervention, it is not possible to blind the health professionals delivering the service or the participants during the intervention. The lead researcher provides technical support to both arms to use the smartphone so is not blinded.
The statistical analysis will be carried out by the lead researcher with the support of a statistician. Patient ID codes will be removed from the data to allow for blinded analysis.

Patient withdrawal

In consenting to the trial, patients are consenting to the trial treatment, follow-up and data collection. If withdrawal of the randomly allocated treatment occurs, patients should still be followed up where they agree. Patients are allowed to withdraw without giving reason

For peer review only - http://bmjopen.bmj.com/site/about/guidelines.xhtml

at any time and a withdrawal case report form (CRF) will be completed to document the date and reason (where given) for withdrawal. Data collected up to the time of withdrawal will be included in analyses. Health professionals will assess patients' capacity to take part in the rehabilitation programme and the study, if they have been deemed to have lost capacity to consent they will be withdrawn from the study but the data already collected will be retained.

Interviews with patients

All participants are offered an interview (even those who withdraw from the trial) in their own home after the final follow-up to assess their experiences of the intervention and trial processes. Family members/carers may also attend the interview at the participants' request.

Focus groups with health professionals

Health professionals from Trafford and Manchester city who are involved in the study are recruited to participate in a focus group at the end of the study (after 24 weeks follow-up). All members of staff (N=8) will be given study information by their team leader and asked if they are available for a focus group, the focus groups will take part at their place of work at a time convenient to each team. Participating staff can choose to be part of a one-to-one interview if they prefer not to be interviewed with colleagues or if for staffing reasons it is not feasible for them to attend the focus group.

The Intervention

Full details of the intervention components are shown in Supplementary material: Table 1 (TIDieR Guidelines).

The technology

The Samsung Galaxy J5 as a means of communication[27] will be provided to all participants and health professionals. Samsung phones have been used previously in our research, with good usability and have the correct specification for the falls detector to work [10]. The research team will provide technical support for participants and health professionals (HHH) and any required application updates (SM, CT).

'Motivate me' app

The 'Motivate me' app is the health professional application. This app is used by the health professional with the patient to set behavioural and outcome-based goals, for the health professional to see what exercises the patient has reported and to give feedback and to check they have received messages (Supplementary Material Figure 1).

'My activity programme'

'My activity programme' is the patients application. This app will be used by the patient to report the exercises they have done, receive messages and prompts and to confirm whether they like the messages received (Supplementary Material Figure 2).

For peer review only - http://bmjopen.bmj.com/site/about/guidelines.xhtml

There are 12-behaviour change techniques adopted[28] through the intervention include goal-setting (behaviour/outcome), action-planning (recording plan to exercise in diary on smartphone/reminder text messages when it is time to start the programme), and feedback on behaviour (providing feedback on what they have done/benefits). The key behavioural change techniques delivered as part of the control and intervention arms are outlined in Table 2.

Table 2: Behaviour change techniques adopted*

	**1.Intervention arm**	**1a How**	**2.Control arm (standard service)**	**2a How**
1.1 Goal setting (behaviour)	x	what, when, where-smartphone and paper	x	What, Where-Paper
1.3 Goal setting (outcome)	x	smartphone verbally	x	Verbally
1.4 Action planning	x	smartphone		
1.5 Review Behavioural goals	x	smartphone verbally	x	Paper Verbally
1.7 Review outcome goals	x	smartphone verbally	x	Verbally
2.2 Feedback on behaviour	x	Smartphone verbally	x	Verbally
4.1 Instructions on how to perform the behaviour	x	Physically<br>Smartphone Paper	x	Physically<br>Paper
5.1 Information about health consequences	x	Smartphone Verbally (ad hoc)	x	Verbally (ad hoc)
5.6 Emotional Consequences	x	Smartphones Verbally (ad hoc)	x	Verbally (ad hoc)

For peer review only - http://bmjopen.bmj.com/site/about/guidelines.xhtml

6.1 Demonstration of behaviour	x	Physically	x	Physically
7.1 Prompts	x	Smartphone		
8.7 Graded tasks	x	Smartphone<br>Paper	x	Paper

*Based on Michie et al[28] behaviour change taxonomy

The Control

Standard service is variable across different sites, but all sites deliver a mix of the evidence-based Falls Management Programme (FaME) and Otago[29] exercises as standard care.

They include face-to-face delivery once-a-week and a prescribed home-exercise programme (with booklet) with informal outcome-based goal-setting.

Manchester City: Once a week visits (either home-based or group exercise) for around 12 weeks (dependent on need) and then check-ups until 6 months discharge.

Trafford: 8 weeks group exercise once a week then discharged, or 6 week home-based exercise then discharged or referred to further 8 week group exercise.

Both sites leave participants with a home exercise plan on discharge and where appropriate refer onto community-based strength and balance programmes.

Control application for self-reporting exercise

The control arm receives a study phone with a basic app where they report their exercises, but they are only able to report their exercises (outcome measure), they are not able to view their programme, receive messages or receive feedback on the phone. The health professional is not able to view what they have reported (outcome measure for the research team only).

Co-treatments

Trial participants are free to seek management of falls and other related or unrelated medical conditions during the course of the trial. We record all health service resource use and these will be reported as a trial outcome. At trial closure, participants will continue with usual healthcare, no further ancillary care is provided beyond that immediately required for the proper and safe conduct of the trial.

Outcome measures

Data such as demographics (age, gender, socio-economics, health conditions, falls history, previous smartphone/mobile phone use and wifi) and physical tests are recorded on the

For peer review only - http://bmjopen.bmj.com/site/about/guidelines.xhtml

CRF (Table 3).

Table 3: Schedule of enrolment interventions and assessments

	STUDY PERIOD					
	**Enrolment**	**Allocation**	**Post-allocation**			**Post intervention**
**TIMEPOINT****	**$-t_1$**	**0**	**T_1**	**T_2**	**T_3**	
**ENROLMENT:**						
**Eligibility screen**	X					
**Consent to further information**	X					
**Tech demo and Informed consent**	X					
**Allocation**		X				
**INTERVENTIONS:**						
Control: ***CFS***			⟷	⟷	⟷	
TCS			⟷			
Intervention			⟷	⟷	⟷	
**ASSESSMENTS:**						
**Gender**<br>**Age**<br>**Ethnicity**<br>**Education**<br>**Housing**<br>**Falls history**<br>**Medical history**<br>**Previous mobile/smartphone use**<br>**Allocated to home or group exercise**	X					
Falls (Calendar)<br>***Falls (alarm)***<br>***My activity self-report***<br>***Prescribed exercise plan***<br>***Face to Face delivery***			⟷	⟷	⟷	
Berg<br>***TUG***<br>***mTUG***<br>***30 Second Chair Stand***<br>***FES-1***<br>***EQ5D***<br>***Resource Use***<br>***Health professional time resource***	X			X	X	

For peer review only - http://bmjopen.bmj.com/site/about/guidelines.xhtml

ICE-CAP-O EARS						
Interviews Focus groups						X

Primary outcome measures

Assess feasibility and acceptability of the design and procedures including:

a. willingness of participants to be randomised (collected on the screening section of the CRF/interviews)
b. willingness of clinicians to recruit participants (through focus group/completion of screening on the CRF)
c. number of eligible patients (through the CRF and documentation of number of monthly referrals to falls services)
d. whether demonstration by peer of the technology aids recruitment.
e. characteristics of the proposed outcome measures e.g. reliability of falls detector when compared to falls calendars, whether a self-report app is a reliable outcome measure.
f. follow-up rates, adherence/compliance rates
g. time needed to collect and analyse data
h. determine effect sizes for use in sample-size calculations, enabling power calculations for the reduction in falls for a definitive large scale RCT.

Start/stop criteria for going to full trial are included in CONSORT diagram (Diagram 1) based on these outcome measures.

We will also report intervention fidelity, process and compliance using observation during quality assurance visits. Health professionals and the assessors will follow a Standard Operating Procedure (SOP) for assessment and intervention. The trial treatment record (CRF) includes details of the grade and type of staff involved with delivery. The health professionals and researcher will keep an issue-log of technical issues with the phones or apps during the trial either experienced directly or reported by participants. We will also explore the potential impact of differing length of exercise delivery across sites.

For peer review only

Outcome measures

Falls

The primary outcome for any future definitive trial would be falls, expressed as fall rate per person per months of follow-up observation after randomisation. The current study collects falls data for the purposes of testing feasibility of data collection, and to inform us of falls rates and intervention effect size for a future sample size calculation.

All participants will wear the smartphone in their pocket or on a waistband and this will act as a falls detector, running the FallsMonitor@home app developed by the University of Bologna[10]. If the patient chooses then they can also use the app on the phone as a falls alarm. The fall detection system application allows the user to identify a list of formal/informal caregivers who will receive an SMS if a fall is detected. Patients are given

For peer review only - http://bmjopen.bmj.com/site/about/guidelines.xhtml

an opportunity to de-active the falls alarm through an application on the smartphone if there is a false alarm, enabling the user to maintain control and prevent unwanted intrusion. Participants are asked if we can use their anonymised falls data for further development of the app and in the Farseeing real-world falls database[30].

To validate this as an outcome measure we use the internationally agreed ProFaNE falls definition[31] and follow the agreed ProFaNE falls data collection and analysis protocols based on self-report calendars[32].

Fear of falling
Short Falls Efficacy Scale-International (Short FES-1) is used to measure fear of falling[33]. This is often a measure used by UK falls services as part of standard outcome measures.

Function
The Timed Up and Go test (TUG) will be used to assess improvements in mobility and function. The TUG will be applied as described by Podsiadlo and Richardson[25]. Participants will be asked to perform the TUG at their self-selected habitual walking speed. A medical device implementing an instrumented version of the TUG will be used (mTUG, mHealth Technologies s.r.l., Bologna, Italy). The device is able to automatically provide guidance to the user for administering the test, capture and process the data, and generates summary reports of function for the health professional. The blinded assessor will complete the normal TUG and the mTUG as outcome measures (the standard TUG as a validation measure) to explore whether the mTUG is usable as an outcome measurement for the definitive RCT. The health professional will carry out the mTUG with a sub-sample of 10 patients at each site to assess their experiences of its use.

Balance
The Berg Balance Scale will be used to assess balance. This has good validity and sensitivity in this population[34] and is one of the best outcome measures for assessing standing balance[35]. It has also been used for the prediction of falls[36]. The effect sizes from this outcome measure scale will be used as part of the power calculation for the full trial.

Strength
30 seconds chair stand test[37], which has good validity and is used throughout health services will be used to assess physical ability, in particular strength.

Adherence
Adherence will be measured in a number of ways (outlined in detail, Table 4):
1) Self-report app will be used for both control and intervention group. Adherence will be classed as the participant carrying out 80% of their prescribed programme (based on the evidence-base for effective strength and balance)[6,38].
2) Exercise Adherence Rating Scale (EARS)[39]. This is a validated 16-question tool with a 6-question subscale specifically measuring adherence (remaining questions measure reasons for adherence/non-adherence).

Health economics
The health related quality of life measures will include the European Quality of Life 5

For peer review only - http://bmjopen.bmj.com/site/about/guidelines.xhtml

Dimensions (EQ-5D-5L)[40] and an additional measure used in previous trials related to falls prevention (the ICEpop CAPability measure for Older people (ICE-CAP-O)[41,42]. Costs of delivering the intervention will be observed based on staff training, delivery costs and equipment costs. Additional resource use measures will be captured via a Resource Use Questionnaire which will seek to measure costs related to an NHS and social care perspective (secondary, primary, community care service use), and a patient perspective (costs related to informal care). The findings from these will inform the feasibility of collection of the data, and priorities for cost collection at full trial.

For peer review only

For peer review only - http://bmjopen.bmj.com/site/about/guidelines.xhtml

Table 4: Adherence measures

	What	How/additional validation
**Self-report through my activity programme and control arm smartphone app**	Exercises reported on app to their prescribed programme they day they are carried out.<br>- Exercise Type<br>- Intensity<br>- Dose<br>Adherence defined as participant carrying out 80% of their prescribed programme.	The health professional will be asked to provide a copy of the participants prescribed exercise plan, any changes to it and the dates any changes were made (both sites record this as part of standard intervention).<br><br>For face to face home delivery, the health professional will be asked to report exactly what the patient has done when with them (this will be used to validate the self-report from participants).<br><br>After discharge from rehabilitation if participants move onto other strength and balance provision. Those services will give us copies of the exercise programme delivered for any days the participants attend, attendance records and any prescribed home exercise programme.
**EARS**	Validated 16-question tool with a 6-question subscale specifically measuring adherence.	Paper questionnaire at baseline, 3 months and 6 months.

Interviews/focus groups

The interview and focus group schedules are based on FARSEEING guidelines[43]. The following key areas will be explored in relation to the smartphone, the 'Motivate me' app, 'My activity programme' app, FallsMonitor@home, and the mTUG. Ease of use, clarity of screen, demonstration of use, wearing comfort, adaption of use, reliability, choice and control and home and lifestyle. This feedback will be considered and any required changes to the technology set-up, applications and intervention will be made prior to the definitive RCT. We also ask additional questions about the research process including: general expectations and views; experiences of recruiting patients (health professional), and of being recruited and randomised (patients), suggestions of methods for recruiting participants; likely uptake and retention of participants.

For peer review only - http://bmjopen.bmj.com/site/about/guidelines.xhtml

Analysis

Quantitative data is analysed using SPSS Release 22.0. The main analyses is descriptive, involving the estimation of recruitment rates, attrition rates, non-compliance rates, means and standard deviations of outcomes by group at baseline and end trial, and 95% confidence intervals for differences of means of outcomes between groups and assessment of change following the intervention at end trial. The health economics analysis is focussed on informing relevant measures and means of collection of health related quality of life and resource use for the future definitive study. Only an exploratory cost-effectiveness analysis will be conducted, for all measures we will report mean values and sample variability alongside information on missing values.

Data from the smartphone based outcome measures (FallsMonitor@home, mTUG, My activity programme/control self-report app) will be compared to the traditional measures (falls calendar[32], TUG[25], EARS[39]) alongside qualitative feedback as part of their validation.
A statistical analysis plan will be created before data analysis.

Qualitative interviews/focus groups will be analysed using thematic analysis[44]. The research will be inductive and although will seek to further understand the quantitative findings, this approach will also generate categories and explanations directly from the data rather than based on previously set aims and objectives, reducing risk of bias[44]. QSR International's NVivo 10 qualitative data analysis software will be used to manage the data. The validity of the analysis will be checked by returning to the data once themes have been identified and also through the use of a second researcher who will check samples of analysis. The accuracy of the transcripts will be checked through discussion with participants to establish if anything is not clear from the interviews/focus groups.

Ethical issues

Ethical approval has been granted from the North West Greater Manchester East Research Ethics Committee (Rec ref: 18/NW/0457, 9/07/2018). Regional and site-specific approvals have been obtained. As this is a study with older patients a number of ethical issues could arise. To address these, community services will act as gatekeepers to access patients and assess patients' eligibility for the study. The intervention is delivered by health service staff and provided in addition to standard service, therefore patients are unlikely to be disadvantaged.

If falls are detected by the smartphone, it is important that someone is informed in real-time. The smartphone application allows the user to select a list of formal/informal caregivers who will receive an SMS if a fall is detected. It will be made clear that the falls service is not an emergency service so in the event of a fall the person receiving the text message would call an ambulance as they would in normal circumstances. If patients already wear a call alarm then they will be encouraged to continue to use this as well or to adopt their usual method of alerting help.

The study requires monitoring of subjects and it is important that patients do not find this

For peer review only - http://bmjopen.bmj.com/site/about/guidelines.xhtml

obtrusive (privacy issues have been identified as major barriers to the use of technology). Patients are given an opportunity to de-active the falls alarm through an application on the smartphone if there is a false alarm. However, previous consultation/usability testing with older adults raised no major privacy issues.

There are ethical issues in the removal of technology at the end of studies[45]. We will not be able to offer older adults the technology at the end of the 6 month study period, but they will be offered the opportunity to download the apps onto their own phones if they wish.

The risk of interviews and focus groups are minimal. The patient or health professional can ask the researcher to move onto another question if they are uncomfortable at any point. Health professionals will be given the chance to discuss the trial, technology and intervention in a one-to-one interview if they do not feel comfortable giving feedback in front of colleagues.

Patient and public involvement

Patient and public representatives have been involved in designing the trial including outcome measures. Feedback from previous usability testing with patients and from patients who sit on our Advisory Group (AG) provided direct information on the design of the trial e.g. use of self-report app for control arm. Patients on our AG (who were formerly patients of one of the services) helped to design study material such as the patient information sheet. They assisted in training health professionals in approaching patients for recruitment and goal-setting as part of the intervention. Three participants', who took part in our usability testing, became peer mentor volunteers for the trial. They will attend the first visit (if the patient gives permission) to demonstrate the technology to patients before consent is given. We will explore whether peer involvement aids recruitment. Finally, the volunteers and the patients who sit on our AG will aid with dissemination of study findings e.g. helping to arrange dissemination events and providing feedback on newsletters for participants.

Trial monitoring

The lead researcher (HHH) will monitor the delivery of the intervention and recruitment of patients, there will also be a clinical lead (AE, EM) at each site taking overall responsibility for identification of patients and delivery of the intervention. This team, alongside academic experts (JH, LC, SM, ASM, CT) from the Trial Co-ordination Group will ensure overall quality of trial data. The AG, which meets bi-annually, giving feedback on the project, providing expert guidance and assisting in dissemination, this includes two previous patients. A risk register is reviewed by the AG. The study is subject to the audit and monitoring regime of the University of Manchester and a monitoring plan followed.

A detailed risk assessment has been carried out and potential patient, organisational and study hazards considered, the likelihood of their occurrence and the resulting impact should they occur.

Adverse events

A safety reporting protocol has been developed for related and unexpected serious adverse events (AEs) and directly attributable AEs. An AE is defined as any untoward medical

For peer review only - http://bmjopen.bmj.com/site/about/guidelines.xhtml

For peer review only

occurrence in a subject which does not necessarily have a causal relationship with treatment. These AEs are recorded in the CRF and if a Serious Adverse Event (SAE) occurs then reported to the Chief investigator. The CI will determine whether AEs require reporting to the trial sponsor and Ethics Committee, in accordance with the safety reporting protocol.

DISCUSSION

This is the first trial that we are aware of that explores the potential use of motivational smartphone apps for the support of an evidence-based falls exercise programme. As this is an active intervention and control we are unable to blind participants or those delivering the intervention. However, the design does enable us to blind both those carrying out the assessments and analysis.

We provide participants with study phones, which may be different to using the app on their own phones. However, we need to ensure the smartphone meets the technical specification required for FallsMonitor@home to work correctly. Furthermore use of study phones enables us to maintain confidentiality of participants (if phones are lost we can wipe them remotely).
This trial assesses several novel outcome measures against the gold standard, the mTUG against standard TUG, the FallsMonitor@home against standard calendar method and a self-report app against the EARS tool[39]. This enables us to further our understanding of whether technology has the potential to provide more objective and reliable outcome measures than current methods.

We use two very different NHS sites, reflecting the reality of day-to-day practice (one specialist falls service, one general rehabilitation services) to explore the delivery of the intervention. This means that the standard service is different across the two sites adding complexity to how the control and intervention arm are delivered. However, these differences enable us to assess its scalability to full trial where different types of falls services would need to be included as sites. It also enables us to be more representative of current services and assess its potential for delivery in practice.

Figure 1: Consort diagram

Supplementary Material Figure 1: Motivate Me user interface

Supplementary Material Figure 2: My Activity Programme interface

For peer review only - http://bmjopen.bmj.com/site/about/guidelines.xhtml

Authors' contributions:

HHH leads the research project and its design, managing the trial overall and has led the writing of the protocol. CT and SM give technical support for the study and have advised on outcomes and the manuscript. JH, LC, ASM, CT and SM have provided scientific advice around the design of the study and commented on the manuscript. ST and YF have given advice on statistics and health economic part of design and manuscript. AE and EM have given advice on the operationalisation of the study and commented on the manuscript.

Acknowledgments

We thank Professor Dawn Skelton and Later Life Training for allowing us to use their images and the name 'Motivate Me' for the health professional app. We thank two PPI representatives who sit on our Advisory Group and three peer volunteers who are supporting recruitment.

Competing Interests

No competing interests declared.

Funding
This study is independent research arising from a NIHR Postdoctoral Fellowship Award, Dr Helen Hawley-Hague, (PDF -2015-08-012) supported by the National Institute for Health Research**.** The views expressed in this publication are those of the author(s) and not necessarily those of the NHS, the National Institute for Health Research or the Department of Health.

only

For peer review only - http://bmjopen.bmj.com/site/about/guidelines.xhtml

REFERENCES

1. Public Health England. Muscle and bone strengthening and balance activities for general health benefits in adults and older adults. Summary of a rapid evidence review for the UK Chief Medical Officers' update of the physical activity guidelines. London 2018. https://assets.publishing.service.gov.uk/government/uploads/system/uploads/attachment_data/file/721874/MBSBA_evidence_review.pdf (accessed 15 November 2018)
2. Yardley, L., Kirby, S., Ben-Shlomo, Y, et al. How likely are older people to take up different falls prevention activities? *Prev Med*2008;47:554-58. doi: 10.1016/j.ypmed.2008.09.001
3. Gillespie, L.D., Robertson, M.C., Gillespie, W.J, et al. Interventions for preventing falls in older people living in the community. *Cochrane Database of Systematic Reviews.* 9, 2012 Art. No.: CD007146. doi: 10.1002/14651858.CD007146.pub3.
4. Sherrington, C., Tiedemann, A., Fairhall N, et al. Exercise to prevent falls in older adults: an updated systematic review and meta-analysis. *Br J Sports Med*2018;51:1750-1758. doi: 10.1136/bjsports-2016-096547
5. Public Health England (2018) An ROI Tool: Assessment of Falls Prevention Programmes for Older People Living in the Community. Crown Copyright. https://assets.publishing.service.gov.uk/government/uploads/system/uploads/attachment_data/file/679856/A_return_on_investment_tool_for_falls_prevention_programmes.pdf (accessed 6 August 2018)
6. Nyman, S.R. Victor, C.R. Older people's participation in and engagement with falls prevention interventions in community settings: an augment to the Cochrane systematic review. *Age Ageing*2011;41:16-23. doi: 10.1093/ageing/afr103.
7. Royal College of Physicians. Older people's experiences of therapeutic exercise as part of a falls prevention service-patient and public involvement. London 2012: RCP.
8. Rapp, K., Büchele,G., Dreinhöfer, k, et al. Epidemiology of hip fractures. *Z Gerontol Geriatr*, 2018: 1-7. https://doi.org/10.1007/s00391-018-1382-z.
9. Ofcom. Rise of the social seniors revealed. https://www.ofcom.org.uk/about-ofcom/latest/media/media-releases/2017/rise-social-seniors (accessed 12 June 2019).
10. Mellone, S., Tacconi., C, Schwickert., L. Smartphone-based solutions for fall detection and prevention: the FARSEEING approach. *Z Gerontol Geriatr*2012;45:722-7. doi: 10.1007/s00391-012-0404-5.
11. King, A.C., Hekler, E.B., Grieco, L.A, et al. Harnessing Different Motivational Frames via Mobile Phones to Promote Daily Physical Activity and Reduce Sedentary Behavior in Aging Adults. *PlosOne*2013;8. DOI: 10.1371/journal.pone.0062613
12. del Rosario, MB., Redmond, SJ., Lovell, N,H. Tracking the Evolution of Smartphone Sensing for Monitoring Human Movement. *Sensors (Basel)*. 2015. 15: 18901–18933. doi: 10.3390/s150818901
13. Hamm, J., Money, AG., Atwal, A, et al. Fall prevention intervention technologies: a conceptual framework and survey of the state of the art *J. Biomed. Inform*2016;59:319-345. https://doi.org/10.1016/j.jbi.2015.12.013
14. CONFIDENCE (2011) D8.5: Final report. http://www.confidence-eu.org/ (accessed 8 August 2014)
15. Parker, S.J., Jessel, S., Richardson, J.E., Reid, C.M. Older adults are mobile too! Identifying the barriers and facilitators to older adults' use of mHealth for pain management. *BMC Geriatrics* 2013, 13:43. https://doi.org/10.1186/1471-2318-13-43.
16. Fukuoka, Y., Kamitani, E., Bonnet, K, et al. Real-Time Social Support Through a Mobile Virtual Community to Improve Healthy Behavior in Overweight and Sedentary

For peer review only - http://bmjopen.bmj.com/site/about/guidelines.xhtml

Adults: A Focus Group Analysis. *J Med Internet Res*2011;13:e49.
17. Vankipuram, M., McMahon., S., Fleury, J. ReadySteady: App for Accelerometer-based Activity Monitoring and Wellness-Motivation Feedback System for Older Adults. *AMIA Annual Symposium Proceedings*2012:931–939.
18. Worringham C, Rojek A, Stewart I, et al.Development and Feasibility of a Smartphone. ECG and GPS Based System for Remotely Monitoring Exercise in Cardiac Rehabilitation. *PloS one*2011:6. doi: e14669-10.1371/journal.pone.0014669
19. Rabin, C. Bock, B. Desired features of smartphone applications promoting physical activity. *Telemed J E Health*2011;17:1-3. doi: 10.1089/tmj.2011.0055
20. Epton, T., Currie, S., Armitage, C. J. Unique effects of setting goals on behavior change: Systematic review and meta-analysis. *J Consult Clin Psychol*2017;85:1182-98. doi: 10.1037/ccp0000260
21. Hawley-Hague, H., Horne, M., Campbell, M, et al. Multiple levels of influence on older adults' attendance and adherence to community exercise classes. *Gerontologist*2014; 54:599-610. doi: 10.1093/geront/gnt075
22. Yardley, L., Donovan-Hall, M., Francis, C, et al. Attitudes and beliefs that predict older people's intention to undertake strength and balance training. *J Gerontol B Psychol Sci Soc Sci 2007*;2:199-225. doi: 10.1093/geronb/62.2.P119
23. Ajzen, I. *Attitudes, Personality and Behaviour*. Maidenhead, UK: Open University Press 1988.
24. Lucidi, F., Grano, C., Barbaranelli, C, et al. Social-cognitive determinants of physical activity attendance in older adults. *J Aging Phys Act2006;*14:344-59.
25. Podsiadlo, D., Richardson, S. The timed "Up & Go": a test of basic functional mobility for frail elderly persons. *J Am Geriatr Soc*1991;39:142–48.
26. Lancaster, GA. Dodd, S., Williamson, P.R. Design and analysis of pilot studies: recommendations for good practice. *J Eval Clin Pract* 2004; 10:307-12. doi:10.1111/j.2002.384.doc.x
27. Boulton, E. Hawley-Hague, H., Vereijken, B, et al. Developing the FARSEEING Taxonomy of Technologies: Classification and Description of Technology Use (including ICT) in Falls Prevention Studies. *J Biomed Inform* 2016; 61:132-140. doi: 10.1016/j.jbi.2016.03.017
28. Michie, S., Richardson, M., Johnston, M, et al. The behavior change technique taxonomy (v1) of 93 hierarchically clustered techniques: building an international consensus for the reporting of behavior change interventions. *Ann Behav Med*2013;46:81-95. doi: 10.1007/s12160-013-9486-6
29. ProFouND. Strength and Balance Home Exercise Booklet for Older People (English) 2013. http://profound.eu.com/strength-and-balance-home-exercise-booklet-for-older-people-english/ (accessed 8 November 2018)
http://profound.eu.com/otago-exercise-program/#
30. FARSEEING. The FARSEEING real world falls repository, http://farseeingresearch.eu/the-farseeing-real-world-fall-repository-a-large-scale-collaborative-database-to-collect-and-share-sensor-signals-from-real-world-falls/ (accessed 5 August 2018)
31. Lamb S, Jørstad-Stein, EC, Hauer K, et al. Development of a common outcome data set for fall injury prevention trials: the Prevention of Falls Network. Europe consensus *JAGS*,2005;53:1618–1622 doi:10.1111/j.1532-5415.2005.53455
32. Hauer K, Lamb SE, Jorstad EC, et al. Systematic review of definitions and methods of measuring in randomized controlled fall prevention trials. *Age Ageing,*2006;35:5-10. doi: 10.1093/ageing/afi218

For peer review only - http://bmjopen.bmj.com/site/about/guidelines.xhtml

33. Kempen G, Yardley, L Van Haastregt, J.C.M, et al. The Short FES-I: a shortened version of the Falls Efficacy Scale-International to assess fear of falling. *Age Ageing*.2008;37:45-50 doi: https://doi.org/10.1093/ageing/afm157
34. Berg K,O., Wood-Dauphinée, SL., Williams, JI, et al. Measuring balance in the elderly: validation of an instrument. *Can J Public Health*1992; S2:S7–S11.
35. Sibley KM, Howe T, Lamb SE, et al. Recommendations for a Core Outcome Set for Measuring Standing Balance in Adult Populations: A Consensus-Based Approach. *Plos1*2015. https://doi.org/10.1371/journal.pone.0120568
36. Lajoie, Y., Gallagher, S.P. Predicting falls within the elderly community: comparison of postural sway, reaction time, the Berg Balance Scale and the Activities-specific Balance Confidence (ABC) scale for comparing fallers and non-fallers. *Arch Gerontol Geriatr*2004;38:11-26
37. Jones CJ, Rikli RE, Beam WC. A 30-s chair-stand test as a measure of lower body strength in community-residing older adults. *Res Q Exerc Sport*1999;70:113–19. doi: 10.1080/02701367.1999.10608028.
38. Osho O, Owoeye O, Armijo-Olivo S. Adherence and Attrition in Fall Prevention Exercise Programs for Community-Dwelling Older Adults: A Systematic Review and Meta-Analysis. *J Aging Phys Act* 2017. 3:1-34. doi: 10.1123/japa.2016-0326
39. Newman-Beinart, N., Norton, S., Dowling, D, et al. The development and initial psychometric evaluation of a measure assessing adherence to prescribed exercise: the Exercise Adherence Rating Scale (EARS). *Physiotherapy*2017;103:180-85. doi: 10.1016/j.physio.2016.11.001
40. Janssen MF, Pickard AS, Golicki D, et al. Measurement properties of the EQ-5D-5L compared to the EQ-5D-3L across eight patient groups: a multi-country study. *Qual Life Res* 2013;22:1717-1727. doi: 10.1007/s11136-012-0322-4
41. Grewal I, Lewis J, Flynn T, et al. Developing attributes for a generic quality of life measure for older people: Preferences or capabilities? *Soc Sci Med*. 2006;62:1891-1901
42. Davis J, Liu-Ambrose T, Richardson C, et al. A comparison of the ICECAP-O with EQ-5D in falls prevention clinical setting: are they complements or substitutes? *Qual life Res*2012;22:969-77. doi: 10.1007/s11136-012-0225-4
43. FARSEEING**.** Preliminary guidelines for design and implementation of technologies 2014. http://farseeingresearch.eu/wp-content/uploads/2014/07/Preliminary-Guidelines-V1.0-Public.pdf (accessed 8 August 2017)
44.Braun V. Clarke V. Using thematic analysis in psychology. *Qualitative Research in Psychology* 2006;3:77-101. doi.org/10.1191/1478088706qp063oa
45. Magnusson, L., Hanson, E.J. Ethical issues arising from a research, technology and development project to support frail older people and their family carers at home. *Health Soc Care Community* 2003;11:431-9.

For peer review only - http://bmjopen.bmj.com/site/about/guidelines.xhtml

For peer review only

Feasibility RCT

Criteria to judge the feasibility of progression to a definitive RCT based on:

1. ≥30% of eligible patients willing to be recruited to feasibility study;
2. ≥80% of patients complete the intervention
3. Data collected on key outcomes at 6-months follow-up for ≥70% of participants;
4. <10% of Serious Adverse Events deemed due to the intervention itself.

Feasibility RCT (2 sites recruiting to both arms)

Baseline assessments

Randomisation

Intervention: Allocated to standard service/usual care with the addition of smartphone with motivational apps

- Trafford N= 18
- Manchester city N= 18

Control: Allocated to standard service/ usual care Receive smartphone for outcome measures only

- Trafford N= 18
- Manchester city N= 18

3 month follow-up, 6 month follow-up

Interviews (Health Professionals, N=8),
Focus groups/Interviews (N=60 patients) **at 6 months**

For peer review only

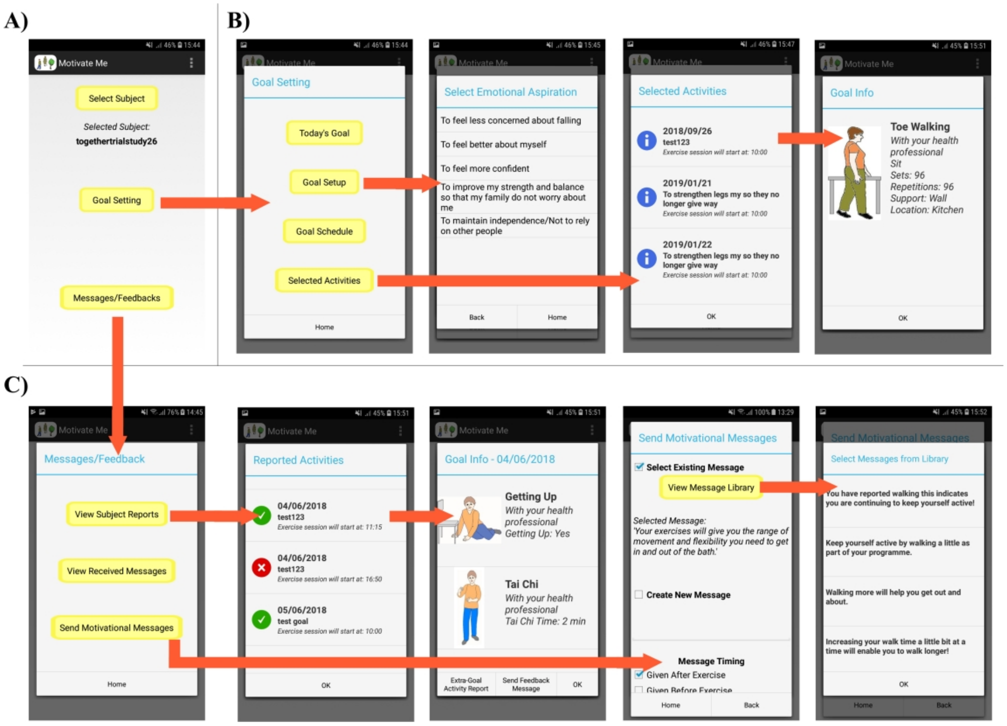
A)
B)
C)
Motivate Me
Select Subject
Selected Subject:
togethertrialstudy26
Goal Setting
Messages/Feedbacks
Goal Setting
Today's Goal
Goal Setup
Goal Schedule
Selected Activities
Home
Select Emotional Aspiration
To feel less concerned about falling
To feel better about myself
To feel more confident
To improve my strength and balance so that my family do not worry about me
To maintain independence/Not to rely on other people
Back
Home
Selected Activities
2018/09/26
test123
2019/01/21
To strengthen legs my so they no longer give way
2019/01/22
To strengthen legs my so they no longer give way
OK
Goal Info
Toe Walking
With your health professional
Sit
Sets: 96
Repetitions: 96
Support: Wall
Location: Kitchen
OK
Messages/Feedback
View Subject Reports
View Received Messages
Send Motivational Messages
Home
Reported Activities
04/06/2018
test123
04/06/2018
test123
05/06/2018
test goal
OK
Goal Info - 04/06/2018
Getting Up
With your health professional
Getting Up: Yes
Tai Chi
With your health professional
Tai Chi Time: 2 min
Extra-Goal Activity Report
Send Feedback Message
OK
Send Motivational Messages
Select Existing Message
View Message Library
Selected Message:
'Your exercises will give you the range of movement and flexibility you need to get in and out of the bath.'
Create New Message
Message Timing
Given After Exercise
Home
Back
Select Messages from Library
You have reported walking this indicates you are continuing to keep yourself active!
Keep yourself active by walking a little as part of your programme.
Walking more will help you get out and about.
Increasing your walk time a little bit at a time will enable you to walk longer!
OK
Home
Back

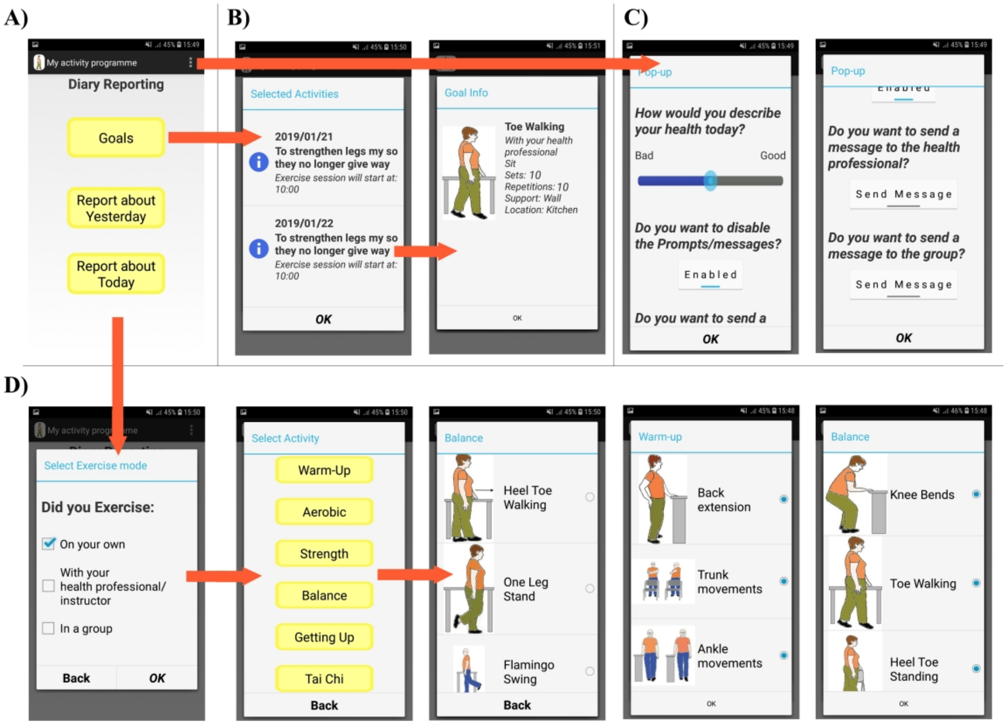
A)
B)
C)
D)
My activity programme
Diary Reporting
Goals
Report about Yesterday
Report about Today
Selected Activities
2019/01/21
To strengthen legs my so they no longer give way
Exercise session will start at: 10:00
2019/01/22
To strengthen legs my so they no longer give way
Exercise session will start at: 10:00
OK
Goal Info
Toe Walking
With your health professional
Sit
Sets: 10
Repetitions: 10
Support: Wall
Location: Kitchen
OK
Pop-up
How would you describe your health today?
Bad
Good
Do you want to disable the Prompts/messages?
Enabled
Do you want to send a
OK
Pop-up
Do you want to send a message to the health professional?
Send Message
Do you want to send a message to the group?
Send Message
OK
Select Exercise mode
Did you Exercise:
On your own
With your health professional/ instructor
In a group
Back
OK
Select Activity
Warm-Up
Aerobic
Strength
Balance
Getting Up
Tai Chi
Back
Balance
Heel Toe Walking
One Leg Stand
Flamingo Swing
Back
Warm-up
Back extension
Trunk movements
Ankle movements
OK
Balance
Knee Bends
Toe Walking
Heel Toe Standing
OK

Supplementary Material Table 1: Intervention description using the Template for Intervention Description and Replication (TIDieR) checklist*

**Item 1: Name**	Can smartphone **T**echnol**OG**y be used to support an **E**ffec**T**ive **H**ome **E**xe**R**cise intervention to prevent falls amongst community dwelling older people: The TOGETHER trial	
**Item 2: Why**	Strength and balance training has been found to be effective in reducing the rate and risk of falls. Health services are often unable to deliver the evidence-based dose of exercise and older adults do not always sufficiently adhere to their programme to gain full outcomes. This feasibility trial will explore whether smartphone technology based on the Theory of Planned Behaviour and using goal setting, feedback and prompts can be used to support patients to better adhere to an evidence-based exercise rehabilitation programme and test study procedures and outcome measures.	
**Item 3: What (Materials)**	**Intervention arm:** Behaviour change apps	**Control arm:**<br>Standard service
	Samsung Galaxy J5<br><br>**Health Professional phone based Motivate Me app**: -<br>1. set patients long-term goals (outcomes)<br>2. set patients behavioural goals (which evidence based exercises they will do and how often, when they will exercise with the health professional, alone or in a group),<br>3. access the patients self-report data and see what exercises they have been doing and when.<br>4. upgrade exercise programme.<br>5. give the patient bespoke feedback (set as once a week).<br><br>**Patient phone based My Activity Programme app:-**<br>1. report the exercises they have done (exercise type, duration, intensity)<br>2. receive prompts when they have scheduled to exercise<br>3. receive automated motivational messages based on the long-term goals they have set (with a focus on strengthening outcome	Samsung Galaxy J5<br><br>**Control group self-report app:-** report the exercises they have done (exercise type, duration, intensity)<br><br>Home exercise booklet<br><br>Calander/FallsMonitor@home<br><br>Patient 'How to guide'<br><br>Technology issue log (Health professional)

For peer review only

	expectations)<br>4. Receive bespoke feedback from health professionals (aimed at increasing Perceived Behavioural Control).<br><br>Home exercise booklet<br><br>Calander/FallsMonitor@home<br><br>Patient 'How to guide'<br>Health professional 'How to guide' (for technology)<br><br>Technology issue log (Health professional)	
**4. What procedure**	Screening and assessment as part of standard service by Falls Services<br><br>Support and training on how to use the 'My Activity Programme app' and falls alarm.<br><br>Formal goal setting session with health professional where use their 'Motivate me' app to set patients' behavioural and outcome-based goals (including prescribing the exercise programme). Handing out of home exercise booklet.<br><br>Strength and balance rehabilitation in group or home commences.<br><br>**Trafford:**<br>If recruited through Community Rehabilitation team they receive a 6- week programme. They are then either referred to group-based rehabilitation or discharged. Seen in own home once a week.<br><br>If recruited through group-based rehabilitation they receive an 8 week group-based programme. Seen in a group once a week.<br><br>They are then referred on to a community based FaME class once a week and prescribed home	Screening and assessment as part of standard service by Falls Services.<br><br>Some discussion around long term goals and home exercise booklet given and programme prescribed (behavioural goals).<br><br>Strength and balance rehabilitation in group or home commences.<br><br>**Trafford:**<br>If recruited through Community Rehabilitation team they receive a 6 week programme. They are then either referred to group-based rehabilitation or discharged. Seen in own home once a week.<br><br>If recruited through the group-based rehabilitation they receive an 8 week group-based programme. Seen in a group once a week.<br><br>They are then referred on to a community based FaME class once a week and prescribed home exercise programme.<br><br>**Manchester City**<br>They receive either a 12 week group based exercise class and prescribed home exercise programme or just a 12 week home based exercise, and

For peer review only

	exercise programme.<br><br>**Manchester City**<br>They receive either a 12 week group based exercise class and prescribed home exercise programme or just a 12 week home based exercise, and then follow-up visits until 6 months (as per patient need). On discharge they are referred to the exercise referral programme where they can attend a group once a week and receive a prescribed home exercise programme. Sometimes they are referred to exercise referral programme before discharge.<br><br>All patients across both sites will receive a prescribed programme by the health professional on discharge and the health professional will set behavioural goals with the patient using the apps. Throughout the rehabilitation period the health professional can upgrade the exercise programme as appropriate using the apps. They will also send the patient a personalised motivational message once a week until discharge.<br><br>Participants receive a visit from the research team the week after their rehabilitation programme has started and then monthly phonecalls.<br><br>Participants can contact the research team for technical support at any time.	then follow-up visits until 6 months (as per patient need). On discharge they are referred to the exercise referral programme where they can attend a group once a week and receive a prescribed home exercise programme. Sometimes they are referred to the exercise referral programme before discharge.<br><br>All patients across both sites will receive a prescribed programme by the health professional on discharge.<br><br>Participants receive a visit from the research team the week after their rehabilitation programme has started and then monthly phonecalls.<br><br>Participants can contact the research team for technical support at any time.
**Item 5: who provided**	**Assessments**<br>All physical assessments completed at the clinical sites are completed by blinded clinical staff from those sites. All assessors are qualified physiotherapists.<br>Assessments are completed at baseline (prior to randomisation), 3 and 6 months. Other assessments are self- completed by the older person.<br><br>**Intervention**<br>Both the intervention and control are delivered by the same health	

For peer review only

	professionals at both sites. These are predominantly Physiotherapists, but also Occupational Therapists and Healthcare Assistants. All health professionals have been trained to deliver the evidence based rehabilitation programme and have also received training on using the smartphone from the research team.		
**6. How**	**Identification**	Patients are identified by health professionals at 2 sites and screened against the inclusion/exclusion criteria. They are then approached by the health professional and asked if they would like a demo of the technology.	
	**Consent**	The researcher contacts the individual and arranges a visit accompanied by a former patient who has used the smartphone app before.<br>The researcher takes consent.	
	**Exercise delivery**	Exercise delivery is the same across intervention and control, but differs dependent on site (see 4.What). Exercise is delivered by the health professional either in the home or within a group.	
	**Motivational input**	**INTERVENTION**	**CONTROL**
		Patient carry out goal setting session with health professional structured by the use of the 'Motivate Me' app and receive a prescribed programme on their app and an exercise booklet.<br><br>Patient will receive prompts and messages through the 'my activity programme app' at home on the days they have planned to exercise.<br><br>Patients will also receive verbal feedback when they see the health professional.	Patient given a prescribed exercise programme and some informal goal setting is carried out as part of the session. They receive a home exercise booklet.<br><br>Patients will only receive verbal feedback when they see the health professional
**7. Where**	The intervention is delivered across several community venues and health centres venues and in patients' homes in Manchester City and Trafford.		
**8. When and how much**		**Intervention arm**	**Control arm**
	Research Team	1 visit to set- up the phone after randomisation	1 visit to set- up the phone after randomisation

For peer review only

		1 visit the week after the rehabilitation programme has commenced<br><br>Phone calls x 6	1 visit the week after the rehabilitation programme has commenced<br><br>Phone calls x 6
	Health Professional	2 x structured goal setting session (baseline and at discharge).<br><br>Weekly face to face exercise intervention (ranging from 6-15 contacts).<br><br>Weekly feedback message received through app until discharge (maximum of 26).	2 x goal setting session (baseline and at discharge).<br><br>Weekly face to face exercise intervention (ranging from 6-15 contacts).
	Smartphone	Automated messages and prompts x 3 on the day they have scheduled to exercise.	
**9. Tailoring**	Rehabilitation programme	The number of home based visits to each patient may differ across both recruitment sites and across both control and intervention groups dependent on patient need.<br><br>The exercise programme delivered will be tailored to each individual patient across both sites and across both control and intervention groups.<br><br>Both sites will send the weekly feedback message until patient discharge (this could be at 8 weeks at Trafford and 6 months at Manchester city).	
	Motivational messages	Because the exercise programme is tailored to the individual, health professionals may schedule for the patient to exercise 3 times a week on the phone or every day, dependent on preference. Participants will receive messages on the days they have scheduled to exercise some patients may receive more than others.	
**11. How well planned**	Health professional fidelity	The research team will attend the goal-setting sessions for the intervention arm for the first 5 patients at each site and then 1 patient at each site every 2 months.<br><br>All staff have undergone a half-day training session and a follow-up support session in using the smartphone app and trial procedures.	

For peer review only

	Assessment fidelity	Assessors masked to group allocation will follow an assessment standard operating procedure.
	Adherence	1. Adherence is collected through the smartphone apps (control and intervention).<br>2. At baseline, 3 and 6 months though validated questionnaire (EARS).<br>3. Through group exercise attendance records, health professional and instructor delivery records.

Item 10 and 12 N/A.

For peer review only

BMJ Open

Can smartphone TechnolOGy be used to support an EffecTive Home ExeRcise intervention to prevent falls amongst community dwelling older adults? The TOGETHER feasibility RCT study protocol.

Journal:	*BMJ Open*
Manuscript ID	bmjopen-2018-028100.R2
Article Type:	Protocol
Date Submitted by the Author:	06-Aug-2019
Complete List of Authors:	Hawley-Hague, Helen; University of Manchester, School of Health Sciences<br>Tacconi, Carlo; Universita degli Studi di Bologna Dipartimento di Ingegneria dell'Energia Elettrica e dell'Informazione Guglielmo Marconi<br>Mellone, Sabato; University of Bologna, Department of Electrical, Electronic and Information Engineering «Guglielmo Marconi»<br>Martinez, Ellen; Central Manchester University Hospitals NHS Foundation Trust<br>Easdon, Angela; Pennine Care NHS Foundation Trust<br>Yang, Fan; University of York, Centre for Health Economics<br>Su, Ting-Li; University of Manchester, Dentistry<br>Mikolaizak, A. Stefanie; Robert Bosch Krankenhaus<br>Chiari, Lorenzo; University of Bologna, Department of Electrical, Electronic and Information Engineering «Guglielmo Marconi»<br>Helbostad, Jorunn; The Norwegian University of Science and Technology, NTNU, Department of Neuromedicine and Movement Science, The Faculty of Medicine and Health Sciences<br>Todd, Chris; University of Manchester, School of Health Sciences
**Primary Subject Heading**:	Rehabilitation medicine
Secondary Subject Heading:	Geriatric medicine, Health services research
Keywords:	REHABILITATION MEDICINE, older, mhealth

SCHOLARONE™
Manuscripts

Can smartphone **T**echnol**OG**y be used to support an **E**ffec**T**ive **H**ome **E**xe**R**cise intervention to prevent falls amongst community dwelling older adults? The **TOGETHER** feasibility RCT study protocol.

Hawley-Hague, H[1]., Tacconi, C[2,3]., Mellone, S[2,3,4]., Martinez, E[5], Easdon, A[6]., Yang, F[7]., Su, T[8]., Mikolaizak, A.S[9]., Chiari, L[2,3,4]., Helbostad, J[10]., Todd, C. [1, 11]

[1] School of Health Sciences, Faculty of Biology, Medicine and Health, The University of Manchester and Manchester Academic Health Sciences Centre, Manchester, U.K.
[2] Health Sciences and Technologies-Interdepartmental Center for Industrial Research, University of Bologna, Bologna, Italy.
[3] mHealth Technologies s.r.l., Bologna, Italy,
[4] Department of Electrical, Electronic and Information Engineering «Guglielmo Marconi» - University of Bologna, Bologna, Italy.
[5] Manchester University NHS Foundation Trust, Manchester, U.K
[6] Pennine Care Hospital Trust, Manchester, U.K
[7] Centre for Health Economics, University of York, York, U.K
[8] School of Medical Sciences, Faculty of Biology, Medicine and Health, The University of Manchester, Manchester, U.K
[9] Robert Bosch Krankenhaus, Department of Clinical Gerontology, Stuttgart, Germany
[10] Department of Neuromedicine and Movement Science, The Faculty of Medicine and Health Sciences, The Norwegian University of Science and Technology, NTNU, Trondheim, Norway
[11] Manchester University NHS Foundation Trust, Manchester, U.K.

Corresponding author:
Dr Helen Hawley-Hague
The University of Manchester
School of Health Sciences (Nursing, Midwifery and Social Work)
Floor 6, Jean McFarlane Building
Oxford Road
Manchester
M13 9PL
Helen.Hawley-Hague@manchester.ac.uk

Word count: 4,649

For peer review only

For peer review only - http://bmjopen.bmj.com/site/about/guidelines.xhtml

ABSTRACT

Introduction

Falls have major implications for quality of life, independence and cost to the health service. Strength and balance training has been found to be effective in reducing the rate/ risk of falls, as long as there is adequate fidelity to the evidence-based programme. Health services are often unable to deliver the evidence-based dose of exercise and older adults do not always sufficiently adhere to their programme to gain full outcomes. Smartphone technology based on behaviour-change theory has been used to support healthy lifestyles, but not falls prevention exercise. This feasibility trial will explore whether smartphone technology can support patients to better adhere to an evidence-based rehabilitation programme and test study procedures/outcome measures.

Methods and analysis

A two-arm, pragmatic feasibility randomised controlled trial will be conducted with health services in Manchester, UK. Seventy-two patients aged 50+ years eligible for a falls rehabilitation exercise programme from two community services will receive: (1) standard service with a smartphone for outcome measurement only or (2) standard service plus a smartphone including the motivational smartphone app. The primary outcome is feasibility of the intervention, study design and procedures. The secondary outcome is to compare standard outcome measures for falls, function and adherence to instrumented versions collected using smartphone. Outcome measures collected include balance, function, falls, strength, fear of falling, health related quality of life, resource use and adherence. Outcomes are measured at baseline, three and six month post-randomisation. Interviews/focus groups with health professionals and participants further explore feasibility of the technology and trial procedures. Primarily analyses will be descriptive.

Ethics and dissemination.

The study protocol is approved by North West Greater Manchester East Research Ethics Committee (Rec ref:18/NW/0457, 9/07/2018). User groups and patient representatives were consulted to inform trial design, and are involved in study recruitment. Results will be reported at conferences and in peer-reviewed publications. A dissemination event will be held in Manchester to present the results of the trial. The protocol adheres to the recommended SPIRIT Checklist.

Trial registration number: ISRCTN12830220

Protocol Version: V1.3 31.7.2019

Key words: Rehabilitation, older, technology

For peer review only - http://bmjopen.bmj.com/site/about/guidelines.xhtml

Strengths and limitations of this study

- The first study to examine a motivational app to support falls rehabilitation.
- Pragmatic feasibility trial enables us to establish whether it is feasible to use the motivational apps in practice.
- Multi-site study with different types of falls services, representative of UK service delivery.
-
- Due to the nature of the intervention we will not be able to blind participants or those delivering the intervention.

For peer review only

For peer review only - http://bmjopen.bmj.com/site/about/guidelines.xhtml

Falls are an important public health issue, with over 30% of people aged 65 and over falling at least once a year[1]. This has implications for quality of life, independence and cost to the health service[1]. Strength and balance training (SBT) comprises 'carrying out exercises that increase muscle strength in the legs and improve balance'[2]. Strength and balance exercise programmes are effective in reducing risk and rate of falls and injuries[3]. Sherrington et al[4] have shown that for strength and balance programmes to be effective they need to be progressive, tailored and of adequate dose (3x a week for 50 hours, and then maintained). Work carried out by Public Health England[5] illustrates that to see a return on investment; fidelity to the evidence-base has to be carried-out (adequate dose, progression).

However, Nyman and Victor[6] report that adherence to evidence-based strength and balance programmes is poor. The National Health Service (NHS) only delivers programmes that are pre-dominantly 3 months or less[7], older adults do not carry out their exercise programme three times a week as prescribed (dose) or carry out the programme for a sufficient length of time to achieve and maintain the benefits[6,7]. Cost and appropriate staffing are cited as primary reasons for short NHS delivery[7].

Unless there are innovative new solutions to support the delivery of falls prevention exercises sufficiently to reduce falls risk and to prevent re-referral to services, over the coming decade it is estimated that population changes will result in service demand beyond the reach of current interventions[8].The use of smartphones to support falls rehabilitation could be one of the solutions. The proportion of older adults using smartphones is growing rapidly, with 39% of those aged 65 to 74 and 15% of those aged over 75 using smartphones[9]. Smartphones offer multiple opportunities to support healthy ageing and falls prevention as they are portable, can be body-worn and can therefore be used for falls detection, movement detection and motivation[10, 11, 12] The evidence which looks at the role of the smartphone for falls prevention is sparse[13], particularly for interventions focused on rehabilitation/strength and balance training. Although, there is a lack of specific evidence related to falls prevention interventions, there is evidence that older adults find mobile phones more usable than using a new device e.g. a falls alarm[14]. It has also been suggested that barriers to smartphone use in this population can be overcome through adequate support and affordability[15]. There is evidence supporting the use of mobile phone-based healthy lifestyle programmes[16,17], including to increase physical activity[17,18,19]. King et al[11], developed and tested smartphone applications (apps) based on behaviour change theory designed to motivate adults aged 45+ years. One of these included personalised goal-setting and behavioural feedback, successful evidence-based behavioural change techniques[20]. The apps recieved positive feedback from participants and increased physical activity.

We know from previous studies that attitudes and beliefs are important to the uptake of and adherence to exercise by older adults[21,22]. The Theory of Planned Behaviour (TPB) [23] is particularly useful for assessing older adults' attitudes in relation to exercise uptake and adherence[21,22,24]. The TPB is based on three core components:
(i) perceived behavioural control (PBC), the perceived ease or difficulty of performing the behaviour.
(ii) social influences including, subjective norms (beliefs of important people e.g. family), perceived social support (support from others for behaviour) and modelling (following observed behaviour of others).

For peer review only - http://bmjopen.bmj.com/site/about/guidelines.xhtml

(iii) attitudes (outcome expectations)[23]. Focused on the advantages and disadvantages of the behaviour (outcome expectations) and when related to adherence, whether these advantages have occurred.

Attitudes measured by using a TPB-based tool have been significantly associated with exercise behaviour in a previous study[21]. This theory has informed the intervention overall and content of the motivational messages within the proposed intervention (focused on outcome expectations/PBC).

Smartphone technology-based motivational applications underpinned by behaviour change theory and developed with health professionals and older adults could be an effective way of encouraging maintenance of exercise and of successfully supporting adherence to evidence-based strength and balance training. We have already carried out usability and acceptability testing of the technology and two motivational apps (one for health professionals and one for patients), before planning this trial. The smartphone apps have been developed through several cycles of user-led design. Initially we carried out engagement workshops with older adults (AgeUK) and health professionals from one falls service in Manchester, followed by usability/acceptability testing with another falls service in Manchester and their patients (IRAS:205980). The use of this approach has enabled us to develop the apps, establish whether the technology is acceptable to older adults and health professionals (qualitative methods) and to check its usability (technology testing). Overall, the apps were acceptable to both patients and health professionals with the majority of suggested changes made to the health professionals' app to ensure it fit more easily with their practice. Changes following this testing included; improvements in the delivery of messages and a more streamline approach to scheduling activities for the health professional. Another suggested change was to make smartphone pens available to participants to aid in the use of the touchscreen.

This study now aims to explore whether it is feasible for smartphone technology to be used to support patients to sufficiently adhere to an evidence-based exercise rehabilitation programme. As a secondary aim it will assess whether technology-based outcome measures (smartphone-based falls alarm and Timed up and Go Test)[25] are reliable when compared to standard methods (e.g. falls calendars). Through a feasibility RCT we will explore the feasibility of using smartphone technology to support falls rehabilitation and test study procedures (e.g. suitability of outcome measures, standard deviation of the outcome measure, recruitment, randomisation, follow-up rates, retention, time required for analysis). Both arms of the trial will receive rehabilitation exercises and will report their exercises on a study provided smartphone but only the intervention arm will carry out goal-setting and receive feedback through the phone.

The intervention has the potential to:
1. Increase the amount of support the patient receives to adhere to their exercise, leading to increased adherence.
2. Increase exercise progression/dose which could be cost neutral/saving.
3. Enable health professionals to monitor compliance to the prescribed programme.
This could assist maintenance of health, reducing long-term falls risk and re-access to services.

For peer review only - http://bmjopen.bmj.com/site/about/guidelines.xhtml

METHODS

Trial design

Core trial information is presented in Table 1. This study is a two-arm pragmatic feasibility randomised controlled trial including the collection of economic data. The trial design framework is exploratory**.** Alongside the trial, qualitative work is carried out to understand the feasibility of the intervention and the trial procedures.

Table 1: WHO Trial Registration Data Set

**Data category**	**Information**
Primary registry and trial identifying number	ISRCTN: 12830220
Date of registration in primary registry	21.08.2018
Secondary identifying numbers	
Source of monetary or material support	National Institute for Health Research Postdoctoral Fellowship Award
Primary sponsor	University of Manchester
Secondary sponsor	N/A
Contact for public queries	Helen.hawley-hague@manchester.ac.uk
Contact for scientific queries	Helen.hawley-hague@manchester.ac.uk
Public title	The Together trial
Scientific Title	Can smartphone **T**echnol**OG**y be used to support an **E**ffec**T**ive **H**ome **E**xe**R**cise intervention to prevent falls amongst community dwelling older people? The **TOGETHER** feasibility RCT.
Countries of recruitment	UK
Health condition of problem studied	Falls in Older Adults
Interventions	**Standard service:** **Manchester City**: 12 weeks once a week contact (home or group exercise), check-ups until 6 months discharge. **Trafford:** 8 weeks group exercise once a week or 6 week home exercise then discharged or referred to further 8 week group exercise.

For peer review only - http://bmjopen.bmj.com/site/about/guidelines.xhtml

	For all prescribed exercise plan and exercise booklet given, asked informally what they want to achieve (outcome goals).<br>Use of study provided smartphone for reporting exercises and falls detection as outcome measures only<br><br>**Intervention:** Standard service plus the use of Motivate Me (health professional app) and My Activity Programme (patient app) on study provided smartphones.
Key inclusion and exclusion criteria	**Age:** Older adults aged 50+<br>**Sex:** Male or female<br>**Inclusion:** At risk of falls, referred to falls rehabilitation services and assessed as suitable for an exercise programme, Good 3G/4G reception in their home or wifi.<br>**Exclusion:** unable to follow instructions (unless they have support from a family member or carer), Severe visual impairment, long-term residential or nursing care, terminal illness or expected shortened lifespan, defined as less than 6 months, Older adults unable to read written English unless they have support from a family member or carer).
Study type	Interventional<br>Allocation: randomised;<br>Primary purpose: prevention, feasibility
Date of first enrolment:	September 2018
Target sample size	72
Recruitment status	Pending
Primary outcome	Feasibility of the design and procedures
Key secondary outcomes	Balance (Berg), Function (TUG/mTUG), Falls (Calendar/FallsMonitor@home), Strength (30 second chair stand), Fear of Falling (Short FES-I), Health related quality of life (EQ5D-5L/ ICE-CAP-O), resource use, adherence (my activity programme/EARS).<br>Baseline, 3, 6 months.

For peer review only

Sampling principles and procedures

Eligibility
Older adults at risk of falls (aged 50+ years) and assessed as requiring a falls rehabilitation exercise programme, are identified through current community falls rehabilitation services delivered in Manchester City and Trafford. The two sites see patients from diverse socio-economic populations. Exclusions include older adults who are unable to follow instructions (unless supported by a family member/carer), who are unable to understand written English (unless supported by a family member/carer), with severe visual impairment, those in long-term residential or nursing care and those with terminal illness or expected shortened lifespan, defined as less than 6 months, as determined by the NHS teams. Patients need to have good 3G/4G mobile phone reception (able to access webpages) or wifi in their home and this is assessed by the health professional before they handout participant information or by the researcher when taking consent.

Recruitment, consent, sample size
Health professionals give patients the study information sheet and inform them about the intervention. The health professionals then ask the patient if they are happy to be contacted by the researcher who demonstrates the technology either in the patient's home or within groups at each NHS site. The technology is demonstrated to the participant before they are asked to give informed consent. Where possible a former patient who has used the smartphone applications accompanies the researcher to demonstrate the technology. We think involvement of a peer has the potential to assist in promoting patient confidence in the use of the technology.

The first 36 eligible patients identified through each service (N=72 in total) who are willing to participate are being recruited and randomised (Figure 1). Thirty patients per arm after attrition (approx. 10%) are normally used for feasibility RCTs[26]. Study participants are randomised using a computer-generated randomisation algorithm at sealedenvelope.com, stratified by gender and site, using block randomisation (2, 4, 6 blocks) into either intervention or control group. Stratification is by gender and site to ensure equal distribution across sites as we are testing all trial procedures.

Blinding

Baseline and follow-up (3 and 6 months) assessments are carried out by experienced clinicians within each NHS Trust (not a member of the clinical teams participating), who are blinded to which intervention the participants are receiving, at baseline the individual is also blinded to the intervention they will receive as randomisation occurs after baseline assessment.

As this is an 'active' intervention, it is not possible to blind the health professionals delivering the service or the participants during the intervention. The lead researcher provides technical support to both arms to use the smartphone so is not blinded.
The statistical analysis will be carried out by the lead researcher with the support of a statistician. Patient ID codes will be removed from the data to allow for blinded analysis.

Patient withdrawal

In consenting to the trial, patients are consenting to the trial treatment, follow-up and data

For peer review only - http://bmjopen.bmj.com/site/about/guidelines.xhtml

collection. If withdrawal of the randomly allocated treatment occurs, patients should still be followed up where they agree. Patients are allowed to withdraw without giving reason at any time and a withdrawal case report form (CRF) will be completed to document the date and reason (where given) for withdrawal. Data collected up to the time of withdrawal will be included in analyses. Health professionals will assess patients' capacity to take part in the rehabilitation programme and the study, if they have been deemed to have lost capacity to consent they will be withdrawn from the study but the data already collected will be retained.

Interviews with patients

All participants are offered an interview (even those who withdraw from the trial) in their own home after the final follow-up to assess their experiences of the intervention and trial processes. Family members/carers may also attend the interview at the participants' request.

Focus groups with health professionals

Health professionals from Trafford and Manchester city who are involved in the study are recruited to participate in a focus group at the end of the study (after 24 weeks follow-up). All members of staff (N=8) will be given study information by their team leader and asked if they are available for a focus group, the focus groups will take part at their place of work at a time convenient to each team. Participating staff can choose to be part of a one-to-one interview if they prefer not to be interviewed with colleagues or if for staffing reasons it is not feasible for them to attend the focus group.

The Intervention

Full details of the intervention components are shown in Supplementary material: Table 1 (TIDieR Guidelines).

The technology

The Samsung Galaxy J5 as a means of communication[27] will be provided to all participants and health professionals. Samsung phones have been used previously in our research, with good usability and have the correct specification for the falls detector to work [10]. The research team will provide technical support for participants and health professionals (HHH) and any required application updates (SM, CT).

'Motivate me' app

The 'Motivate me' app is the health professional application. This app is used by the health professional with the patient to set behavioural and outcome-based goals, for the health professional to see what exercises the patient has reported and to give feedback and to check they have received messages (Supplementary Material Figure 1).

'My activity programme'

'My activity programme' is the patients application. This app will be used by the patient to

For peer review only - http://bmjopen.bmj.com/site/about/guidelines.xhtml

report the exercises they have done, receive messages and prompts and to confirm whether they like the messages received (Supplementary Material Figure 2).

There are 12-behaviour change techniques adopted[28] through the intervention include goal-setting (behaviour/outcome), action-planning (recording plan to exercise in diary on smartphone/reminder text messages when it is time to start the programme), and feedback on behaviour (providing feedback on what they have done/benefits). The key behavioural change techniques delivered as part of the control and intervention arms are outlined in Table 2.

Table 2: Behaviour change techniques adopted*

	**1.Intervention arm**	**1a How**	**2.Control arm (standard service)**	**2a How**
1.1 Goal setting (behaviour)	x	what, when, where- smartphone and paper	x	What, Where- Paper
1.3 Goal setting (outcome)	x	smartphone verbally	x	Verbally
1.4 Action planning	x	smartphone		
1.5 Review Behavioural goals	x	smartphone verbally	x	Paper Verbally
1.7 Review outcome goals	x	smartphone verbally	x	Verbally
2.2 Feedback on behaviour	x	Smartphone verbally	x	Verbally
4.1 Instructions on how to perform the behaviour	x	Physically<br>Smartphone Paper	x	Physically<br>Paper
5.1 Information about health consequences	x	Smartphone Verbally (ad hoc)	x	Verbally (ad hoc)
5.6 Emotional Consequences	x	Smartphones Verbally (ad hoc)	x	Verbally (ad hoc)

For peer review only

6.1 Demonstration of behaviour	x	Physically	x	Physically
7.1 Prompts	x	Smartphone		
8.7 Graded tasks	x	Smartphone Paper	x	Paper

*Based on Michie et al[28] behaviour change taxonomy

The Control

Standard service is variable across different sites, but all sites deliver a mix of the evidence-based Falls Management Programme (FaME) and Otago[29] exercises as standard care.

They include face-to-face delivery once-a-week and a prescribed home-exercise programme (with booklet) with informal outcome-based goal-setting.

Manchester City: Once a week visits (either home-based or group exercise) for around 12 weeks (dependent on need) and then check-ups until 6 months discharge.

Trafford: 8 weeks group exercise once a week then discharged, or 6 week home-based exercise then discharged or referred to further 8 week group exercise.

Both sites leave participants with a home exercise plan on discharge and where appropriate refer onto community-based strength and balance programmes.

Control application for self-reporting exercise

The control arm receives a study phone with a basic app where they report their exercises, but they are only able to report their exercises (outcome measure), they are not able to view their programme, receive messages or receive feedback on the phone. The health professional is not able to view what they have reported (outcome measure for the research team only), thereby minimising risk of contamination.

Co-treatments

Trial participants are free to seek management of falls and other related or unrelated medical conditions during the course of the trial. We record all health service resource use and these will be reported as a trial outcome. At trial closure, participants will continue with usual healthcare, no further ancillary care is provided beyond that immediately required for the proper and safe conduct of the trial.

Outcome measures

Data such as demographics (age, gender, socio-economics, health conditions, falls history, previous smartphone/mobile phone use and wifi) and physical tests are recorded on the CRF (Table 3).

For peer review only - http://bmjopen.bmj.com/site/about/guidelines.xhtml

Table 3: Schedule of enrolment interventions and assessments

	STUDY PERIOD					
	Enrolment	Allocation	Post-allocation			Post intervention
**TIMEPOINT****	**$-t_1$**	**0**	**T_1**	**T_2**	**T_3**	
**ENROLMENT:**						
**Eligibility screen**	X					
**Consent to further information**	X					
**Tech demo and Informed consent**	X					
**Allocation**		X				
**INTERVENTIONS:**						
Control:						
CFS			◆———	———	———◆	
TCS			◆———◆			
Intervention			◆———	———	———◆	
**ASSESSMENTS:**						
**Gender<br>Age<br>Ethnicity<br>Education<br>Housing<br>Falls history<br>Medical history<br>Previous mobile/smartphone use<br>Allocated to home or group exercise**	X					
Falls (Calendar)<br>Falls (alarm)<br>My activity self-report<br>Prescribed exercise plan<br>Face to Face delivery			◆———	———	———◆	
Berg<br>TUG<br>mTUG<br>30 Second Chair Stand<br>FES-1<br>EQ5D<br>Resource Use<br>Health professional time resource<br>ICE-CAP-O	X			X	X	

For peer review only - http://bmjopen.bmj.com/site/about/guidelines.xhtml

EARS						
Interviews Focus groups						X

Primary outcome measures

Assess feasibility and acceptability of the design and procedures including:

a. willingness of participants to be randomised (collected on the screening section of the CRF/interviews)
b. willingness of clinicians to recruit participants (through focus group/completion of screening on the CRF)
c. number of eligible patients (through the CRF and documentation of number of monthly referrals to falls services)
d. whether demonstration by peer of the technology aids recruitment.
e. characteristics of the proposed outcome measures e.g. reliability of falls detector when compared to falls calendars, whether a self-report app is a reliable outcome measure.
f. follow-up rates, adherence/compliance rates
g. time needed to collect and analyse data
h. determine effect sizes for use in sample-size calculations, enabling power calculations for the reduction in falls for a definitive large scale RCT.

Start/stop criteria for going to full trial are included in CONSORT diagram (Diagram 1) based on these outcome measures.

We will also report intervention fidelity, process and compliance using observation during quality assurance visits. Health professionals and the assessors will follow a Standard Operating Procedure (SOP) for assessment and intervention. The trial treatment record (CRF) includes details of the grade and type of staff involved with delivery. The health professionals and researcher will keep an issue-log of technical issues with the phones or apps during the trial either experienced directly or reported by participants. We will also explore the potential impact of differing length of exercise delivery across sites.

For peer review only

Outcome measures

Falls

The primary outcome for any future definitive trial would be falls, expressed as fall rate per person per months of follow-up observation after randomisation. The current study collects falls data for the purposes of testing feasibility of data collection, and to inform us of falls rates and intervention effect size for a future sample size calculation.

All participants will wear the smartphone in their pocket or on a waistband and this will act as a falls detector, running the FallsMonitor@home app developed by the University of Bologna[10]. If the patient chooses then they can also use the app on the phone as a falls alarm. The fall detection system application allows the user to identify a list of formal/informal caregivers who will receive an SMS if a fall is detected. Patients are given

For peer review only - http://bmjopen.bmj.com/site/about/guidelines.xhtml

an opportunity to de-active the falls alarm through an application on the smartphone if there is a false alarm, enabling the user to maintain control and prevent unwanted intrusion. Participants are asked if we can use their anonymised falls data for further development of the app and in the Farseeing real-world falls database[30].

To validate this as an outcome measure we use the internationally agreed ProFaNE falls definition[31] and follow the agreed ProFaNE falls data collection and analysis protocols based on self-report calendars[32].

Fear of falling
Short Falls Efficacy Scale-International (Short FES-1) is used to measure fear of falling[33]. This is often a measure used by UK falls services as part of standard outcome measures.

Function
The Timed Up and Go test (TUG) will be used to assess improvements in mobility and function. The TUG will be applied as described by Podsiadlo and Richardson[25]. Participants will be asked to perform the TUG at their self-selected habitual walking speed. A medical device implementing an instrumented version of the TUG will be used (mTUG, mHealth Technologies s.r.l., Bologna, Italy). The device is able to automatically provide guidance to the user for administering the test, capture and process the data, and generates summary reports of function for the health professional. The blinded assessor will complete the normal TUG and the mTUG as outcome measures (the standard TUG as a validation measure) to explore whether the mTUG is usable as an outcome measurement for the definitive RCT. The health professional will carry out the mTUG with a sub-sample of 10 patients at each site to assess their experiences of its use.

Balance
The Berg Balance Scale will be used to assess balance. This has good validity and sensitivity in this population[34] and is one of the best outcome measures for assessing standing balance[35]. It has also been used for the prediction of falls[36]. The effect sizes from this outcome measure scale will be used as part of the power calculation for the full trial.

Strength
30 seconds chair stand test[37], which has good validity and is used throughout health services will be used to assess physical ability, in particular strength.

Adherence
Adherence will be measured in a number of ways (outlined in detail, Table 4):
1) Self-report app will be used for both control and intervention group. Adherence will be classed as the participant carrying out 80% of their prescribed programme (based on the evidence-base for effective strength and balance)[6,38].
2) Exercise Adherence Rating Scale (EARS)[39]. This is a validated 16-question tool with a 6-question subscale specifically measuring adherence (remaining questions measure reasons for adherence/non-adherence).

Health economics
The health related quality of life measures will include the European Quality of Life 5

For peer review only - http://bmjopen.bmj.com/site/about/guidelines.xhtml

Dimensions (EQ-5D-5L)[40] and an additional measure used in previous trials related to falls prevention (the ICEpop CAPability measure for Older people (ICE-CAP-O)[41,42]. Costs of delivering the intervention will be observed based on staff training, delivery costs and equipment costs. Additional resource use measures will be captured via a Resource Use Questionnaire which will seek to measure costs related to an NHS and social care perspective (secondary, primary, community care service use), and a patient perspective (costs related to informal care). The findings from these will inform the feasibility of collection of the data, and priorities for cost collection at full trial.

For peer review only

For peer review only - http://bmjopen.bmj.com/site/about/guidelines.xhtml

Table 4: Adherence measures

	What	How/additional validation
**Self-report through my activity programme and control arm smartphone app**	Exercises reported on app to their prescribed programme they day they are carried out.<br>- Exercise Type<br>- Intensity<br>- Dose<br>Adherence defined as participant carrying out 80% of their prescribed programme.	The health professional will be asked to provide a copy of the participants prescribed exercise plan, any changes to it and the dates any changes were made (both sites record this as part of standard intervention).<br><br>For face to face home delivery, the health professional will be asked to report exactly what the patient has done when with them (this will be used to validate the self-report from participants).<br><br>After discharge from rehabilitation if participants move onto other strength and balance provision. Those services will give us copies of the exercise programme delivered for any days the participants attend, attendance records and any prescribed home exercise programme.
**EARS**	Validated 16-question tool with a 6-question subscale specifically measuring adherence.	Paper questionnaire at baseline, 3 months and 6 months.

Interviews/focus groups

The interview and focus group schedules are based on FARSEEING guidelines[43]. The following key areas will be explored in relation to the smartphone, the 'Motivate me' app, 'My activity programme' app, FallsMonitor@home, and the mTUG. Ease of use, clarity of screen, demonstration of use, wearing comfort, adaption of use, reliability, choice and control and home and lifestyle. This feedback will be considered and any required changes to the technology set-up, applications and intervention will be made prior to the definitive RCT. We also ask additional questions about the research process including: general expectations and views; experiences of recruiting patients (health professional), and of being recruited and randomised (patients), suggestions of methods for recruiting participants; likely uptake and retention of participants.

For peer review only - http://bmjopen.bmj.com/site/about/guidelines.xhtml

Analysis

Quantitative data is analysed using SPSS Release 22.0. The main analyses is descriptive, involving the estimation of recruitment rates, attrition rates, non-compliance rates, means and standard deviations of outcomes by group at baseline and end trial, and 95% confidence intervals for differences of means of outcomes between groups and assessment of change following the intervention at end trial. The health economics analysis is focussed on informing relevant measures and means of collection of health related quality of life and resource use for the future definitive study. Only an exploratory cost-effectiveness analysis will be conducted, for all measures we will report mean values and sample variability alongside information on missing values.

Data from the smartphone based outcome measures (FallsMonitor@home, mTUG, My activity programme/control self-report app) will be compared to the traditional measures (falls calendar[32], TUG[25], EARS[39]) alongside qualitative feedback as part of their validation. A statistical analysis plan will be created before data analysis.

Qualitative interviews/focus groups will be analysed using thematic analysis[44]. The research will be inductive and although will seek to further understand the quantitative findings, this approach will also generate categories and explanations directly from the data rather than based on previously set aims and objectives, reducing risk of bias[44]. QSR International's NVivo 10 qualitative data analysis software will be used to manage the data. The validity of the analysis will be checked by returning to the data once themes have been identified and also through the use of a second researcher who will check samples of analysis. The accuracy of the transcripts will be checked through discussion with participants to establish if anything is not clear from the interviews/focus groups.

Ethical issues

Ethical approval has been granted from the North West Greater Manchester East Research Ethics Committee (Rec ref: 18/NW/0457, 9/07/2018). Regional and site-specific approvals have been obtained. We are collecting and storing personal information in accordance with the General Data Protection Regulation (GDPR) and Data Protection Act 2018. As this is a study with older patients a number of ethical issues could arise. To address these, community services will act as gatekeepers to access patients and assess patients' eligibility for the study. The intervention is delivered by health service staff and provided in addition to standard service, therefore patients are unlikely to be disadvantaged.

If falls are detected by the smartphone, it is important that someone is informed in real-time. The smartphone application allows the user to select a list of formal/informal caregivers who will receive an SMS if a fall is detected. It will be made clear that the falls service is not an emergency service so in the event of a fall the person receiving the text message would call an ambulance as they would in normal circumstances. If patients already wear a call alarm then they will be encouraged to continue to use this as well or to adopt their usual method of alerting help.

The study requires monitoring of subjects and it is important that patients do not find this obtrusive (privacy issues have been identified as major barriers to the use of technology). Patients are given an opportunity to de-active the falls alarm through an application on the

For peer review only - http://bmjopen.bmj.com/site/about/guidelines.xhtml

smartphone if there is a false alarm. However, previous consultation/usability testing with older adults raised no major privacy issues.

There are ethical issues in the removal of technology at the end of studies[45]. We will not be able to offer older adults the technology at the end of the 6 month study period, but they will be offered the opportunity to download the apps onto their own phones if they wish.

The risk of interviews and focus groups are minimal. The patient or health professional can ask the researcher to move onto another question if they are uncomfortable at any point. Health professionals will be given the chance to discuss the trial, technology and intervention in a one-to-one interview if they do not feel comfortable giving feedback in front of colleagues.

Patient and public involvement

Patient and public representatives have been involved in designing the trial including outcome measures. Feedback from previous usability testing with patients and from patients who sit on our Advisory Group (AG) provided direct information on the design of the trial e.g. use of self-report app for control arm. Patients on our AG (who were formerly patients of one of the services) helped to design study material such as the patient information sheet. They assisted in training health professionals in approaching patients for recruitment and goal-setting as part of the intervention. Three participants', who took part in our usability testing, became peer mentor volunteers for the trial. They will attend the first visit (if the patient gives permission) to demonstrate the technology to patients before consent is given. We will explore whether peer involvement aids recruitment. Finally, the volunteers and the patients who sit on our AG will aid with dissemination of study findings e.g. helping to arrange dissemination events and providing feedback on newsletters for participants.

Trial monitoring

The lead researcher (HHH) will monitor the delivery of the intervention and recruitment of patients, there will also be a clinical lead (AE, EM) at each site taking overall responsibility for identification of patients and delivery of the intervention. This team, alongside academic experts (JH, LC, SM, ASM, CT) from the Trial Co-ordination Group will ensure overall quality of trial data. The AG, which meets bi-annually, giving feedback on the project, providing expert guidance and assisting in dissemination, this includes two previous patients. A risk register is reviewed by the AG. The study is subject to the audit and monitoring regime of the University of Manchester and a monitoring plan followed.

A detailed risk assessment has been carried out and potential patient, organisational and study hazards considered, the likelihood of their occurrence and the resulting impact should they occur.

Adverse events

A safety reporting protocol has been developed for related and unexpected serious adverse events (AEs) and directly attributable AEs. An AE is defined as any untoward medical occurrence in a subject which does not necessarily have a causal relationship with treatment. These AEs are recorded in the CRF and if a Serious Adverse Event (SAE)

For peer review only - http://bmjopen.bmj.com/site/about/guidelines.xhtml

occurs then reported to the Chief investigator. The CI will determine whether AEs require reporting to the trial sponsor and Ethics Committee, in accordance with the safety reporting protocol.

DISCUSSION

This is the first trial that we are aware of that explores the potential use of motivational smartphone apps for the support of an evidence-based falls exercise programme.

As this is an active intervention and control we are unable to blind participants or those delivering the intervention. However, the design does enable us to blind both those carrying out the assessments and analysis. The fact that both arms have a smartphone minimises the risk of unblinding with the independent assessors and, we would argue, also reduces risk of drop-out. There is the potential for the control group to become motivated by reporting their activities. However, if we did not ask them to report, there is also the risk of any difference between groups being a function of differential reporting schedules rather than a function of the intervention per se.

We provide participants with study phones, which may be different to using the app on their own phones. However, we need to ensure the smartphone meets the technical specification required for FallsMonitor@home to work correctly. Furthermore use of study phones enables us to maintain confidentiality of participants (if phones are lost we can wipe them remotely).

This trial assesses several novel outcome measures against the gold standard, the mTUG against standard TUG, the FallsMonitor@home against standard calendar method and a self-report app against the EARS tool[39]. This enables us to further our understanding of whether technology has the potential to provide more objective and reliable outcome measures than current methods.

We use two very different NHS sites, reflecting the reality of day-to-day practice (one specialist falls service, one general rehabilitation services) to explore the delivery of the intervention. This means that the standard service is different across the two sites adding complexity to how the control and intervention arm are delivered. However, these differences enable us to assess its scalability to full trial where different types of falls services would need to be included as sites. It also enables us to be more representative of current services and assess its potential for delivery in practice.

Figure 1: Consort diagram

Supplementary Material Figure 1: Motivate Me user interface

Supplementary Material Figure 2: My Activity Programme interface

For peer review only - http://bmjopen.bmj.com/site/about/guidelines.xhtml

Authors' contributions:

HHH leads the research project and its design, managing the trial overall and has led the writing of the protocol. CTa and SM give technical support for the study and have advised on outcomes and the manuscript. JH, LC, ASM, CTo and SM have provided scientific advice around the design of the study and commented on the manuscript. ST and YF have given advice on statistics and health economic part of design and manuscript. AE and EM have given advice on the operationalisation of the study and commented on the manuscript.

Acknowledgments

We thank Professor Dawn Skelton and Later Life Training for allowing us to use their images and the name 'Motivate Me' for the health professional app. We thank two PPI representatives who sit on our Advisory Group and three peer volunteers who are supporting recruitment.

Competing Interests

No competing interests declared.

Funding

Dr Helen Hawley-Hague, is funded by a National Institute for Health Research (NIHR), (Postdoctoral Research Fellow) for this research project.

This publication presents independent research funded by the National Institute for Health Research (NIHR). The views expressed are those of the author(s) and not necessarily those of the NHS, the NIHR or the Department of Health and Social Care.

only

REFERENCES

1. Public Health England. Muscle and bone strengthening and balance activities for general health benefits in adults and older adults. Summary of a rapid evidence review for the UK Chief Medical Officers' update of the physical activity guidelines. London 2018. https://assets.publishing.service.gov.uk/government/uploads/system/uploads/attachment_data/file/721874/MBSBA_evidence_review.pdf (accessed 15 November 2018)
2. Yardley, L., Kirby, S., Ben-Shlomo, Y, et al. How likely are older people to take up different falls prevention activities? *Prev Med*2008;47:554-58. doi: 10.1016/j.ypmed.2008.09.001
3. Gillespie, L.D., Robertson, M.C., Gillespie, W.J, et al. Interventions for preventing falls in older people living in the community. *Cochrane Database of Systematic Reviews.* 9, 2012 Art. No.: CD007146. doi: 10.1002/14651858.CD007146.pub3.
4. Sherrington, C., Tiedemann, A., Fairhall N, et al. Exercise to prevent falls in older adults: an updated systematic review and meta-analysis. *Br J Sports Med*2018;51:1750-1758. doi: 10.1136/bjsports-2016-096547
5. Public Health England (2018) An ROI Tool: Assessment of Falls Prevention Programmes for Older People Living in the Community. Crown Copyright. https://assets.publishing.service.gov.uk/government/uploads/system/uploads/attachment_data/file/679856/A_return_on_investment_tool_for_falls_prevention_programmes.pdf (accessed 6 August 2018)
6. Nyman, S.R. Victor, C.R. Older people's participation in and engagement with falls prevention interventions in community settings: an augment to the Cochrane systematic review. *Age Ageing*2011;41:16-23. doi: 10.1093/ageing/afr103.
7. Royal College of Physicians. Older people's experiences of therapeutic exercise as part of a falls prevention service-patient and public involvement. London 2012: RCP.
8. Rapp, K., Büchele,G., Dreinhöfer, k, et al. Epidemiology of hip fractures. *Z Gerontol Geriatr*, 2018: 1-7. https://doi.org/10.1007/s00391-018-1382-z.
9. Ofcom. Rise of the social seniors revealed. https://www.ofcom.org.uk/about-ofcom/latest/media/media-releases/2017/rise-social-seniors (accessed 12 June 2019).
10. Mellone, S., Tacconi., C, Schwickert., L. Smartphone-based solutions for fall detection and prevention: the FARSEEING approach. *Z Gerontol Geriatr*2012;45:722-7. doi: 10.1007/s00391-012-0404-5.
11. King, A.C., Hekler, E.B., Grieco, L.A, et al. Harnessing Different Motivational Frames via Mobile Phones to Promote Daily Physical Activity and Reduce Sedentary Behavior in Aging Adults. *PlosOne*2013;8. DOI: 10.1371/journal.pone.0062613
12. del Rosario, MB., Redmond, SJ., Lovell, N,H. Tracking the Evolution of Smartphone Sensing for Monitoring Human Movement. *Sensors (Basel)*. 2015. 15: 18901–18933. doi: 10.3390/s150818901
13. Hamm, J., Money, AG., Atwal, A, et al. Fall prevention intervention technologies: a conceptual framework and survey of the state of the art *J. Biomed. Inform*2016;59:319-345. https://doi.org/10.1016/j.jbi.2015.12.013
14. CONFIDENCE (2011) D8.5: Final report. http://www.confidence-eu.org/ (accessed 8 August 2014)
15. Parker, S.J., Jessel, S., Richardson, J.E., Reid, C.M. Older adults are mobile too! Identifying the barriers and facilitators to older adults' use of mHealth for pain management. *BMC Geriatrics* 2013, 13:43. https://doi.org/10.1186/1471-2318-13-43.
16. Fukuoka, Y., Kamitani, E., Bonnet, K, et al. Real-Time Social Support Through a Mobile Virtual Community to Improve Healthy Behavior in Overweight and Sedentary

For peer review only - http://bmjopen.bmj.com/site/about/guidelines.xhtml

Adults: A Focus Group Analysis. *J Med Internet Res*2011;13:e49.
17. Vankipuram, M., McMahon., S., Fleury, J. ReadySteady: App for Accelerometer-based Activity Monitoring and Wellness-Motivation Feedback System for Older Adults. *AMIA Annual Symposium Proceedings*2012:931–939.
18. Worringham C, Rojek A, Stewart I, et al.Development and Feasibility of a Smartphone. ECG and GPS Based System for Remotely Monitoring Exercise in Cardiac Rehabilitation. *PloS one*2011:6. doi: e14669-10.1371/journal.pone.0014669
19. Rabin, C. Bock, B. Desired features of smartphone applications promoting physical activity. *Telemed J E Health*2011;17:1-3. doi: 10.1089/tmj.2011.0055
20. Epton, T., Currie, S., Armitage, C. J. Unique effects of setting goals on behavior change: Systematic review and meta-analysis. *J Consult Clin Psychol*2017;85:1182-98. doi: 10.1037/ccp0000260
21. Hawley-Hague, H., Horne, M., Campbell, M, et al. Multiple levels of influence on older adults' attendance and adherence to community exercise classes. *Gerontologist*2014; 54:599-610. doi: 10.1093/geront/gnt075
22. Yardley, L., Donovan-Hall, M., Francis, C, et al. Attitudes and beliefs that predict older people's intention to undertake strength and balance training. *J Gerontol B Psychol Sci Soc Sci 2007*;2:199-225. doi: 10.1093/geronb/62.2.P119
23. Ajzen, I. *Attitudes, Personality and Behaviour*. Maidenhead, UK: Open University Press 1988.
24. Lucidi, F., Grano, C., Barbaranelli, C, et al. Social-cognitive determinants of physical activity attendance in older adults. *J Aging Phys Act2006;*14:344-59.
25. Podsiadlo, D., Richardson, S. The timed "Up & Go": a test of basic functional mobility for frail elderly persons. *J Am Geriatr Soc*1991;39:142–48.
26. Lancaster, GA. Dodd, S., Williamson, P.R. Design and analysis of pilot studies: recommendations for good practice. *J Eval Clin Pract* 2004; 10:307-12. doi:10.1111/j.2002.384.doc.x
27. Boulton, E. Hawley-Hague, H., Vereijken, B, et al. Developing the FARSEEING Taxonomy of Technologies: Classification and Description of Technology Use (including ICT) in Falls Prevention Studies. *J Biomed Inform* 2016; 61:132-140. doi: 10.1016/j.jbi.2016.03.017
28. Michie, S., Richardson, M., Johnston, M, et al. The behavior change technique taxonomy (v1) of 93 hierarchically clustered techniques: building an international consensus for the reporting of behavior change interventions. *Ann Behav Med*2013;46:81-95. doi: 10.1007/s12160-013-9486-6
29. ProFouND. Strength and Balance Home Exercise Booklet for Older People (English) 2013. http://profound.eu.com/strength-and-balance-home-exercise-booklet-for-older-people-english/ (accessed 8 November 2018)
http://profound.eu.com/otago-exercise-program/#
30. FARSEEING. The FARSEEING real world falls repository, http://farseeingresearch.eu/the-farseeing-real-world-fall-repository-a-large-scale-collaborative-database-to-collect-and-share-sensor-signals-from-real-world-falls/ (accessed 5 August 2018)
31. Lamb S, Jørstad-Stein, EC, Hauer K, et al. Development of a common outcome data set for fall injury prevention trials: the Prevention of Falls Network. Europe consensus *JAGS*,2005;53:1618–1622 doi:10.1111/j.1532-5415.2005.53455
32. Hauer K, Lamb SE, Jorstad EC, et al. Systematic review of definitions and methods of measuring in randomized controlled fall prevention trials. *Age Ageing,*2006;35:5-10. doi: 10.1093/ageing/afi218

For peer review only - http://bmjopen.bmj.com/site/about/guidelines.xhtml

33. Kempen G, Yardley, L Van Haastregt, J.C.M, et al. The Short FES-I: a shortened version of the Falls Efficacy Scale-International to assess fear of falling. *Age Ageing*.2008;37:45-50 doi: https://doi.org/10.1093/ageing/afm157
34. Berg K,O., Wood-Dauphinée, SL., Williams, JI, et al. Measuring balance in the elderly: validation of an instrument. *Can J Public Health*1992; S2:S7–S11.
35. Sibley KM, Howe T, Lamb SE, et al. Recommendations for a Core Outcome Set for Measuring Standing Balance in Adult Populations: A Consensus-Based Approach. *Plos1*2015. https://doi.org/10.1371/journal.pone.0120568
36. Lajoie, Y., Gallagher, S.P. Predicting falls within the elderly community: comparison of postural sway, reaction time, the Berg Balance Scale and the Activities-specific Balance Confidence (ABC) scale for comparing fallers and non-fallers. *Arch Gerontol Geriatr*2004;38:11-26
37. Jones CJ, Rikli RE, Beam WC. A 30-s chair-stand test as a measure of lower body strength in community-residing older adults. *Res Q Exerc Sport*1999;70:113–19. doi: 10.1080/02701367.1999.10608028.
38. Osho O, Owoeye O, Armijo-Olivo S. Adherence and Attrition in Fall Prevention Exercise Programs for Community-Dwelling Older Adults: A Systematic Review and Meta-Analysis. *J Aging Phys Act* 2017. 3:1-34. doi: 10.1123/japa.2016-0326
39. Newman-Beinart, N., Norton, S., Dowling, D, et al. The development and initial psychometric evaluation of a measure assessing adherence to prescribed exercise: the Exercise Adherence Rating Scale (EARS). *Physiotherapy*2017;103:180-85. doi: 10.1016/j.physio.2016.11.001
40. Janssen MF, Pickard AS, Golicki D, et al. Measurement properties of the EQ-5D-5L compared to the EQ-5D-3L across eight patient groups: a multi-country study. *Qual Life Res* 2013;22:1717-1727. doi: 10.1007/s11136-012-0322-4
41. Grewal I, Lewis J, Flynn T, et al. Developing attributes for a generic quality of life measure for older people: Preferences or capabilities? *Soc Sci Med*. 2006;62:1891-1901
42. Davis J, Liu-Ambrose T, Richardson C, et al. A comparison of the ICECAP-O with EQ-5D in falls prevention clinical setting: are they complements or substitutes? *Qual life Res* 2012;22:969-77. doi: 10.1007/s11136-012-0225-4
43. FARSEEING**.** Preliminary guidelines for design and implementation of technologies 2014. http://farseeingresearch.eu/wp-content/uploads/2014/07/Preliminary-Guidelines-V1.0-Public.pdf (accessed 8 August 2017)
44.Braun V. Clarke V. Using thematic analysis in psychology. *Qualitative Research in Psychology* 2006;3:77-101. doi.org/10.1191/1478088706qp063oa
45. Magnusson, L., Hanson, E.J. Ethical issues arising from a research, technology and development project to support frail older people and their family carers at home. *Health Soc Care Community* 2003;11:431-9.

For peer review only - http://bmjopen.bmj.com/site/about/guidelines.xhtml

For peer review only

For peer review only - http://bmjopen.bmj.com/site/about/guidelines.xhtml

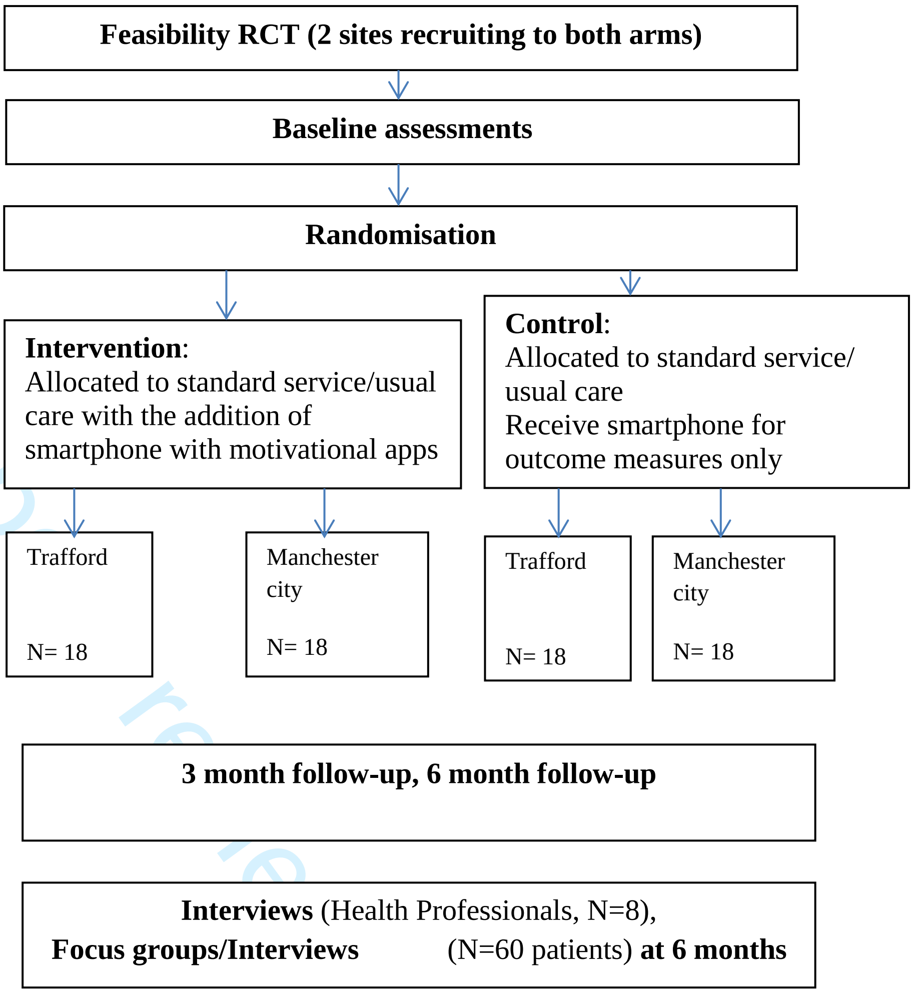

Feasibility RCT

Criteria to judge the feasibility of progression to a definitive RCT based on:

1. ≥30% of eligible patients willing to be recruited to feasibility study;
2. ≥80% of patients complete the intervention
3. Data collected on key outcomes at 6-months follow-up for ≥70% of participants;
4. <10% of Serious Adverse Events deemed due to the intervention itself.

For peer review only

Supplementary Material Table 1: Intervention description using the Template for Intervention Description and Replication (TIDieR) checklist*

<table>
<tr><td>Item 1: Name</td><td colspan="2">Can smartphone TechnolOGy be used to support an EffecTive Home ExeRcise intervention to prevent falls amongst community dwelling older people: The TOGETHER trial</td></tr>
<tr><td>Item 2: Why</td><td colspan="2">Strength and balance training has been found to be effective in reducing the rate and risk of falls. Health services are often unable to deliver the evidence-based dose of exercise and older adults do not always sufficiently adhere to their programme to gain full outcomes. This feasibility trial will explore whether smartphone technology based on the Theory of Planned Behaviour and using goal setting, feedback and prompts can be used to support patients to better adhere to an evidence-based exercise rehabilitation programme and test study procedures and outcome measures.</td></tr>
<tr><td rowspan="2">Item 3: What (Materials)</td><td>Intervention arm: Behaviour change apps</td><td>Control arm:
Standard service</td></tr>
<tr><td>Samsung Galaxy J5

Health Professional phone based Motivate Me app: -
1. set patients long-term goals (outcomes)
2. set patients behavioural goals (which evidence based exercises they will do and how often, when they will exercise with the health professional, alone or in a group),
3. access the patients self-report data and see what exercises they have been doing and when.
4. upgrade exercise programme.
5. give the patient bespoke feedback (set as once a week).

Patient phone based My Activity Programme app:-
1. report the exercises they have done (exercise type, duration, intensity)
2. receive prompts when they have scheduled to exercise
3. receive automated motivational messages based on the long-term goals they have set (with a focus on strengthening outcome</td><td>Samsung Galaxy J5

Control group self-report app:- report the exercises they have done (exercise type, duration, intensity)

Home exercise booklet

Calander/FallsMonitor@home

Patient 'How to guide'

Technology issue log (Health professional)</td></tr>
</table>

For peer review only

	expectations)<br>4. Receive bespoke feedback from health professionals (aimed at increasing Perceived Behavioural Control).<br><br>Home exercise booklet<br><br>Calander/FallsMonitor@home<br><br>Patient 'How to guide'<br>Health professional 'How to guide' (for technology)<br><br>Technology issue log (Health professional)	
**4. What procedure**	Screening and assessment as part of standard service by Falls Services<br><br>Support and training on how to use the 'My Activity Programme app' and falls alarm.<br><br>Formal goal setting session with health professional where use their 'Motivate me' app to set patients' behavioural and outcome-based goals (including prescribing the exercise programme). Handing out of home exercise booklet.<br><br>Strength and balance rehabilitation in group or home commences.<br><br>**Trafford:**<br>If recruited through Community Rehabilitation team they receive a 6- week programme. They are then either referred to group-based rehabilitation or discharged. Seen in own home once a week.<br><br>If recruited through group-based rehabilitation they receive an 8 week group-based programme. Seen in a group once a week.<br><br>They are then referred on to a community based FaME class once a week and prescribed home	Screening and assessment as part of standard service by Falls Services.<br><br>Some discussion around long term goals and home exercise booklet given and programme prescribed (behavioural goals).<br><br>Strength and balance rehabilitation in group or home commences.<br><br>**Trafford:**<br>If recruited through Community Rehabilitation team they receive a 6 week programme. They are then either referred to group-based rehabilitation or discharged. Seen in own home once a week.<br><br>If recruited through the group-based rehabilitation they receive an 8 week group-based programme. Seen in a group once a week.<br><br>They are then referred on to a community based FaME class once a week and prescribed home exercise programme.<br><br>**Manchester City**<br>They receive either a 12 week group based exercise class and prescribed home exercise programme or just a 12 week home based exercise, and

For peer review only

	exercise programme.<br><br>**Manchester City**<br>They receive either a 12 week group based exercise class and prescribed home exercise programme or just a 12 week home based exercise, and then follow-up visits until 6 months (as per patient need). On discharge they are referred to the exercise referral programme where they can attend a group once a week and receive a prescribed home exercise programme. Sometimes they are referred to exercise referral programme before discharge.<br><br>All patients across both sites will receive a prescribed programme by the health professional on discharge and the health professional will set behavioural goals with the patient using the apps. Throughout the rehabilitation period the health professional can upgrade the exercise programme as appropriate using the apps. They will also send the patient a personalised motivational message once a week until discharge.<br><br>Participants receive a visit from the research team the week after their rehabilitation programme has started and then monthly phonecalls.<br><br>Participants can contact the research team for technical support at any time.	then follow-up visits until 6 months (as per patient need). On discharge they are referred to the exercise referral programme where they can attend a group once a week and receive a prescribed home exercise programme. Sometimes they are referred to the exercise referral programme before discharge.<br><br>All patients across both sites will receive a prescribed programme by the health professional on discharge.<br><br>Participants receive a visit from the research team the week after their rehabilitation programme has started and then monthly phonecalls.<br><br>Participants can contact the research team for technical support at any time.
**Item 5: who provided**	**Assessments**<br>All physical assessments completed at the clinical sites are completed by blinded clinical staff from those sites. All assessors are qualified physiotherapists.<br>Assessments are completed at baseline (prior to randomisation), 3 and 6 months. Other assessments are self- completed by the older person.<br><br>**Intervention**<br>Both the intervention and control are delivered by the same health	

For peer review only

	professionals at both sites. These are predominantly Physiotherapists, but also Occupational Therapists and Healthcare Assistants. All health professionals have been trained to deliver the evidence based rehabilitation programme and have also received training on using the smartphone from the research team.		
**6. How**	**Identification**	Patients are identified by health professionals at 2 sites and screened against the inclusion/exclusion criteria. They are then approached by the health professional and asked if they would like a demo of the technology.	
	**Consent**	The researcher contacts the individual and arranges a visit accompanied by a former patient who has used the smartphone app before.<br>The researcher takes consent.	
	**Exercise delivery**	Exercise delivery is the same across intervention and control, but differs dependent on site (see 4.What). Exercise is delivered by the health professional either in the home or within a group.	
	**Motivational input**	**INTERVENTION**	**CONTROL**
		Patient carry out goal setting session with health professional structured by the use of the 'Motivate Me' app and receive a prescribed programme on their app and an exercise booklet.<br><br>Patient will receive prompts and messages through the 'my activity programme app' at home on the days they have planned to exercise.<br><br>Patients will also receive verbal feedback when they see the health professional.	Patient given a prescribed exercise programme and some informal goal setting is carried out as part of the session. They receive a home exercise booklet.<br><br>Patients will only receive verbal feedback when they see the health professional
**7. Where**	The intervention is delivered across several community venues and health centres venues and in patients' homes in Manchester City and Trafford.		
**8. When and how much**		**Intervention arm**	**Control arm**
	Research Team	1 visit to set- up the phone after randomisation	1 visit to set- up the phone after randomisation

For peer review only

		1 visit the week after the rehabilitation programme has commenced<br><br>Phone calls x 6	1 visit the week after the rehabilitation programme has commenced<br><br>Phone calls x 6
	Health Professional	2 x structured goal setting session (baseline and at discharge).<br><br>Weekly face to face exercise intervention (ranging from 6-15 contacts).<br><br>Weekly feedback message received through app until discharge (maximum of 26).	2 x goal setting session (baseline and at discharge).<br><br>Weekly face to face exercise intervention (ranging from 6-15 contacts).
	Smartphone	Automated messages and prompts x 3 on the day they have scheduled to exercise.	
**9. Tailoring**	Rehabilitation programme	The number of home based visits to each patient may differ across both recruitment sites and across both control and intervention groups dependent on patient need.<br><br>The exercise programme delivered will be tailored to each individual patient across both sites and across both control and intervention groups.<br><br>Both sites will send the weekly feedback message until patient discharge (this could be at 8 weeks at Trafford and 6 months at Manchester city).	
	Motivational messages	Because the exercise programme is tailored to the individual, health professionals may schedule for the patient to exercise 3 times a week on the phone or every day, dependent on preference. Participants will receive messages on the days they have scheduled to exercise some patients may receive more than others.	
**11. How well planned**	Health professional fidelity	The research team will attend the goal-setting sessions for the intervention arm for the first 5 patients at each site and then 1 patient at each site every 2 months.<br><br>All staff have undergone a half-day training session and a follow-up support session in using the smartphone app and trial procedures.	

For peer review only

	Assessment fidelity	Assessors masked to group allocation will follow an assessment standard operating procedure.
	Adherence	1. Adherence is collected through the smartphone apps (control and intervention).<br>2. At baseline, 3 and 6 months though validated questionnaire (EARS).<br>3. Through group exercise attendance records, health professional and instructor delivery records.

Item 10 and 12 N/A.

For peer review only

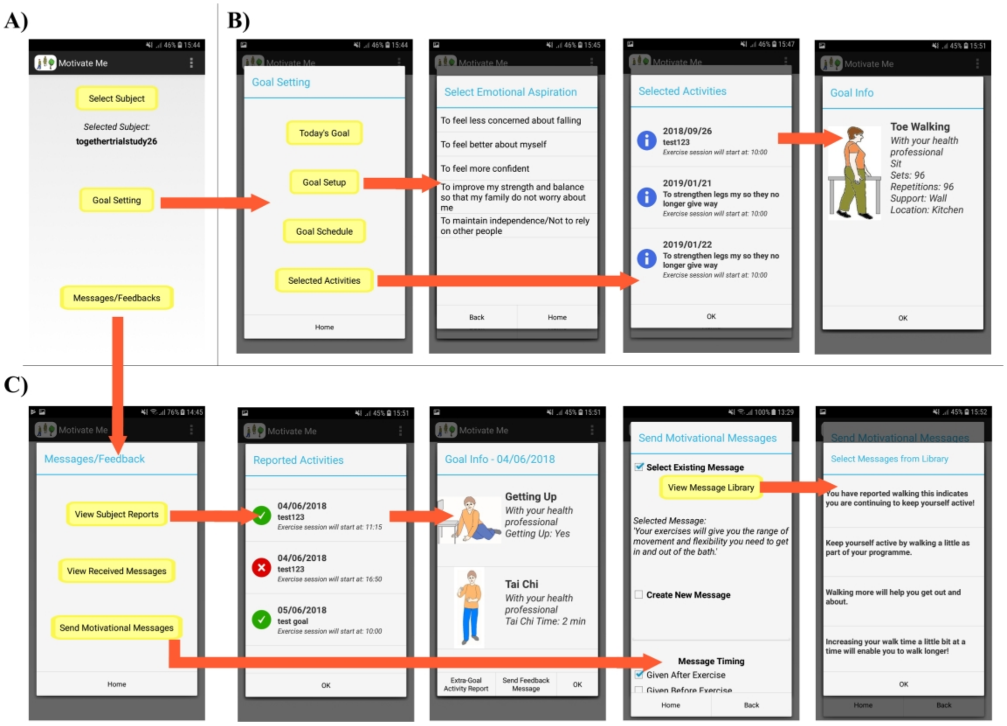
A)
B)
C)
Motivate Me
Select Subject
Selected Subject:
togethertrialstudy26
Goal Setting
Messages/Feedbacks
Today's Goal
Goal Setup
Goal Schedule
Selected Activities
Home
Select Emotional Aspiration
To feel less concerned about falling
To feel better about myself
To feel more confident
To improve my strength and balance so that my family do not worry about me
To maintain independence/Not to rely on other people
Back
Selected Activities
OK
Goal Info
Toe Walking
Messages/Feedback
View Subject Reports
View Received Messages
Send Motivational Messages
Reported Activities
Goal Info - 04/06/2018
Getting Up
Tai Chi
Send Motivational Messages
Select Existing Message
View Message Library
Create New Message
Message Timing
Given After Exercise
Select Messages from Library

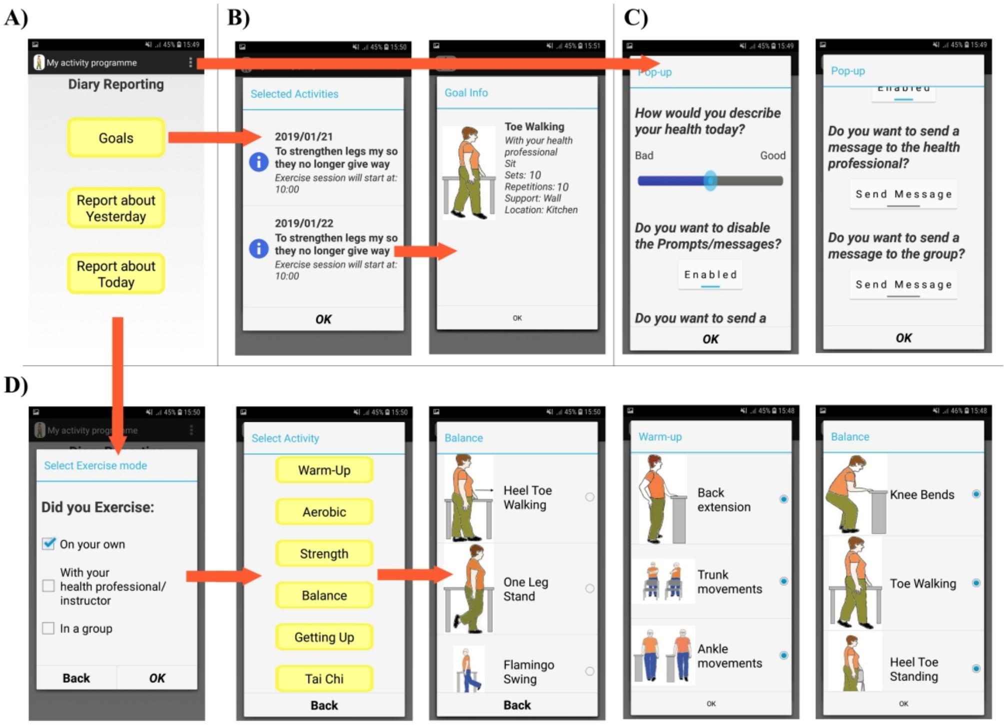
A)
My activity programme
Diary Reporting
Goals
Report about Yesterday
Report about Today
B)
Selected Activities
2019/01/21
To strengthen legs my so they no longer give way
Exercise session will start at: 10:00
2019/01/22
To strengthen legs my so they no longer give way
Exercise session will start at: 10:00
OK
Goal Info
Toe Walking
With your health professional
Sit
Sets: 10
Repetitions: 10
Support: Wall
Location: Kitchen
OK
C)
Pop-up
How would you describe your health today?
Bad
Good
Do you want to disable the Prompts/messages?
Enabled
Do you want to send a
OK
Pop-up
Do you want to send a message to the health professional?
Send Message
Do you want to send a message to the group?
Send Message
OK
D)
Select Exercise mode
Did you Exercise:
On your own
With your health professional/ instructor
In a group
Back
OK
Select Activity
Warm-Up
Aerobic
Strength
Balance
Getting Up
Tai Chi
Back
Balance
Heel Toe Walking
One Leg Stand
Flamingo Swing
Back
Warm-up
Back extension
Trunk movements
Ankle movements
OK
Balance
Knee Bends
Toe Walking
Heel Toe Standing
OK

STANDARD PROTOCOL ITEMS: RECOMMENDATIONS FOR INTERVENTIONAL TRIALS

SPIRIT 2013 Checklist: Recommended items to address in a clinical trial protocol and related documents*

Section/item	Item No	Description	Addressed on page number
**Administrative information**			
Title	1	Descriptive title identifying the study design, population, interventions, and, if applicable, trial acronym	Page 1
Trial registration	2a	Trial identifier and registry name. If not yet registered, name of intended registry	Page 2
	2b	All items from the World Health Organization Trial Registration Data Set	Page 6
Protocol version	3	Date and version identifier	Page 2
Funding	4	Sources and types of financial, material, and other support	Page 20
Roles and responsibilities	5a	Names, affiliations, and roles of protocol contributors	Page 1 & 20
	5b	Name and contact information for the trial sponsor	Page 1 & 7
	5c	Role of study sponsor and funders, if any, in study design; collection, management, analysis, and interpretation of data; writing of the report; and the decision to submit the report for publication, including whether they will have ultimate authority over any of these activities	Page 20
	5d	Composition, roles, and responsibilities of the coordinating centre, steering committee, endpoint adjudication committee, data management team, and other individuals or groups overseeing the trial, if applicable (see Item 21a for data monitoring committee)	Page 18

For peer review only

**Introduction**			
Background and rationale	6a	Description of research question and justification for undertaking the trial, including summary of relevant studies (published and unpublished) examining benefits and harms for each intervention	Pages 4-5
	6b	Explanation for choice of comparators	Page 5
Objectives	7	Specific objectives or hypotheses	Page 5
Trial design	8	Description of trial design including type of trial (eg, parallel group, crossover, factorial, single group), allocation ratio, and framework (eg, superiority, equivalence, noninferiority, exploratory)	Page 5
**Methods: Participants, interventions, and outcomes**			
Study setting	9	Description of study settings (eg, community clinic, academic hospital) and list of countries where data will be collected. Reference to where list of study sites can be obtained	Page 6-8
Eligibility criteria	10	Inclusion and exclusion criteria for participants. If applicable, eligibility criteria for study centres and individuals who will perform the interventions (eg, surgeons, psychotherapists)	Page 7-8
Interventions	11a	Interventions for each group with sufficient detail to allow replication, including how and when they will be administered	Pages 9-12 and supplementary table.
	11b	Criteria for discontinuing or modifying allocated interventions for a given trial participant (eg, drug dose change in response to harms, participant request, or improving/worsening disease)	Pages 18-19
	11c	Strategies to improve adherence to intervention protocols, and any procedures for monitoring adherence (eg, drug tablet return, laboratory tests)	Pages 14 & 16
	11d	Relevant concomitant care and interventions that are permitted or prohibited during the trial	Pages 11
Outcomes	12	Primary, secondary, and other outcomes, including the specific measurement variable (eg, systolic blood pressure), analysis metric (eg, change from baseline, final value, time to event), method of aggregation (eg, median, proportion), and time point for each outcome. Explanation of the clinical relevance of chosen efficacy and harm outcomes is strongly recommended	Pages 11-16

For peer review only - http://bmjopen.bmj.com/site/about/guidelines.xhtml

Participant timeline	13	Time schedule of enrolment, interventions (including any run-ins and washouts), assessments, and visits for participants. A schematic diagram is highly recommended (see Figure)	Page 12
Sample size	14	Estimated number of participants needed to achieve study objectives and how it was determined, including clinical and statistical assumptions supporting any sample size calculations	Page 8
Recruitment	15	Strategies for achieving adequate participant enrolment to reach target sample size	Page 8

Methods: Assignment of interventions (for controlled trials)

Allocation:			
Sequence generation	16a	Method of generating the allocation sequence (eg, computer-generated random numbers), and list of any factors for stratification. To reduce predictability of a random sequence, details of any planned restriction (eg, blocking) should be provided in a separate document that is unavailable to those who enrol participants or assign interventions	Page 8
Allocation concealment mechanism	16b	Mechanism of implementing the allocation sequence (eg, central telephone; sequentially numbered, opaque, sealed envelopes), describing any steps to conceal the sequence until interventions are assigned	Page 8
Implementation	16c	Who will generate the allocation sequence, who will enrol participants, and who will assign participants to interventions	Page 8
Blinding (masking)	17a	Who will be blinded after assignment to interventions (eg, trial participants, care providers, outcome assessors, data analysts), and how	Page 8
	17b	If blinded, circumstances under which unblinding is permissible, and procedure for revealing a participant's allocated intervention during the trial	N/A only the assessor and statistician is blinded

Methods: Data collection, management, and analysis

For peer review only - http://bmjopen.bmj.com/site/about/guidelines.xhtml

Data collection methods	18a	Plans for assessment and collection of outcome, baseline, and other trial data, including any related processes to promote data quality (eg, duplicate measurements, training of assessors) and a description of study instruments (eg, questionnaires, laboratory tests) along with their reliability and validity, if known. Reference to where data collection forms can be found, if not in the protocol	Pages 11-16.
	18b	Plans to promote participant retention and complete follow-up, including list of any outcome data to be collected for participants who discontinue or deviate from intervention protocols	Page 16
Data management	19	Plans for data entry, coding, security, and storage, including any related processes to promote data quality (eg, double data entry; range checks for data values). Reference to where details of data management procedures can be found, if not in the protocol	Page 17
Statistical methods	20a	Statistical methods for analysing primary and secondary outcomes. Reference to where other details of the statistical analysis plan can be found, if not in the protocol	Page 17
	20b	Methods for any additional analyses (eg, subgroup and adjusted analyses)	Page 17
	20c	Definition of analysis population relating to protocol non-adherence (eg, as randomised analysis), and any statistical methods to handle missing data (eg, multiple imputation)	Page 17
**Methods: Monitoring**			
Data monitoring	21a	Composition of data monitoring committee (DMC); summary of its role and reporting structure; statement of whether it is independent from the sponsor and competing interests; and reference to where further details about its charter can be found, if not in the protocol. Alternatively, an explanation of why a DMC is not needed	Page 18
	21b	Description of any interim analyses and stopping guidelines, including who will have access to these interim results and make the final decision to terminate the trial	N/A
Harms	22	Plans for collecting, assessing, reporting, and managing solicited and spontaneously reported adverse events and other unintended effects of trial interventions or trial conduct	Page 18
Auditing	23	Frequency and procedures for auditing trial conduct, if any, and whether the process will be independent from investigators and the sponsor	Page 18
**Ethics and dissemination**			

Research ethics approval	24	Plans for seeking research ethics committee/institutional review board (REC/IRB) approval	Page 17
Protocol amendments	25	Plans for communicating important protocol modifications (eg, changes to eligibility criteria, outcomes, analyses) to relevant parties (eg, investigators, REC/IRBs, trial participants, trial registries, journals, regulators)	N/A
Consent or assent	26a	Who will obtain informed consent or assent from potential trial participants or authorised surrogates, and how (see Item 32)	Page 8
	26b	Additional consent provisions for collection and use of participant data and biological specimens in ancillary studies, if applicable	N/A
Confidentiality	27	How personal information about potential and enrolled participants will be collected, shared, and maintained in order to protect confidentiality before, during, and after the trial	Page 18
Declaration of interests	28	Financial and other competing interests for principal investigators for the overall trial and each study site	Page 20
Access to data	29	Statement of who will have access to the final trial dataset, and disclosure of contractual agreements that limit such access for investigators	N/A As not full trial
Ancillary and post-trial care	30	Provisions, if any, for ancillary and post-trial care, and for compensation to those who suffer harm from trial participation	Page 18
Dissemination policy	31a	Plans for investigators and sponsor to communicate trial results to participants, healthcare professionals, the public, and other relevant groups (eg, via publication, reporting in results databases, or other data sharing arrangements), including any publication restrictions	Page 2
	31b	Authorship eligibility guidelines and any intended use of professional writers	________
	31c	Plans, if any, for granting public access to the full protocol, participant-level dataset, and statistical code	N/A

Appendices

Informed consent materials	32	Model consent form and other related documentation given to participants and authorised surrogates	N/A

For peer review only

Biological specimens	33	Plans for collection, laboratory evaluation, and storage of biological specimens for genetic or molecular analysis in the current trial and for future use in ancillary studies, if applicable	N/A

*It is strongly recommended that this checklist be read in conjunction with the SPIRIT 2013 Explanation & Elaboration for important clarification on the items. Amendments to the protocol should be tracked and dated. The SPIRIT checklist is copyrighted by the SPIRIT Group under the Creative Commons "Attribution-NonCommercial-NoDerivs 3.0 Unported" license.

For peer review only
